# Dietary Fiber-Associated Differences in Gut Microbial Community Composition and Predicted Short-Chain Fatty Acid-Related Functional Potential: An In Silico Re-Analysis of 16S rRNA Data

**DOI:** 10.3390/ijms27177851

**Published:** 2026-09-02

**Authors:** Shaza N. Alkhatib

**Affiliations:** Department of Biological Sciences, College of Sciences and Arts Khulais, University of Jeddah, Jeddah 21959, Saudi Arabia; snalkhatib@uj.edu.sa

**Keywords:** dietary fiber, gut microbiota, SCFAs, predicted functional potential, in silico re-analysis, microbiome composition

## Abstract

Dietary fiber shapes both gut microbial community structure and the fermentable substrates available to resident taxa, yet how fiber deprivation reshapes taxonomic representation and predicted functional potential remains incompletely resolved. We performed an in silico re-analysis of publicly deposited 16S rRNA gene data from 15 female C57BL/6 mice distributed across three dietary cohorts—fiber-replete (F), no-fiber (NF), and no-fiber with exogenous short-chain fatty acid supplementation (NF-SCFA)—using processed taxonomic profiles and PICRUSt-derived KO/EC and KEGG pathway representations. No new animals, sequencing, enzyme assays, metabolomics, or direct SCFA measurements were generated. The F cohort exhibited the highest alpha-diversity summaries (observed features, Shannon, Simpson) and higher read-count representation for fiber-associated taxa, including Clostridium and Bifidobacterium, whereas NF profiles showed elevated representation of *Ligilactobacillus* in several samples. Beta-diversity analyses (PERMANOVA, PCoA) revealed that F communities were compositionally distinct from both fiber-deprived cohorts, while NF and NF-SCFA remained similar to one another. Among 52 differentially represented KO/EC features mapped across 31 KEGG pathway groups, 19 were F-preferential and 33 NF-preferential, spanning carbohydrate, pyruvate, propanoate, and butanoate metabolism. Critically, the NF-SCFA cohort failed to recapitulate the F-like taxonomic or functional pattern, indicating that exogenous SCFA supplementation alone does not restore fiber-associated community structure. These findings describe associations and predicted functional potential rather than demonstrated enzyme activity, metabolite concentration, or SCFA output, and should be interpreted as hypothesis-generating. The results identify candidate taxa and pathways warranting direct biochemical, transcriptomic, and strain-resolved validation before conclusions about fiber-dependent SCFA biosynthesis can be established.

## 1. Introduction

The human gut microbiota is a complex and dynamic ecosystem that contributes to nutrient transformation, immune regulation, epithelial integrity, and metabolic homeostasis. Perturbations in community composition and function, often referred to as dysbiosis, have been associated with inflammatory bowel disease, obesity, type 2 diabetes, cardiovascular disease, and neurodegenerative conditions [1]. These associations provide biological context for studying dietary influences, but they do not mean that a taxonomic difference in the present 16S-derived dataset demonstrates a disease mechanism or a host outcome.

Short-chain fatty acids (SCFAs), especially butyrate and propionate, are microbial metabolites that participate in intestinal barrier regulation, immune signaling, and energy homeostasis [1,2,3,4,5]. When SCFA concentrations become imbalanced, associations with metabolic, cardiovascular, and inflammatory disease processes have been reported [2,6,7,8]. SCFAs can also influence B-cell function through epigenetic mechanisms. In particular, butyrate and propionate can inhibit histone deacetylases (HDACs), increasing acetylation at regulatory regions of microRNA host genes and altering microRNAs that target *Aicda* and *Prdm1* transcripts [9,10]. HDACs normally remove acetyl groups from histones, promoting chromatin condensation and transcriptional silencing; inhibition of this activity can increase chromatin accessibility and change gene expression [11,12]. The resulting effects on activation-induced cytidine deaminase (AID) and Blimp-1 are relevant to class-switch recombination, somatic hypermutation, and plasma-cell differentiation [9]. *Prdm1* also contributes to germinal-center exit and repression of genes that maintain the germinal-center B-cell state [13,14], while its dysregulation has been linked to immune disorders and autoimmunity [15]. These observations establish why SCFA biology is relevant background for the present study. They do not, however, establish that SCFAs, B-cell responses, or host disease processes were measured in this re-analysis; the present work evaluates only 16S-derived taxonomic profiles and inferred functional potential.

In animals consuming diets rich in fiber, gut microorganisms encounter fermentable substrates such as inulin, resistant starch, pectin, arabinoxylan, and beta-glucans, which can support carbohydrate degradation and fermentation-associated functions [16,17,18]. Fiber-associated taxa, including members of *Clostridium*, *Lactobacillus*, *Bifidobacterium*, *Roseburia*, *Oscillibacter*, *Bacteroides*, and *Prevotella*, have been discussed in relation to these processes, although genus-level membership does not establish a common strain-level metabolism [19,20,21]. Carbohydrate-active enzymes and related pathways can convert complex polysaccharides to smaller substrates that enter central metabolic routes, with pyruvate serving as a metabolic junction for several fermentation pathways [22,23,24,25]. Acetate-, propionate-, and butyrate-associated routes include multiple alternative reactions and cross-feeding relationships, so a pathway map should not be read as a direct measurement of product formation [25,26,27,28,29,30]. The availability of fiber may therefore influence which organisms and predicted functional features are represented in a community, whereas the absence of fiber may increase the relative representation of features associated with alternative carbohydrate, amino-acid, or host-derived substrates [31,32,33]. Dietary fiber and gut microbial metabolism have also been linked to broader host energy and immune processes in prior work [1,25,34,35]. The current analysis uses this literature as biological context only. PICRUSt uses 16S rRNA gene profiles and reference-genome information to infer the representation of KEGG orthologs and enzyme-associated features; those outputs describe predicted functional potential and do not measure transcripts, proteins, reaction rates, metabolic flux, metabolites, or SCFAs [35,36,37,38].

We therefore re-analyzed deposited 16S rRNA gene data from three dietary cohorts of female C57BL/6 mice: F, NF, and NF-SCFA, with five samples in each cohort. We compared alpha- and beta-diversity summaries, taxonomic read-count profiles, PICRUSt-inferred KO/EC profiles, and mapped KEGG pathway representations, and asked whether the NF-SCFA profile resembled the F profile in the supplied evidence. The analysis was computational and hypothesis-generating; no new animals, samples, sequencing, or wet-lab measurements were performed, and no direct inference about SCFA concentrations or host responses was attempted.

## 2. Results

This research was designed to characterize the nutritionally responsive metabolic flexibility of the gut microbiota in female C57BL/6 mice by systematically quantifying community-level microbial abundance patterns and their associated metabolic capacity for short-chain fatty acid (SCFA) production. To clarify the dietary basis for downstream microbiome and metabolic analyses, female C57BL/6 mice were maintained on one of three nutritional regimens: fiber-replete (F), fiber-devoid (NF), or fiber-devoid supplemented with exogenous short-chain fatty acids (NF-SCFA). The product identities and fiber content of these diets, as originally reported by Sanchez et al. (2020) [9], are summarized in Table S1. The F diet corresponded to Teklad Mouse/Rat Sterilizable Diet LM-485, containing approximately 18% crude fiber, while the NF diet corresponded to Harlan TD.00129 AIN-93G No Cellulose, containing 0% fiber. Notably, the NF-SCFA regimen did not represent a third, independently formulated solid diet; rather, it consisted of the same NF diet supplemented with an exogenous SCFA cocktail delivered via drinking water, comprising a 20.0 mg mL^−1^ tributyrin emulsion, 140 mM sodium butyrate, and 150 mM sodium propionate, adjusted to pH 7.4. Formulation fields not explicitly reported in the original source publication are indicated in Table S1 as “Not reported in Sanchez et al. (2020)”. Because complete non-fiber macronutrient composition was not fully established in the cited source, potential differences in energy density, protein, carbohydrate, and micronutrient content between diets cannot be excluded as confounding variables in the interpretation of downstream microbiome and metabolic findings; accordingly, no manufacturer-derived values were inferred or supplemented beyond those explicitly reported in the original study.

**Figure 1:**
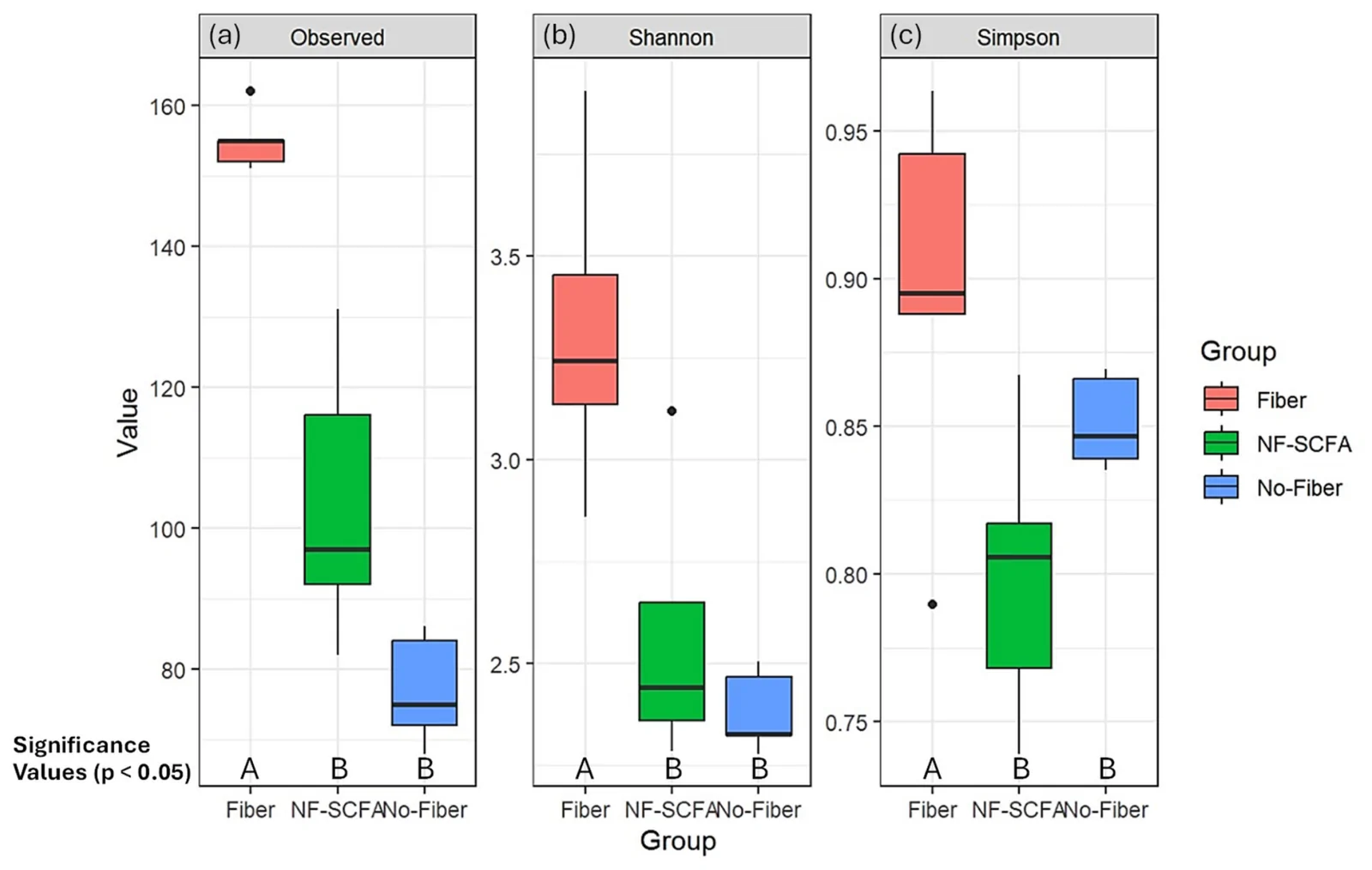
Comparison of alpha-diversity indices—observed features (**a**), Shannon index (**b**), and Simpson index (**c**)—among gut microbiota communities in female C57BL/6 mice assigned to the fiber-replete (F), no-fiber (NF), and no-fiber plus exogenous short-chain fatty acid (NF-SCFA) cohorts. “Observed features” denotes the number of detected operational taxonomic units or species-level features and therefore reflects richness in the processed 16S-derived profiles. Shannon combines richness and evenness, whereas Simpson gives greater weight to the distribution of common features. The displayed values are cohort-level summaries derived from deposited 16S rRNA gene data; they are not absolute bacterial-load measurements and do not establish the mechanism responsible for any difference. The three indices answer related but distinct questions about richness and evenness, and their interpretation is limited to the processed sample profiles.

Alpha-diversity analysis (Figure 1) revealed that the fiber-replete (F) cohort demonstrated the highest diversity metrics across observed species (Figure 1a), Shannon (Figure 1b), and Simpson (Figure 1c) indices, reflecting the capacity of fermentable polysaccharides to sustain a taxonomically rich and evenly distributed microbial community. Conversely, the fiber-deprived (NF) cohort exhibited markedly diminished alpha diversity, indicative of reduced ecological complexity and loss of specialized fiber-degrading taxa, while the fiber-deprived with exogenous SCFA supplementation (NF-SCFA) cohort showed alpha-diversity metrics substantially attenuated relative to F but comparable to NF, underscoring that exogenous SCFA provision fails to recapitulate the substrate-dependent microbial ecological complexity of fiber-replete conditions.

**Figure 2:**
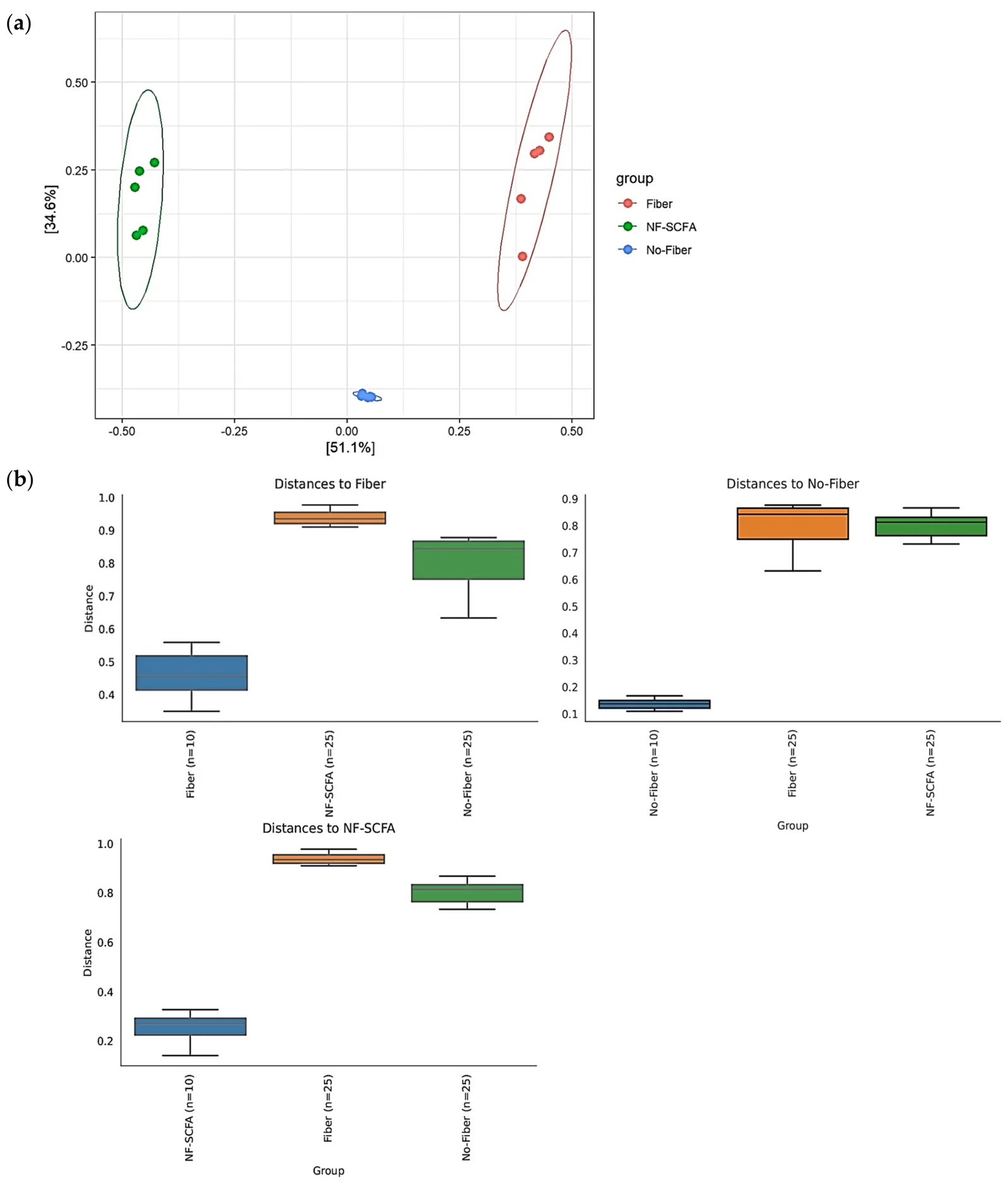
Dietary effects on gut microbial community composition in female C57BL/6 mice. (**a**) Three-dimensional principal coordinate analysis (PCoA) of Bray–Curtis dissimilarity matrices, depicting compositional divergence among intestinal microbial communities in mice fed fiber-replete (F), fiber-devoid (NF), or fiber-devoid diets supplemented with exogenous short-chain fatty acids (NF-SCFA). Ellipses denote 95% confidence intervals surrounding each dietary cohort’s centroid; (**b**) permutational multivariate analysis of variance (PERMANOVA) applied to pairwise Bray–Curtis dissimilarities across the three dietary cohorts, evaluating the magnitude and statistical significance of diet-associated compositional differences. Each cohort comprised n = 5 animals; accordingly, the within-group comparisons in panel (**b**) reflect n = 10 pairwise distances per cohort (C(5,2)), while the between-group comparisons reflect n = 25 pairwise distances per cohort pair (5 × 5).

Principal coordinate analysis (PCoA) ordination (Figure 2a) and PERMANOVA analysis of Bray–Curtis dissimilarity (Figure 2b) both demonstrated that the fiber-replete (F) cohort exhibits compositionally distinct microbial community architecture substantially separated from both fiber-deprived cohorts, with NF and NF-SCFA cohorts showing compositional similarity, thereby indicating that exogenous SCFA supplementation does not substantially restore the microbial community structure characteristic of fiber-replete conditions and highlighting the irreplaceable role of fermentable polysaccharides in orchestrating optimal microbiota diversity, composition, and functional capacity. To statistically substantiate the separation visualized in Figure 2, PERMANOVA was performed on the Bray–Curtis distance matrix (999 permutations, QIIME 2 2026.4), with Benjamini–Hochberg correction across pairwise comparisons. The overall PERMANOVA was highly significant (pseudo-F = 31.98, *p* = 0.001, n = 15), and all pairwise comparisons remained significant after correction (BH q = 0.012 in each case), confirming that each dietary cohort harbors a compositionally distinct gut microbial community. These results were corroborated by ANOSIM (R = 1.0, *p* = 0.001) and Jaccard-based PERMANOVA (pseudo-F = 10.83, *p* = 0.001), providing convergent support for the PCoA separation shown in Figure 2a; details are reported in Table S2.

**Figure 3:**
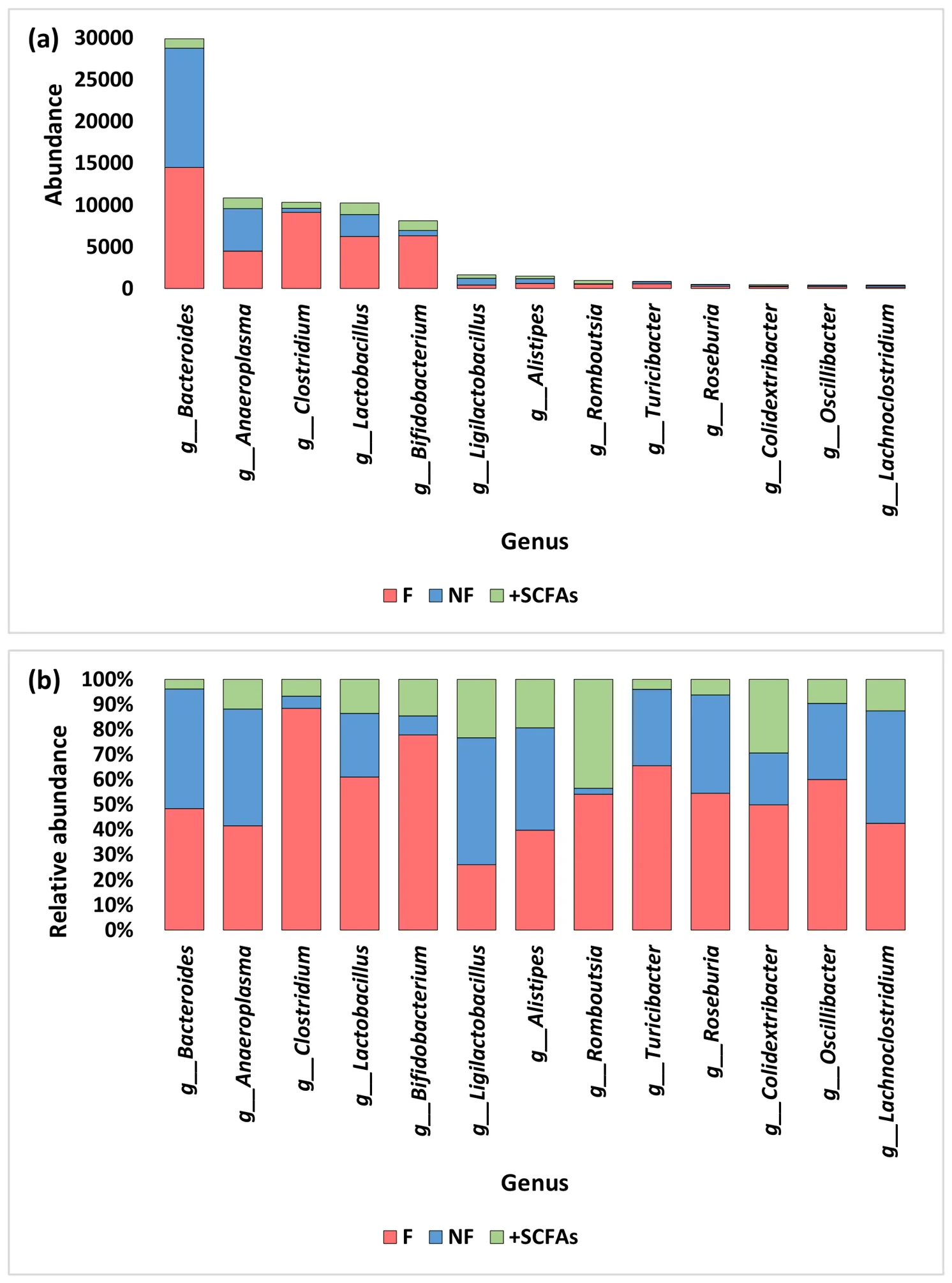
Abundance (**a**) and relative abundance (**b**) of the most prevalent bacterial genera in the gut microbiota of female C57BL/6 mice subjected to fiber-replete (F), fiber-devoid (NF), and fiber-devoid with exogenous short-chain fatty acid supplementation (+SCFA) dietary regimens. A rigorous abundance criterion of ≥100 gene query enumeration was applied to identify highly abundant microbial taxa demonstrating significant prevalence across the three dietary cohorts. Additional details are provided in Table S3.

Figure 3 and Table S3 contain 16S rRNA gene read counts for 13 selected genera displayed across the three cohorts. The table applies the documented threshold of at least 100 gene-query counts to focus the comparison on genera with substantial representation in the supplied profile. F-associated examples included higher counts for several fiber-associated taxa, including *Clostridium* and *Bifidobacterium*, whereas *Ligilactobacillus* was higher in several NF samples. Individual accessions varied considerably, and the table entries do not establish a common abundance ranking for every animal or cohort. Because the values are read-count-derived and no independent total bacterial-load measurement was documented, they should be described as gene-query counts or relative representation among the displayed taxa, not as absolute bacterial abundance. The genus-level comparison is descriptive and does not support unreported significance or strain-level functional claims. The selected genera should therefore be viewed as a reporting subset rather than a complete description of the intestinal community. Counts can vary between accessions within the same cohort, and a higher count for one genus does not imply a corresponding increase in total bacterial load or in a functional product. The comparison also does not provide a basis for assigning causality to a particular dietary constituent.

**Figure 4:**
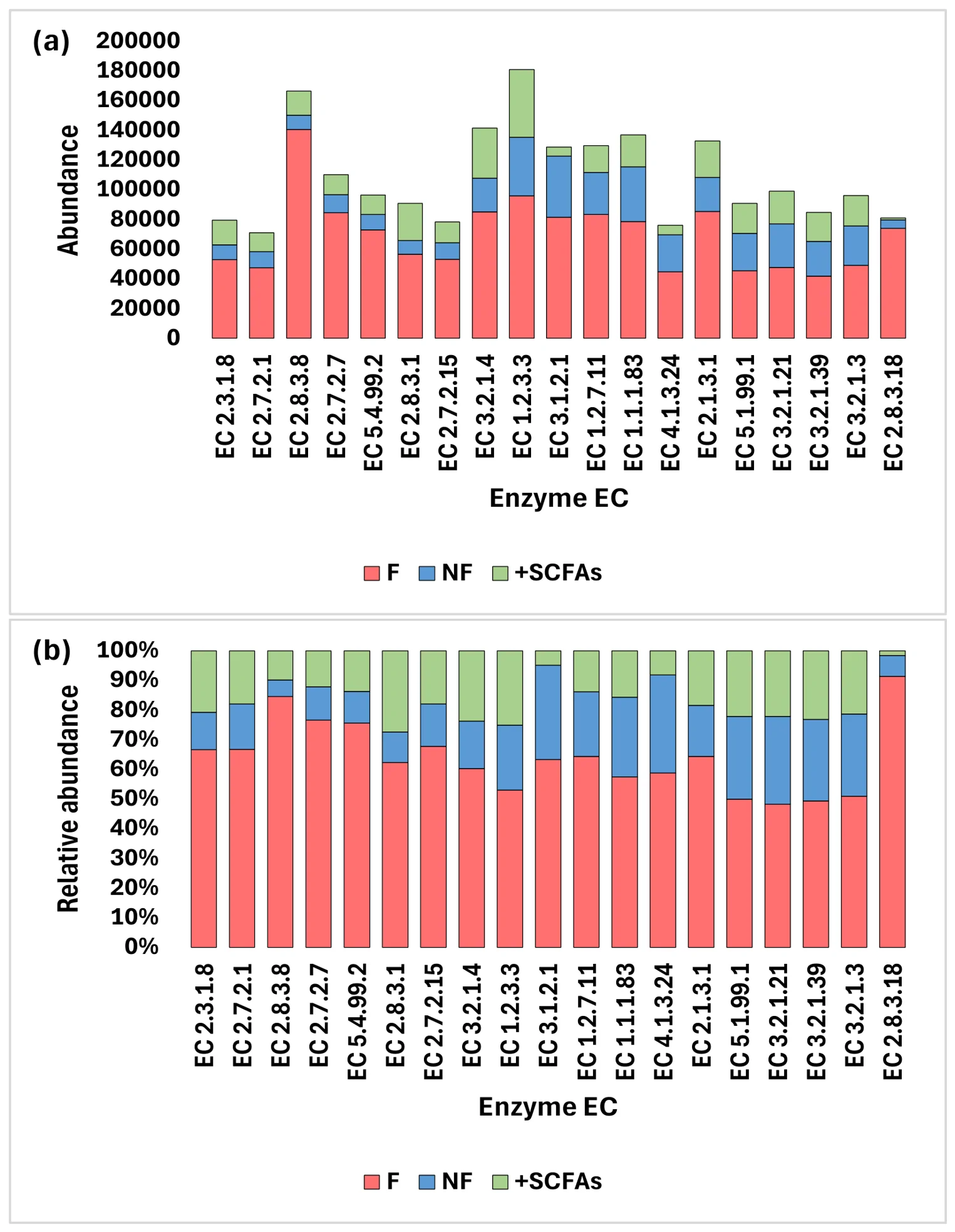
Abundance (**a**) and relative abundance (**b**) of preferential KEGG enzymes or enzymatic constituents within the gut microbiome of female C57BL/6 mice showing high prevalent enzymes in fiber-replete (F) cohort compared to those fed a fiber-devoid (NF) diet. Comprehensive details for enzymatic classifications, abundance, and pathway mapping are available in Tables S4 and S5. Aligning with the results of enzyme analysis, three pathways were highly enriched in F cohort relative to both other cohorts (Figure S32 and Tables S6 and S7), while 22 pathways were most enriched in the NF cohort (Figure S33 and Tables S6 and S7). The six residual pathways exhibiting pronounced enrichment of enzymes or enzymatic subunits common to both the fiber-replete (F) and fiber-deprived (NF) cohorts were likewise subjected to comprehensive metabolic interrogation (Figure S34 and Tables S6 and S7). The concurrent enrichment observed across these three pathway groups is plausibly attributable to the elevated abundance of their constituent enzymes within both murine cohorts.

**Figure 5:**
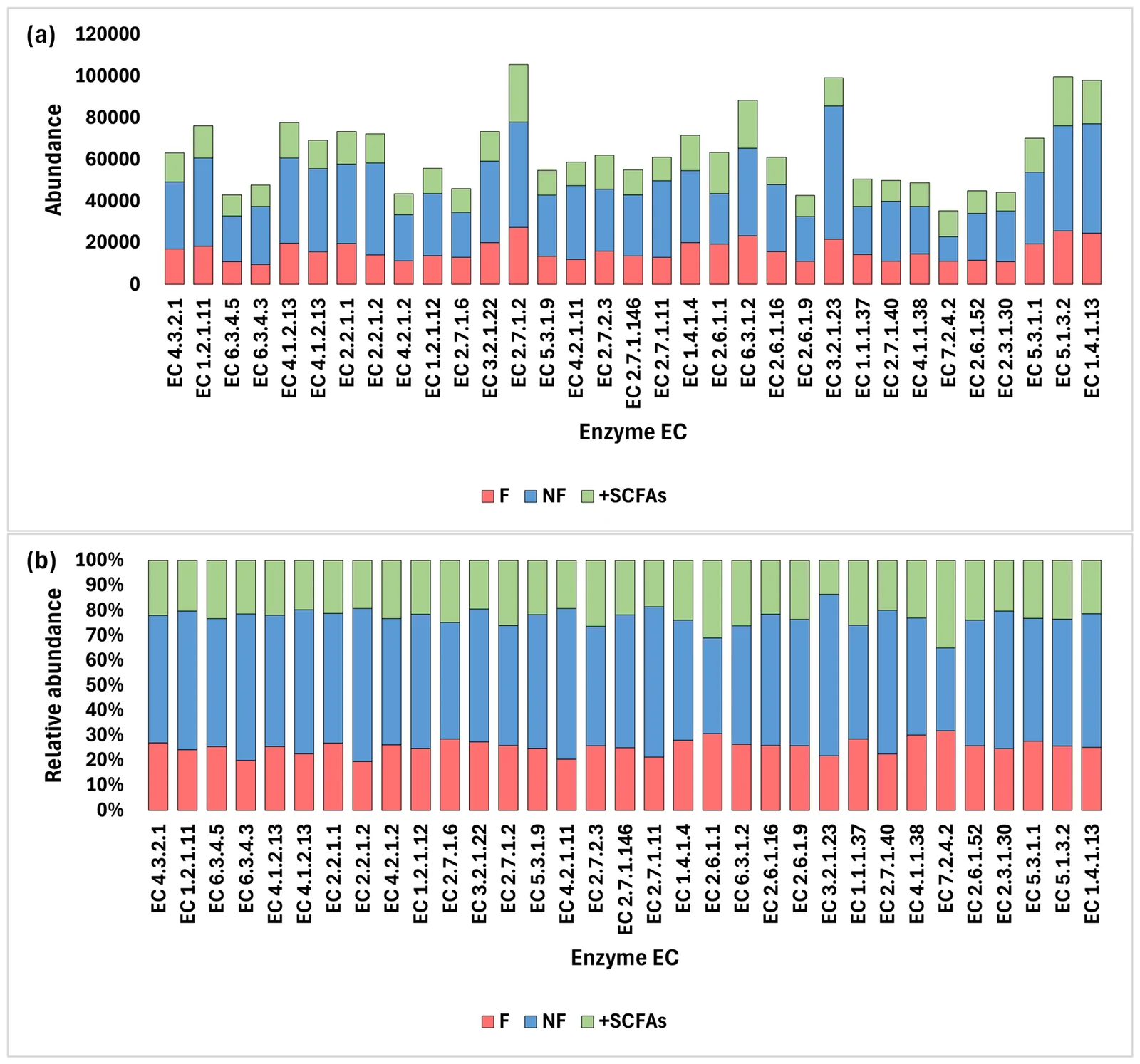
Abundance (**a**) and relative abundance (**b**) of preferential KEGG enzymes or enzymatic constituents within the gut microbiome of female C57BL/6 mice showing highly prevalent enzymes in the fiber-replete (F) cohort compared to those fed a fiber-devoid diet supplemented with exogenous SCFAs (NF-SCFA). Comprehensive details for enzymatic classifications, abundance, and pathway mapping are available in Tables S4–S7.

Tables S4 and S5 were examined to identify KO/EC features with differing predicted representation among the F, NF, and NF-SCFA cohorts, yielding 19 F-preferential and 33 NF-preferential features that map to 31 KEGG pathway groups (Figures S1–S31; Table S6). Figures S32–S34 present the corresponding PICRUSt-predicted and relative functional representations of these preferential pathways: three pathways enriched in F over both other cohorts (Figure S32), 22 enriched in NF (Figure S33), and six shared between F and NF relative to NF-SCFA (Figure S34), with full detail in Tables S6 and S7. As with the earlier pathway maps, these figures capture only inferred KOEC/KEGG representation drawn from the applied filtering and comparison criteria, not actual enzyme concentrations, catalytic rates, transcript or protein levels, or confirmed pathway activity. Figure 4 and Figure 5 should likewise be read as PICRUSt-based predicted functional profiles that identify candidate associations for future validation rather than direct biochemical measurements. Throughout, “preferential” and “enriched” serve strictly as descriptive labels for the comparative profiles rather than indicators of absolute abundance, and the reported pathway counts are preserved mainly to keep the source tables auditable and to flag candidate functional groups for subsequent experimental testing.

The F-associated KO/EC examples shown in Figure 4 are PICRUSt-predicted functional representations from the processed tables. They include features mapped to carbohydrate, pyruvate, propanoate, and butanoate pathway groups, which together provide a computational view of functions that may be associated with fiber-supported community structure. The representation of glucose-related and pyruvate-related features does not demonstrate glucose utilization, conversion of pyruvate to acetate, or any other product-forming reaction in the animals. Likewise, the presence of a predicted KO or EC annotation cannot distinguish gene content from transcription, translation, enzyme activity, substrate availability, or catalytic flux. The F-associated pattern is therefore reported as a difference in predicted representation that can guide targeted measurement, not as evidence of enhanced biochemical production. This distinction is particularly important for the pathway labels because a KEGG annotation can be shared by organisms with different physiological roles.

Figure 5 presents the same set of F-preferential KO/EC features shown in Figure 4, but benchmarked against the NF-SCFA cohort rather than the NF cohort. This complementary comparison addresses whether exogenous SCFA supplementation, in the absence of dietary fiber, shifts the predicted functional profile toward the F-associated pattern. As with Figure 4, the values plotted are PICRUSt-inferred representations and do not constitute direct measurements of enzyme activity, metabolite concentration, or catalytic flux. If the F-preferential features remain comparably distinct from NF-SCFA as they are from NF, this would indicate that exogenous SCFA provision alone does not recapitulate the fiber-associated functional signature reflected in Figure 4. Conversely, any convergence between F and NF-SCFA profiles in this comparison would need to be interpreted cautiously, since PICRUSt annotations reflect predicted genomic potential rather than confirmed pathway operation, and a shared KO/EC annotation across cohorts does not establish that the same reaction is occurring at comparable rates or is being carried out by the same taxa. Together, Figure 4 and Figure 5 frame the F-preferential functional signature against both fiber-deprived comparator groups, allowing the dietary-fiber-specific and SCFA-specific contributions to predicted functional representation to be considered separately.

Figure S35 illustrates the discrete metabolic architectures spanning multiple integrated KEGG pathways that collectively govern acetate biosynthesis, emphasizing how variations in substrate availability and microbial composition dictate divergent metabolic strategies for acetate production across the two dietary cohorts. In the fiber-deprived (NF) mice, the substantial accumulation of metabolic intermediates denotes an adaptive reconfiguration of the gut microbiota favoring the exploitation of alternative, non-polysaccharide carbon sources—specifically proteins, endogenous monosaccharides, and notably nucleotides—via glycolytic and proteolytic pathways that principally converge on pyruvate biosynthesis. Conversely, the fiber-replete (F) mice demonstrate an augmented enzymatic capacity to channel pyruvate toward acetate synthesis via core catalytic components of the “Pyruvate metabolism” pathway, indicative of a microbiota-mediated enhancement of the key biosynthetic framework driven by dietary fiber availability. Within this metabolic framework, glucose demonstrated consistent and substantial enrichment irrespective of fiber status, underscoring its central function as the principal glycolytic substrate across the divergent enzymatic configurations and substrate affinities characterizing each dietary cohort, while simultaneously emphasizing the substrate-dependent modalities underlying glucose biosynthesis.

Under fiber-deprived conditions, the marked accumulation of downstream intermediates—such as D-ribose-5-phosphate, D-galactose, Fumarate, fructose-1P, glutamine, and glutamate—signals a disrupted metabolic continuum, wherein these compounds accumulate instead of traversing the full cascade of reactions necessary for short-chain fatty acid synthesis. This phenomenon reflects the metabolic inefficiency and fragmentation inherent to fermentative pathways that do not rely on polysaccharides, whose substrates remain only partially broken down and therefore cannot support efficient short-chain fatty acid production.

**Figure 6:**
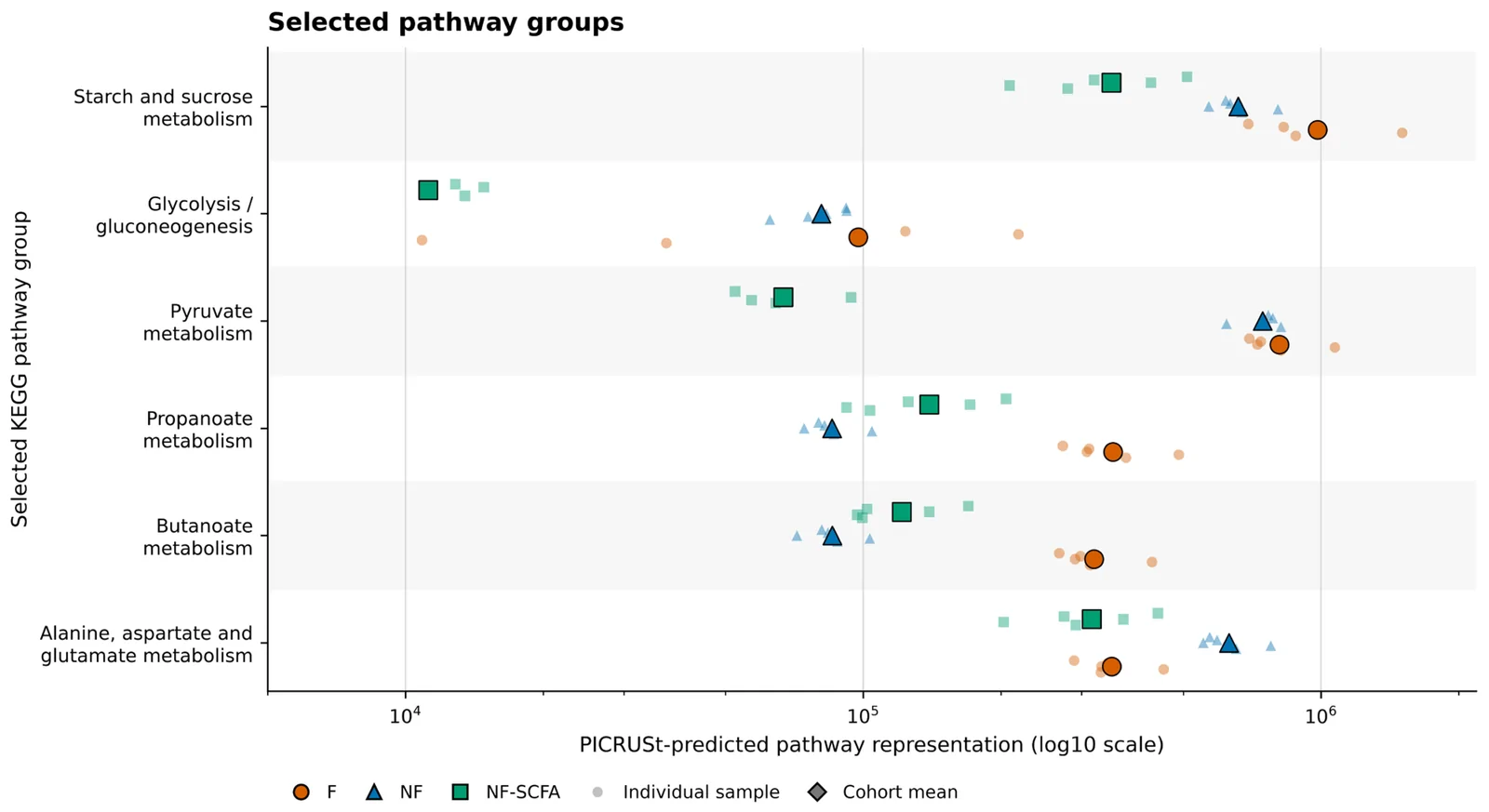
Simplified pathway-level comparison of PICRUSt-predicted functional representation across F, NF, and NF-SCFA cohorts. Six KEGG pathway groups from Table S6 are shown: starch and sucrose metabolism (map00500), glycolysis/gluconeogenesis (map00010), pyruvate metabolism (map00620), propanoate metabolism (map00640), butanoate metabolism (map00650), and alanine, aspartate, and glutamate metabolism (map00250). Small symbols show individual sample values and large outlined symbols show cohort means (n = 5 per cohort); the logarithmic x-axis is used for display, without per-pathway normalization. F showed higher predicted propanoate and butanoate representation, NF showed higher predicted alanine/aspartate/glutamate representation, and NF-SCFA did not reproduce an F-like pattern across the selected pathways. The values are PICRUSt-inferred pathway representations from 16S-derived profiles and are not direct measurements of metabolites, enzymes, transcripts, proteins, reaction rates, flux, or SCFAs. KEGG pathway annotations provide functional context for carbohydrate and SCFA-associated routes [23,25,39,40] but do not establish pathway operation or causal biochemical output. The detailed original pathway-network rendering is shown in Figure S35.

To present the pathway-level pattern in a more accessible form, Figure 6 summarizes six selected KEGG pathway groups from Table S6 as PICRUSt-predicted representation values across the F, NF, and NF-SCFA cohorts. Each cohort contains five samples; small symbols show individual sample values and large symbols show cohort means. The F cohort had higher mean representation of starch and sucrose, propanoate, and butanoate metabolism than NF-SCFA and higher propanoate- and butanoate-associated representation than NF. F and NF showed broadly similar glycolysis/gluconeogenesis and pyruvate representation, whereas NF-SCFA was lower for both. NF showed the highest mean representation of alanine, aspartate, and glutamate metabolism. Across the selected pathway groups, NF-SCFA did not reproduce an F-like pattern. These observations are descriptive differences in inferred functional representation from 16S-derived profiles and do not establish metabolite accumulation, enzyme activity, reaction direction, metabolic flux, or SCFA production [9].

**Figure 7:**
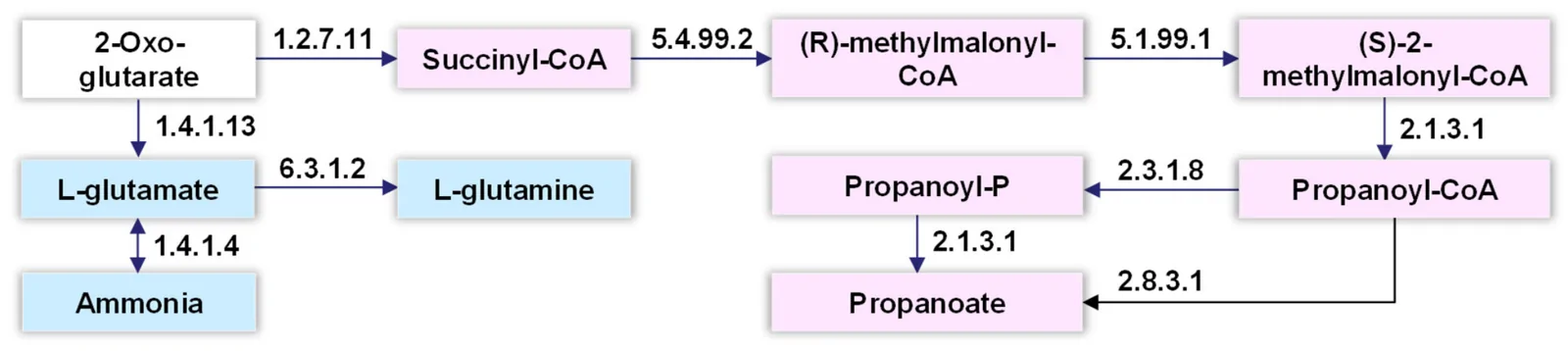
Dietary fiber-dependent orchestration of propanoate biosynthetic specialization in the gut microbiota of female C57BL/6 mice. The diagram delineates the metabolic phenotypic variation exhibited by gut microbiota across three distinct nutritional paradigms: fiber-replete (F), fiber-devoid (NF), and fiber-devoid with exogenous short-chain fatty acid provisioning (NF-SCFA). Color-stratified metabolic nodes identify primary metabolic substrates (white boxes), intermediary metabolic junctions, and terminal fermentation products, each annotated according to their relative quantitative abundance across treatment cohorts. Blue-demarcated boxes represent metabolites manifesting substantially elevated concentrations within the fiber-devoid (NF) microbiota relative to alternative treatment cohorts, whereas pink-demarcated boxes identify metabolites exhibiting preferentially augmented abundance exclusively within the fiber-replete (F) microbiota compared with both fiber-devoid (NF) and fiber-devoid-supplemented (NF-SCFA) cohorts. The hierarchically organized metabolic architecture exemplifies the microbiota’s adaptive metabolic capacity to engage alternative substrates and enzymatic routing mechanisms in response to fiber nutrient bioavailability, with specialized enzymatic programs orchestrating propanoate biosynthesis uniquely tailored to each experimental nutritional condition. Secondary metabolic endpoints accumulated under fiber-devoid conditions—including glutamine and glutamate—represent nitrogenous metabolic intermediates that do not undergo the requisite subsequent transformative steps essential for short-chain fatty acid biosynthesis, thereby illustrating the metabolic incompleteness of non-polysaccharide catabolic pathways in sustaining complete SCFA production. Comprehensive metabolomic characterization and supporting analytical data are detailed in Table S6 and Figures S1, S2, S7, S10, S23–S26 and S30.

Figure 7 demonstrates the dietary fiber-dependent specialization of propanoate biosynthetic avenues within the gut microbiota of female C57BL/6 mice, with a focus on the utilization of substrates such as 2-oxo-glutarate. In fiber-replete mice, the enhanced enzymatic machinery drives more direct routes to propanoate from fermentable fiber-derived substrates, whereas in fiber-deprived conditions the adaptive microbiota leverage nitrogenous intermediates to sustain propanoate production. The 2-Oxo-glutarate serves a dual function: it acts as a central precursor for the biosynthesis of propanoate, as well as glutamine and glutamate, and alternative fermentable intermediates for propanoate production.

## 3. Discussion

Empirical measurements of fecal and tissue SCFA concentrations reported by Sanchez et al. (2020) [9] revealed that fiber-fed animals exhibited elevated SCFA levels relative to those maintained on a no-fiber diet. These biochemical determinations are neither replicated nor re-derived within the present investigation; rather, this work undertakes a secondary interrogation of the identical 15-sample evidentiary corpus, leveraging PICRUSt-based inference to reconstruct KO/EC and KEGG pathway representations that remained unexamined in the original publication. This distinction constitutes the crux of the manuscript’s contribution: while the antecedent study established empirically measured SCFA endpoints, the current re-analysis interrogates a complementary and hitherto unaddressed question—namely, which taxonomic compositions and predicted functional signatures covary with dietary fiber status, and might plausibly underlie the previously reported SCFA disparities. The present analysis is accordingly oriented toward discerning which taxonomic and computationally inferred functional patterns coincide with the source-study cohort designations, thereby identifying observations meriting subsequent empirical validation. Critically, the source investigation and this re-analysis diverge fundamentally in their evidentiary standing: a direct biochemical assay affords quantification of a measured endpoint under a precisely defined experimental paradigm, whereas 16S-derived inference merely approximates the representational prevalence of reference-associated functional capacities. The latter methodology, while capable of generating a testable mechanistic hypothesis, remains inherently incapable of establishing directionality, magnitude, or causal attribution.

The novelty in the present study is threefold. First, unlike Sanchez et al. (2020) [9], which focused on host immunology and biochemical SCFA validation, this study performs a systematic PICRUSt-based functional reconstruction of the same dataset, surfacing pathway-level hypotheses (carbohydrate, pyruvate, propanoate, butanoate metabolism) never reported originally. Second, compared with the prior SCFA-microbiome literature—which typically either measures SCFAs directly or infers function without a validated benchmark—this analysis uniquely pairs PICRUSt-predicted potential with a dataset carrying an established SCFA phenotype. Third, it explicitly leverages the NF-SCFA cohort as a dissociation design (fiber absence vs. exogenous SCFA repletion), testing whether functional signatures track fiber availability specifically rather than SCFA exposure alone.

The analysis of the present study also describes associations between dietary cohort and predicted functional representation rather than measured enzyme or metabolite changes [18,41,42]. Higher inferred representation of pathway-associated features cannot establish metabolite concentration, substrate bottlenecks, reaction direction, metabolic flux, enzyme activity, or SCFA output. The NF-SCFA profile did not reproduce the F-like pattern in the supplied data, but this observation is descriptive and cannot be interpreted as evidence of causal inhibition, host-mediated feedback, or failure of an exogenous SCFA treatment to restore a biochemical pathway. Because PICRUSt cannot measure transcripts, proteins, or metabolites directly, the analysis remains hypothesis-generating, proposing candidates for future biochemical validation.

SCFAs have established physiological roles in gut and immune biology, and the Sanchez et al. source study directly measured fecal and tissue SCFA concentrations in its experimental context [2,43]. The prior literature describes fiber fermentation as supporting communities that can be associated with SCFA-related functions, lower luminal pH, epithelial-barrier maintenance, and immune homeostasis [2,43,44,45,46]. Conversely, no-fiber diets can remove fermentable substrates and can be accompanied by changes in fiber-associated taxa, community diversity, and host–microbe interactions [47,48,49]. These mechanisms provide a rationale for examining whether the supplied 16S-derived profiles differ between F and NF cohorts. They do not convert inferred KO/EC or pathway representation into a measurement of SCFA concentration. In the present work, no direct SCFA assay, metabolomic characterization, flux analysis, transcriptomic measurement, proteomic measurement, or enzyme-activity assay was performed [9,45,50]. Accordingly, source-study observations and general biological mechanisms are discussed as context, while the current results are limited to taxonomic counts and PICRUSt-inferred functional potential. The source-study observations are consequently cited to clarify the biological question and the distinction from the present dataset, not to supply an outcome for the current analysis. A direct comparison of source-study SCFA concentrations with the inferred values in Tables S4–S7 would be inappropriate because the two evidence types have different units, denominators, and measurement procedures.

SCFAs are produced in many gut ecosystems through microbial metabolism of dietary and host-derived substrates, and prior studies have associated genera such as *Lactobacillus*, *Bifidobacterium*, *Akkermansia*, *Clostridium*, and *Faecalibacterium* with saccharolytic or SCFA-related functions [23,39,51,52]. In the supplied Table S3 profiles, several of these or related taxa were represented differently across the cohorts, including higher F read counts for selected *Clostridium*, *Lactobacillus*, and *Bifidobacterium* entries and higher NF *Ligilactobacillus* counts in several samples [53,54,55,56,57]. The comparison is not a census of all fiber-fermenting organisms, and a genus label does not specify the strain, gene complement, substrate preference, or reaction pathway that would be required to infer a particular product. Other candidate taxa, including *Prevotella*, *Coprococcus*, *Subdoligranulum*, and members of the *Lachnospiraceae* and *Ruminococcaceae*, may contribute to cross-feeding or carbohydrate transformation in some settings, but those possibilities were not resolved by the supplied genus-level table [58,59]. The present evidence therefore supports a cautious statement that taxonomic representations co-occurred with dietary cohort, not that a defined set of organisms generated a measured amount of acetate, propionate, or butyrate [54,60,61]. Direct culture, genome-resolved sequencing, transcript, protein, enzyme, and metabolite measurements would be required to evaluate those hypotheses [62]. In addition, apparent cohort differences may reflect multiple organisms sharing an annotation, changes in low-abundance taxa, compositional effects, or differences in the unmeasured diet constituents documented in the diet-comparison table. A taxon can be compatible with a pathway hypothesis without being necessary or sufficient for that pathway in the sampled community.

The NF-associated *Ligilactobacillus* pattern is compatible with a cohort difference in taxonomic representation, but 16S genus assignments do not resolve strain-level metabolic traits [63,64]. Earlier directional statements about *Lachnoclostridium* and *Romboutsia* were not retained because the supplied Table S3 entries do not support those directions consistently. Any proposed contribution of *Ligilactobacillus* or other NF-associated taxa to SCFA-related processes is therefore a hypothesis for targeted culture, genome, transcript, protein, enzyme, and metabolite measurements rather than a conclusion from this dataset [65]. This conservative interpretation preserves useful observation while avoiding a strain-level or metabolite-level conclusion that the available table cannot support.

The inferred KO/EC features mapped to butanoate, propanoate, pyruvate, carbohydrate, and amino-acid pathway groups provide candidate functional signatures in the processed profiles [30,39,54]. In the source literature, enzymes such as butyryl-CoA:acetate CoA-transferase, butyrate kinase, methylmalonyl-CoA mutase, propionate CoA-transferase, and related reactions have been discussed in relation to SCFA-associated pathways [30,39,54]. Their annotation in a predicted profile, however, does not demonstrate that the corresponding genes are expressed, that the proteins are present, or that the reactions proceed at a particular rate [30,39,54,66]. It also cannot distinguish alternative organisms, substrate pools, pathway direction, or cross-feeding relationships that may lead to the same functional annotation [39,40]. The values in Tables S4 and S5 should therefore be read as inferred representation that can prioritize experiments, not as direct enzyme quantities or as evidence of butyrate or propionate production. Targeted metatranscriptomics, metaproteomics, enzyme assays, stable-isotope tracing, flux analysis, and direct SCFA measurement would be needed to test the proposed functional interpretations [67,68]. The same annotation may occur in organisms with different genomes and regulatory states, and the tables do not provide the information needed to determine whether a feature is complete, expressed, or active in vivo. The inferred profile is thus best used to formulate experiments that measure the relevant genes, proteins, reactions, and products directly.

Under fiber-deprived conditions, the processed profiles contained differences in features annotated to ribose, galactose, fumarate, glutamine, glutamate, and related pathway groups [69,70]. These observations are compatible with altered representation of functions associated with alternative carbohydrate and amino-acid substrates, but they do not show that those substrates accumulated, that conversion stopped at a particular intermediate, or that bacteria were forced to use a specific route. Concentration, reaction rate, substrate turnover, and metabolic flux were not measured. The original biological interpretation that fiber deprivation can alter access to fermentable substrates remains a plausible hypothesis, but the present data support only higher predicted representation of pathway-associated features in selected NF profiles [48,71,72,73,74,75]. The same caution applies to Figure 6, Figure 7 and Figure S35: pathway annotations can organize a mechanistic hypothesis, whereas direct metabolite and enzyme measurements are required to establish pathway operation or SCFA output [3,43,48,70,76]. The profiles also do not distinguish whether an annotation belongs to a dominant organism or to several less abundant organisms, and they do not measure the environmental conditions that determine reaction direction. These limitations prevent a claim that NF profiles contained accumulated intermediates or that fiber deprivation redirected carbon or nitrogen flow.

Overall, the analysis of the present study is constrained by the compositional and small-sample nature of the underlying 16S rRNA data, reliance on PICRUSt-based functional inference rather than direct measurement, and the absence of a verifiable raw-data archive for independently reproducing the historical analysis pipeline. More specifically, the analysis of the present study is limited by a modest sample size of five animals per cohort (n = 15 total across F, NF, and NF-SCFA), which constrains statistical power, the precision of effect estimates, and the robustness and reproducibility of the observed microbial and functional patterns; at this scale, individual-animal variability can disproportionately influence read-count and pathway-representation summaries, and rare or low-abundance taxa are particularly susceptible to sampling noise. This limitation is compounded by the absence of direct SCFA, metabolomic, flux, transcriptomic, proteomic, and enzyme-activity measurements. Genus-level profiles cannot reliably resolve strain-level functional variation, horizontal gene transfer, gene expression, protein abundance, or the effects of substrate availability. The results also depend on the source study’s diet formulation and on the historical FLASH 1.2.7, QIIME 1.7.0, R 2.15.3/vegan, and PICRUSt 1.1.4 workflow documented in the manuscript; the supplied package does not include the raw exports, distance matrix, analysis scripts, or complete software environment needed to independently reproduce every historical calculation. The findings should therefore be treated as exploratory associations and predicted functional potential that require independent biochemical and molecular validation, ideally in an adequately powered, independently replicated animal cohort.

## 4. Materials and Methods

The source-study design comprised three cohorts of female wild-type C57BL/6 mice, with 15 animals in total and five animals assigned to each dietary condition. The F cohort received Teklad Mouse/Rat Sterilizable Diet LM-485 (Inotiv, Indianapolis, IN, USA), reported as containing approximately 18% crude fiber, whereas the NF cohort received Harlan TD.00129 AIN-93G No Cellulose (Inotiv, Indianapolis, IN, USA), reported as containing 0% fiber. The NF-SCFA cohort remained on the NF solid diet (CLEA Japan, Inc., Tsukuba, Japan) and received drinking-water supplementation containing the source-study SCFA cocktail at pH 7.4: 20.0 mg mL−1 tributyrin emulsion, 140 mM sodium butyrate, and 150 mM sodium propionate. According to the source-study description, the drinking-water supplementation in the NF-SCFA cohort was initiated at five weeks of age, and the fecal samples used for SCFA quantification and microbiota profiling were collected at 12 weeks of age, with the mice housed in a specific pathogen-free facility at the University of Texas Health Science Center at San Antonio; these procedural details are retained here only as inherited source-study context. The three-cohort arrangement was designed to separate the effect of dietary fiber itself (F versus NF) from the effect of exogenous short-chain fatty acids supplied without fiber (NF versus NF-SCFA). These product identities, fiber descriptions, and supplementation details are inherited source-study procedures and are summarized descriptively in the separate diet-comparison table. They should not be interpreted as proof that the solid diets differed only in fiber because the supplied source does not establish every energy, macronutrient, starch, sucrose, or micronutrient constituent. The present work is a computational re-analysis with no new animals, samples, sequencing, or wet-lab measurements.

Genomic DNA and 16S rRNA gene sequencing were performed in the source study, and the present work used the deposited and processed evidence package rather than generating new sequence data [9]. The public accessions are SAMN13226853–SAMN13226857 for F, SAMN13226858–SAMN13226862 for NF, and SAMN13226863–SAMN13226867 for NF-SCFA, as listed in Table S8. According to the source-study methods, DNA was extracted from fecal material with the Quick-DNA™ Fecal/Soil Microbe Microprep Kit (Zymo Research, Irvine, CA, USA); the hypervariable V3–V4 regions of the 16S rRNA gene were amplified with the barcoded primers bact341F and bact805R using Phusion high-fidelity DNA polymerase; amplicon libraries prepared with the Nextera XT Index Kit (Illumina, Inc., San Diego, CA, USA) were paired-end sequenced on an Illumina MiSeq platform. These laboratory details are reproduced here as inherited source-study context and were not re-executed in the present re-analysis. The source-study sample labels and accession assignments were retained without adding samples or changing the cohort structure. Because the raw sequence exports, laboratory records, and complete source-analysis archive were not supplied here, the present manuscript does not claim to reproduce library preparation, sequencing yield, quality-control thresholds, or provider-specific processing details beyond the historical workflow documented below.

The documented historical workflow was retained: FLASH 1.2.7 read merging [77]; QIIME 1.7.0 quality filtering and diversity analysis [78,79]; UCHIME/SILVA chimera handling; UPARSE 7.0.1090 OTU clustering; QIIME/Mothur taxonomic classification; R 2.15.3 with vegan for community statistics; and PICRUSt 1.1.4 for KEGG ortholog inference. In this pipeline, FLASH reconstructs full-length amplicons from overlapping paired-end reads; QIIME quality filtering removes low-quality and putatively erroneous sequences; UCHIME v11.0.667 [80], aligned against the SILVA release 144 reference database (http://www.arb-silva.de/ (accessed on 22 August 2026)), identifies and removes chimeric sequences; UPARSE clusters the surviving high-quality reads into operational taxonomic units (OTUs) [81]; and QIIME/Mothur classify representative OTU sequences against the SILVA taxonomy [82,83]. These stepwise descriptions are retained only as the documented historical workflow. The supplied package contains no verified source-analysis archive beyond the historical workflow, so no alternative software or raw-export provenance is asserted. The workflow description is retained as inherited source-study context; it does not imply that the missing raw exports or scripts are available for a local rerun.

Alpha-diversity metrics, including observed features, Shannon index, and Simpson index, were computed in the documented historical QIIME 1.7.0 workflow and visualized with R 2.15.3 [84,85]. The indices were used to summarize richness and evenness within the processed microbial profiles, not to estimate total bacterial biomass. Observed features counts the number of detected taxa, the Shannon index weights richness and evenness, and the Simpson index emphasizes dominance by the most abundant taxa, so the three indices together summarize within-sample diversity structure rather than bacterial load. The source description also refers to comparisons among dietary groupings and to the visualization of shared taxa, including Venn-style summaries, using the F, NF, and NF-SCFA labels.

Beta-diversity was represented using Bray–Curtis dissimilarities and PCoA. PCoA projects the sample-by-sample dissimilarity matrix into a reduced-dimensional ordination space, allowing the separation of the F, NF, and NF-SCFA profiles to be inspected visually; as with the other ordinations in this work, it is an exploratory display of community-level compositional structure. Differences in microbial community composition were assessed in the historical workflow with PERMANOVA implemented through the vegan package in R 2.15.3, using Bray–Curtis dissimilarities derived from genus-level profiles. PERMANOVA is a permutation-based multivariate procedure that tests whether the observed separation among cohort centroids in the dissimilarity space exceeds what would be expected by chance, making it appropriate for the sparse, non-Gaussian structure typical of microbial count data from small cohorts. For clarity, each cohort contained n = 5 animals and the complete dataset contained n = 15 animals. Within-group Bray–Curtis distances number (n) = 10 = C(5,2) per cohort, whereas between-group distances number (n) = 25 = 5 × 5 per cohort pair. These are pairwise-distance counts, not animal sample sizes. The ordination displays describe relationships among samples or cohort centroids in multivariate space; they do not themselves establish a biochemical mechanism.

PICRUSt 1.1.4 (http://picrust.github.io/picrust/ (accessed on 22 August 2026)) [86] was used in the documented historical workflow to infer KEGG ortholog and enzyme-associated profiles from 16S rRNA gene data. PICRUSt maps the processed genus-level composition onto reference-genome content to estimate which KEGG orthologs and enzyme-encoding features the community could carry, and the resulting profiles were then grouped into metabolic pathways; these are computational predictions conditioned on taxonomy and reference genomes, not direct measurements of microbial function.

Read-count ratios in this study describe how often a feature was queried within the filtered dataset, whereas the inferred profiles describe predicted functional capacity given the observed taxonomy; neither quantity should be read as a measured biological amount. These values must not be interpreted as independent bacterial-load or EC measurements, enzyme activity, metabolic flux, or metabolite concentration. Any relative representation shown in the figures is limited to the stated table denominator or within-feature display and does not convert the profiles into absolute measurements. The absence of the underlying raw exports and analysis archive further limits the precision with which normalization or historical filtering can be reconstructed.

## 5. Conclusions

This in silico re-analysis of 16S rRNA gene data from 15 female C57BL/6 mice offers a computational, hypothesis-generating perspective on how dietary fiber status relates to gut microbial community structure and predicted functional potential, complementing the direct biochemical measurements reported by the source study. The analysis found cohort-associated differences in taxonomic read counts and PICRUSt-predicted functional representations across the fiber-replete (F), no-fiber (NF), and no-fiber-plus-SCFA (NF-SCFA) cohorts: F showed the highest alpha-diversity and higher representation of fiber-associated taxa such as *Clostridium* and *Bifidobacterium*, NF showed elevated *Ligilactobacillus* and distinct pathway-associated features among 52 differentially represented KO/EC features (19 F-preferential, 33 NF-preferential) spanning carbohydrate, pyruvate, propanoate, and butanoate metabolism, and critically, NF-SCFA failed to reproduce the F-like pattern in either analysis, indicating that exogenous SCFA supplementation alone does not restore fiber-associated community structure or functional potential. These observations are exploratory, noncausal, and limited to a small source-study dataset; they do not demonstrate a change in bacterial biomass, enzyme abundance, metabolite concentration, metabolic flux, or SCFA output, and the results instead identify candidate taxa and pathway associations that warrant direct validation—via SCFA measurement, metabolomics, enzyme assays, transcript or protein measurements, and strain-resolved analysis—before any claim about pathway operation or host benefit can be made.

## Data Availability

Public 16S rRNA gene accessions SAMN13226853–SAMN13226867 are available through NCBI and are mapped to the three dietary cohorts in Table S1. The processed evidence used here is provided in Tables S3–S7, including taxonomic read-count profiles, PICRUSt-inferred KO/EC profiles, KEGG mappings, and predicted pathway profiles. These processed inferred profiles are not metabolomic data and do not provide absolute bacterial load, enzyme activity, metabolite concentration, or flux measurements. No claim is made that the historical analysis can be rerun without the missing raw exports, scripts, and software environment.

## Supplementary Materials

The following supporting information can be downloaded at https://www.mdpi.com/article/10.3390/ijms27177851/s1.

## Abbreviations

The following abbreviations are used in this manuscript:

AID	Activation-induced cytidine deaminase
ANOSIM	Analysis of Similarities
BH	Benjamini–Hochberg
DNA	Deoxyribonucleic acid
F	Fiber-replete cohort
HDAC	Histone deacetylase
KEGG	Kyoto Encyclopedia of Genes and Genomes
NCBI	National Center for Biotechnology Information
NF	No-fiber cohort
NF-SCFA	No-fiber cohort with exogenous short-chain fatty acid supplementation
OTU	Operational taxonomic unit
PCoA	Principal coordinate analysis
PERMANOVA	Permutational multivariate analysis of variance
PICRUSt	Phylogenetic Investigation of Communities by Reconstruction of Unobserved States
RNA	Ribonucleic acid
rRNA	Ribosomal RNA
SCFA	Short-chain fatty acid

## References

[1] Bui T.N., Paul A., Guleria S., O’Sullivan J.M., Toldi G. (2025). Short-chain fatty acids—A key link between the gut microbiome and T-lymphocytes in neonates?. Pediatr. Res..

[2] Liu M., Lu Y., Xue G., Han L., Jia H., Wang Z., Zhang J., Liu P., Yang C., Zhou Y. (2024). Role of short-chain fatty acids in host physiology. Anim. Models Exp. Med..

[3] Den Besten G., Van Eunen K., Groen A.K., Venema K., Reijngoud D.-J., Bakker B.M. (2013). The role of short-chain fatty acids in the interplay between diet, gut microbiota, and host energy metabolism. J. Lipid Res..

[4] Kong D., Schipper L., van Dijk G. (2021). Distinct effects of short chain fatty acids on host energy balance and fuel homeostasis with focus on route of administration and host species. Front. Neurosci..

[5] Luke A.M., Hegde M.N., Hegde N.N. (2025). Metabolic interplay of SCFA’s in the gut and oral microbiome: A link to health and disease. Front. Oral Health.

[6] Tan J., McKenzie C., Potamitis M., Thorburn A.N., Mackay C.R., Macia L. (2014). The role of short-chain fatty acids in health and disease. Adv. Immunol..

[7] Xiong R.-G., Zhou D.-D., Wu S.-X., Huang S.-Y., Saimaiti A., Yang Z.-J., Shang A., Zhao C.-N., Gan R.-Y., Li H.-B. (2022). Health benefits and side effects of short-chain fatty acids. Foods.

[8] Sankarganesh P., Bhunia A., Kumar A.G., Babu S., Gopukumar S., Lokesh E. (2025). Short-chain fatty acids (SCFAs) in gut health: Implications for drug metabolism and therapeutics. Med. Microecol..

[9] Sanchez H., Moroney J.B., Gan H., Shen T., Im J., Li T., Taylor J.R., Zan H., Casali P.G. (2020). B cell-intrinsic epigenetic modulation of antibody responses by dietary fiber-derived short-chain fatty acids. Nat. Commun..

[10] Colpaert R.M.W., Calore M. (2021). Epigenetics and microRNAs in cardiovascular diseases. Genomics.

[11] Sato F., Tsuchiya S., Meltzer S.J., Shimizu K. (2011). MicroRNAs and epigenetics. FEBS J..

[12] Shen T., Sanchez H., Zan H., Casali P.G. (2015). Genome-Wide Analysis Reveals Selective Modulation of microRNAs and mRNAs by Histone Deacetylase Inhibitor in B Cells Induced to Undergo Class-Switch DNA Recombination and Plasma Cell Differentiation. Front. Immunol..

[13] Nadeau S., Martins G.A. (2022). Conserved and Unique Functions of Blimp1 in Immune Cells. Front. Immunol..

[14] Zhu C., Chen G., Zhao Y., Gao X.-M., Wang J. (2018). Regulation of the Development and Function of B Cells by ZBTB Transcription Factors. Front. Immunol..

[15] Bönelt P., Wöhner M., Minnich M., Tagoh H., Fischer M., Jaritz M., Kavirayani A.M., Garimella M.G., Karlsson M.C.I., Busslinger M. (2018). Precocious expression of Blimp1 in B cells causes autoimmune disease with increased self-reactive plasma cells. EMBO J..

[16] Cronin P., Joyce S.A., O’Toole P.W., O’Connor E.M. (2021). Dietary fibre modulates the gut microbiota. Nutrients.

[17] Samami E., Starkweather A., Lyon D., Kelly D.L. (2025). Associations Between Dietary Fiber, the Gut Microbiota, and Health Outcomes in Breast Cancer Survivors: A Scoping Review. Clin. Nutr. Open Sci..

[18] Fu J., Zheng Y., Gao Y., Xu W. (2022). Dietary fiber intake and gut microbiota in human health. Microorganisms.

[19] Chettri D., Nad S., Konar U., Verma A.K. (2022). CAZyme from gut microbiome for efficient lignocellulose degradation and biofuel production. Front. Chem. Eng..

[20] Rowland I., Gibson G., Heinken A., Scott K., Swann J., Thiele I., Tuohy K. (2018). Gut microbiota functions: Metabolism of nutrients and other food components. Eur. J. Nutr..

[21] Glover J.S., Ticer T.D., Engevik M.A. (2022). Characterizing the mucin-degrading capacity of the human gut microbiota. Sci. Rep..

[22] Deleu S., Machiels K., Raes J., Verbeke K., Vermeire S. (2021). Short chain fatty acids and its producing organisms: An overlooked therapy for IBD?. eBioMedicine.

[23] Miller T.L., Wolin M.J. (1996). Pathways of acetate, propionate, and butyrate formation by the human fecal microbial flora. Appl. Environ. Microbiol..

[24] Frampton J., Murphy K.G., Frost G., Chambers E.S. (2020). Short-chain fatty acids as potential regulators of skeletal muscle metabolism and function. Nat. Metab..

[25] Martin-Gallausiaux C., Marinelli L., Blottière H.M., Larraufie P., Lapaque N. (2021). SCFA: Mechanisms and functional importance in the gut. Proc. Nutr. Soc..

[26] Ramos Meyers G., Samouda H., Bohn T. (2022). Short chain fatty acid metabolism in relation to gut microbiota and genetic variability. Nutrients.

[27] Charrier C., Duncan G.J., Reid M.D., Rucklidge G.J., Henderson D., Young P., Russell V.J., Aminov R.I., Flint H.J., Louis P. (2006). A novel class of CoA-transferase involved in short-chain fatty acid metabolism in butyrate-producing human colonic bacteria. Microbiology.

[28] Zhang B., Lingga C., Bowman C., Hackmann T.J. (2021). A new pathway for forming acetate and synthesizing ATP during fermentation in bacteria. Appl. Environ. Microbiol..

[29] Sato M., Yoshida Y., Nagano K., Hasegawa Y., Takebe J., Yoshimura F. (2016). Three CoA transferases involved in the production of short chain fatty acids in Porphyromonas gingivalis. Front. Microbiol..

[30] Van-Wehle T., Vital M. (2024). Investigating the response of the butyrate production potential to major fibers in dietary intervention studies. npj Biofilms Microbiomes.

[31] Hiseni P., Snipen L., Wilson R.C., Furu K., Hegge F.T., Rudi K. (2023). Prediction of high fecal propionate-to-butyrate ratios using 16S rRNA-based detection of bacterial groups with liquid array diagnostics. BioTechniques.

[32] Wang W., Dernst A., Martin B., Lorenzi L., Cadefau-Fabregat M., Phulphagar K., Wagener A., Budden C., Stair N., Wagner T. (2024). Butyrate and propionate are microbial danger signals that activate the NLRP3 inflammasome in human macrophages upon TLR stimulation. Cell Rep..

[33] Otten B.M., Sthijns M.M., Troost F.J. (2023). A combination of acetate, propionate, and butyrate increases glucose uptake in C2C12 myotubes. Nutrients.

[34] Almansour N., Choudhry K., Al-rashed F., Alqaderi H., Sindhu S., Al Mulla F., Ahmad R. (2025). Gut microbiota: A promising new target in immune tolerance. Front. Immunol..

[35] Pang A., Pu S., Pan Y., Huang N., Li D. (2025). Short-chain fatty acids from gut microbiota restore Th17/Treg balance in rheumatoid arthritis: Mechanisms and therapeutic potential. J. Transl. Autoimmun..

[36] Zhang D., Jian Y.-P., Zhang Y.-N., Li Y., Gu L.-T., Sun H.-H., Liu M.-D., Zhou H.-L., Wang Y.-S., Xu Z.-X. (2023). Short-chain fatty acids in diseases. Cell Commun. Signal..

[37] Cheng J., Hu H., Ju Y., Liu J., Wang M., Liu B., Zhang Y. (2024). Gut microbiota-derived short-chain fatty acids and depression: Deep insight into biological mechanisms and potential applications. Gen. Psychiatry.

[38] Caetano-Silva M.E., Rund L., Hutchinson N.T., Woods J.A., Steelman A.J., Johnson R.W. (2023). Inhibition of inflammatory microglia by dietary fiber and short-chain fatty acids. Sci. Rep..

[39] Kircher B., Woltemate S., Gutzki F.M., Schlüter D., Geffers R., Bähre H., Vital M. (2022). Predicting butyrate- and propionate-forming bacteria of gut microbiota from sequencing data. Gut Microbes.

[40] Reichardt N., Duncan S.H., Young P., Belenguer A., Leitch C.M., Scott K.P., Flint H.J., Louis P. (2014). Phylogenetic distribution of three pathways for propionate production within the human gut microbiota. ISME J..

[41] Karl J.P., Fagnant H.S., Radcliffe P.N., Wilson M., Karis A.J., Sayers B., Wijeyesekera A., Gibson G.R., Lieberman H.R., Giles G.E. (2025). Gut microbiota-targeted dietary supplementation with fermentable fibers and polyphenols prevents hypobaric hypoxia-induced increases in intestinal permeability. Am. J. Physiol.-Regul. Integr. Comp. Physiol..

[42] Abreu A.T.A.Y., Milke-García M., Argüello-Arévalo G., Calderón-De La Barca A., Carmona-Sánchez R., Consuelo-Sánchez A., Coss-Adame E., García-Cedillo M., Hernández-Rosiles V., Icaza-Chávez M. (2021). Dietary fiber and the microbiota: A narrative review by a group of experts from the Asociación Mexicana de Gastroenterología. Rev. Gastroenterol. Méx..

[43] Silva Y.P., Bernardi A., Frozza R.L. (2020). The role of short-chain fatty acids from gut microbiota in gut-brain communication. Front. Endocrinol..

[44] Gamage H.K.A.H., Tetu S.G., Chong R.W.W., Bucio-Noble D., Rosewarne C.P., Kautto L., Ball M.S., Molloy M.P., Packer N.H., Paulsen I.T. (2018). Fiber Supplements Derived From Sugarcane Stem, Wheat Dextrin and Psyllium Husk Have Different In Vitro Effects on the Human Gut Microbiota. Front. Microbiol..

[45] Zhang F., Fan D.-J., Huang J., Zuo T. (2022). The gut microbiome: Linking dietary fiber to inflammatory diseases. Med. Microecol..

[46] Baxter N.T., Schmidt A.W., Venkataraman A., Kim K.-S., Waldron C., Schmidt T.M. (2018). Dynamics of Human Gut Microbiota and Short-Chain Fatty Acids in Response to Dietary Interventions with Three Fermentable Fibers. mBio.

[47] Chuandong Z., Hu J., Li J., Wu Y., Wu C., Lai G., Shen H., Wu F., Tao C., Liu S. (2024). Distribution and roles of Ligilactobacillus murinus in hosts. Microbiol. Res..

[48] Morrison D.J., Preston T. (2016). Formation of short chain fatty acids by the gut microbiota and their impact on human metabolism. Gut Microbes.

[49] Yang Y., Song X., Wang G., Xia Y., Xiong Z., Ai L. (2024). Understanding Ligilactobacillus salivarius from probiotic properties to omics technology: A review. Foods.

[50] Mazhar M., Zhu Y., Qin L. (2023). The Interplay of Dietary Fibers and Intestinal Microbiota Affects Type 2 Diabetes by Generating Short-Chain Fatty Acids. Foods.

[51] Mukhopadhya I., Louis P. (2025). Gut microbiota-derived short-chain fatty acids and their role in human health and disease. Nat. Rev. Microbiol..

[52] Cummings J.H., Pomare E.W., Branch W.J., Naylor C.P., Macfarlane G.T. (1987). Short chain fatty acids in human large intestine, portal, hepatic and venous blood. Gut.

[53] Chen M., Fan B., Liu S., Imam K.M.S.U., Xie Y., Wen B., Xin F. (2020). The in vitro Effect of Fibers With Different Degrees of Polymerization on Human Gut Bacteria. Front. Microbiol..

[54] Singh V., Lee G., Son H., Koh H., Kim E.S., Unno T., Shin J.-H. (2023). Butyrate producers, “The Sentinel of Gut”: Their intestinal significance with and beyond butyrate, and prospective use as microbial therapeutics. Front. Microbiol..

[55] Fusco W., Lorenzo M.B., Cintoni M., Porcari S., Rinninella E., Kaitsas F., Lener E., Mele M.C., Gasbarrini A., Collado M.C. (2023). Short-Chain Fatty-Acid-Producing Bacteria: Key Components of the Human Gut Microbiota. Nutrients.

[56] Tsukuda N., Yahagi K., Hara T., Watanabe Y., Matsumoto H., Mori H., Higashi K., Tsuji H., Matsumoto S., Kurokawa K. (2021). Key bacterial taxa and metabolic pathways affecting gut short-chain fatty acid profiles in early life. ISME J..

[57] Chen T., Long W., Zhang C., Liu S., Zhao L., Hamaker B.R. (2017). Fiber-utilizing capacity varies in Prevotella- versus Bacteroides-dominated gut microbiota. Sci. Rep..

[58] Gerritsen J., Hornung B., Renckens B., van Hijum S.A., Dos Santos V.A.M., Rijkers G.T., Schaap P.J., de Vos W.M., Smidt H. (2017). Genomic and functional analysis of Romboutsia ilealis CRIBT reveals adaptation to the small intestine. PeerJ.

[59] Fan Z., Ke X., Jiang L., Zhang Z., Yi M., Liu Z., Cao J., Lu M., Wang M. (2024). Genomic and biochemical analysis reveals fermented product of a putative novel Romboutsia species involves the glycometabolism of tilapia. Aquaculture.

[60] Hodgkinson K., El Abbar F., Dobranowski P., Manoogian J., Butcher J., Figeys D., Mack D., Stintzi A. (2023). Butyrate’s role in human health and the current progress towards its clinical application to treat gastrointestinal disease. Clin. Nutr..

[61] Nie K., Ma K., Luo W., Shen Z., Yang Z., Xiao M., Tong T., Yang Y., Wang X. (2021). Roseburia intestinalis: A beneficial gut organism from the discoveries in genus and species. Front. Cell. Infect. Microbiol..

[62] Gerges P., Bangarusamy D.K., Bitar T., Alameddine A., Nemer G., Hleihel W. (2024). Turicibacter and Catenibacterium as potential biomarkers in autism spectrum disorders. Sci. Rep..

[63] Kopczyńska J., Kowalczyk M. (2024). The potential of short-chain fatty acid epigenetic regulation in chronic low-grade inflammation and obesity. Front. Immunol..

[64] Xu L., Qiu B., Ba F., Zhang S., Han S., Chen H., Wu Y., Gao W., Xie S., Chen Y. (2024). Synergistic effects of Ligilactobacillus salivarius Li01 and psyllium husk prevent mice from developing loperamide-induced constipation. Food Funct..

[65] Jensen N., Maldonado-Gomez M., Krishnakumar N., Weng C.-Y., Castillo J., Razi D., Kalanetra K., German J.B., Lebrilla C.B., Mills D.A. (2024). Dietary fiber monosaccharide content alters gut microbiome composition and fermentation. Appl. Environ. Microbiol..

[66] Frolova M.S., Suvorova I.A., Iablokov S.N., Petrov S.N., Rodionov D.A. (2022). Genomic reconstruction of short-chain fatty acid production by the human gut microbiota. Front. Mol. Biosci..

[67] Hesslinger C., Fairhurst S.A., Sawers G. (1998). Novel keto acid formate-lyase and propionate kinase enzymes are components of an anaerobic pathway in Escherichia coli that degrades L-threonine to propionate. Mol. Microbiol..

[68] Yoshida Y., Sato M., Nonaka T., Hasegawa Y., Kezuka Y. (2019). Characterization of the phosphotransacetylase-acetate kinase pathway for ATP production in Porphyromonas gingivalis. J. Oral Microbiol..

[69] Meiners F., Fuellen G., Barrantes I. (2025). Gut microbiome-mediated health effects of fiber and polyphenol-rich dietary interventions. Front. Nutr..

[70] Desai M.S., Seekatz A.M., Koropatkin N.M., Kamada N., Hickey C.A., Wolter M., Pudlo N.A., Kitamoto S., Terrapon N., Muller A. (2016). A dietary fiber-deprived gut microbiota degrades the colonic mucus barrier and enhances pathogen susceptibility. Cell.

[71] Basen M., Kurrer S.E. (2021). A close look at pentose metabolism of gut bacteria. FEBS J..

[72] Mhd Omar N.A., Dicksved J., Kruger J., Zamaratskaia G., Michaëlsson K., Wolk A., Frank J., Landberg R. (2022). Effect of a diet rich in galactose or fructose, with or without fructooligosaccharides, on gut microbiota composition in rats. Front. Nutr..

[73] Xu K., Ren Y., Zhao S., Feng J., Wu Q., Gong X., Chen J., Xie P. (2024). Oral D-ribose causes depressive-like behavior by altering glycerophospholipid metabolism via the gut-brain axis. Commun. Biol..

[74] Karri V., Hirschi K.D. (2020). Gut Bacteria have a novel sweet tooth: Ribose sensing and scavenging from fiber. Gut Microbes.

[75] Wang Z., Kang S., Wu Z., Liu X., Zhang X., Wu Y., Wen Y., Zhou X., Zhang G., Wang J. (2025). Muribaculum intestinale restricts Salmonella Typhimurium colonization by converting succinate to propionate. ISME J..

[76] Eliot G., Shronts E.P. (1992). Intestinal fuels: Glutamine, short-chain fatty acids, and dietary fiber. J. Am. Diet. Assoc..

[77] Magoč T., Salzberg S.L. (2011). FLASH: Fast length adjustment of short reads to improve genome assemblies. Bioinformatics.

[78] Caporaso J.G., Kuczynski J., Stombaugh J., Bittinger K., Bushman F.D., Costello E.K., Fierer N., Peña A.G., Goodrich J.K., Gordon J.I. (2010). QIIME allows analysis of high-throughput community sequencing data. Nat. Methods.

[79] Bokulich N.A., Subramanian S., Faith J.J., Gevers D., Gordon J.I., Knight R., Mills D.A., Caporaso J.G. (2013). Quality-filtering vastly improves diversity estimates from Illumina amplicon sequencing. Nat. Methods.

[80] Edgar R.C., Haas B.J., Clemente J.C., Quince C., Knight R. (2011). UCHIME improves sensitivity and speed of chimera detection. Bioinformatics.

[81] Edgar R.C. (2013). UPARSE: Highly accurate OTU sequences from microbial amplicon reads. Nat. Methods.

[82] Altschul S.F., Gish W., Miller W., Myers E.W., Lipman D.J. (1990). Basic local alignment search tool. J. Mol. Biol..

[83] Quast C., Pruesse E., Yilmaz P., Gerken J., Schweer T., Yarza P., Peplies J., Glöckner F.O. (2012). The SILVA ribosomal RNA gene database project: Improved data processing and web-based tools. Nucleic Acids Res..

[84] R Core Team (2013). R: A Language and Environment for Statistical Computing.

[85] Li B., Zhang X., Guo F., Wu W., Zhang T. (2013). Characterization of tetracycline resistant bacterial community in saline activated sludge using batch stress incubation with high-throughput sequencing analysis. Water Res..

[86] Langille M.G., Zaneveld J., Caporaso J.G., McDonald D., Knights D., Reyes J.A., Clemente J.C., Burkepile D.E., Thurber R.L.V., Knight R. (2013). Predictive functional profiling of microbial communities using 16S rRNA marker gene sequences. Nat. Biotechnol..

