# Supplementary material for: Dietary Fiber-Associated Differences in Gut Microbial Community Composition and Predicted Short-Chain Fatty Acid-Related Functional Potential: An In Silico Re-Analysis of 16S rRNA Data
Source: Int J Mol Sci. 2026 Sep 2;27(17):7851. doi: 10.3390/ijms27177851 (PMC13566324; doi:10.3390/ijms27177851)
Supplement: Supplementary file 1 [file ijms-27-07851-s001.zip › ijms-4484762-supplementary figures.pdf]

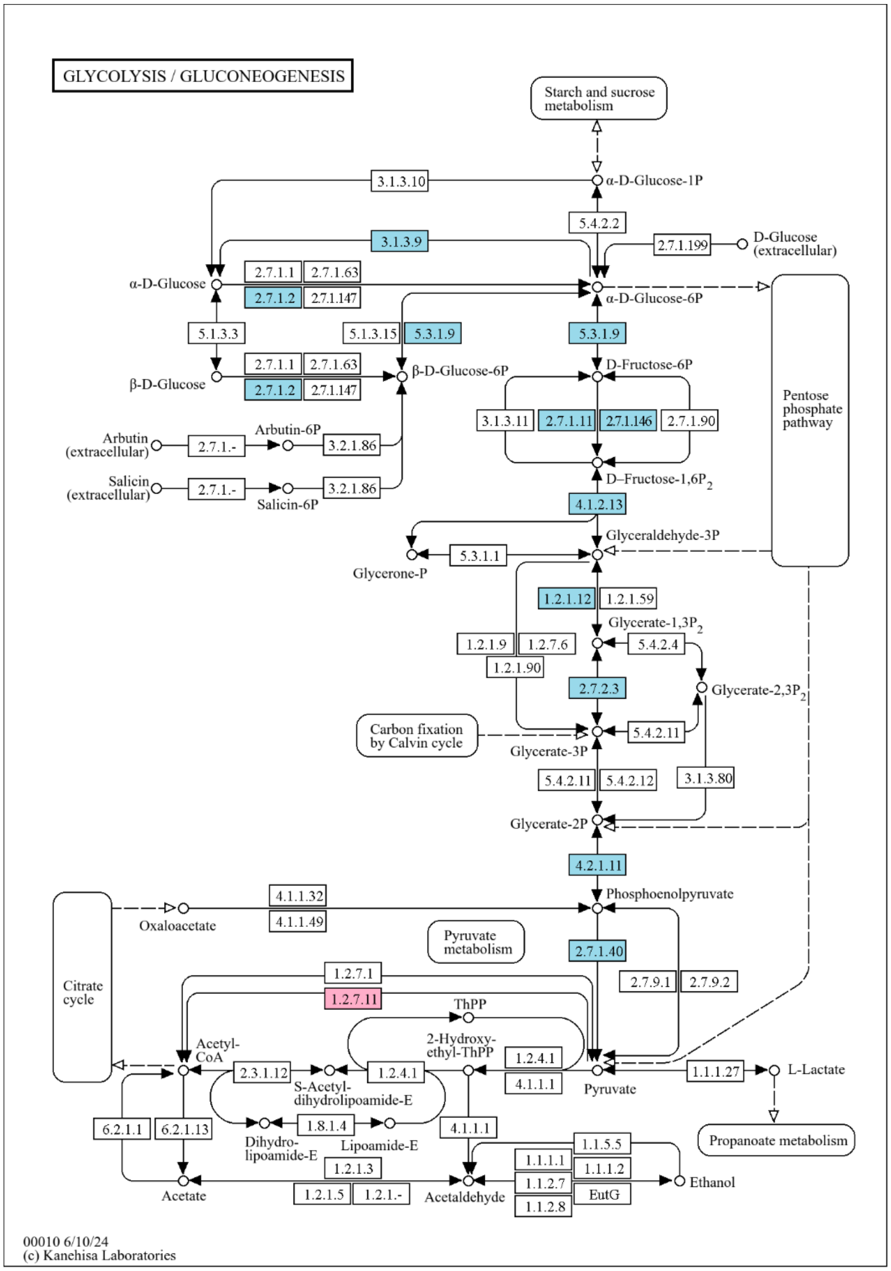

Figure S1. KEGG pathway "Glycolysis/ Gluconeogenesis" (map00010), delineating enzyme(s) and/or enzymatic constituents manifesting high abundance (demarcated by light blue boxes) within the intestinal microbial consortium of female C57BL/6 mice subjected to a fiber-devoid (NF) nutritional paradigm, juxtaposed against experimental cohorts administered either fiber-replete (F) or fiber-devoid diets augmented with exogenous short-chain fatty acids (NF-SCFA). These biocatalytic entities orchestrate the biosynthetic transformation of alternative non-polysaccharide substrates—including proteinaceous amino acid moieties and monosaccharide metabolites synthesized by the gut microbiome, which subsequently undergo anaerobic fermentative catabolism, culminating in the generation of short-chain fatty acids. EC 2.7.1.2 = glucokinase, EC 5.3.1.9 = glucose-6-phosphate isomerase, EC 5.3.1.1 = triosephosphate isomerase, EC 4.2.1.11 = enolase, EC 2.7.2.3 = phosphoglycerate kinase, EC 1.2.1.12 = glyceraldehyde-3-phosphate dehydrogenase, EC 2.7.1.40 = pyruvate kinase, EC 4.1.2.13 = fructose-bisphosphate aldolase, class I & II, EC 2.7.1.146 = ADP-dependent phosphofructokinase/glucokinase, EC 1.2.7.11 = 2-oxoglutarate/2-oxoacid ferredoxin oxidoreductase subunit alpha, EC 2.7.1.11 = 6-phosphofructokinase. Enzymes exhibiting pronounced enrichment in fiber-replete (F) dietary cohort versus fiber-devoid (NF) or exogenous SCFA-supplemented (NF-SCFA) groups are demarcated by pink boxes, whereas enzymes demonstrating pronounced enrichment in fiber-devoid (NF) cohort relative to fiber-replete (F) or SCFA-supplemented (NF-SCFA) groups are demarcated by light blue boxes.

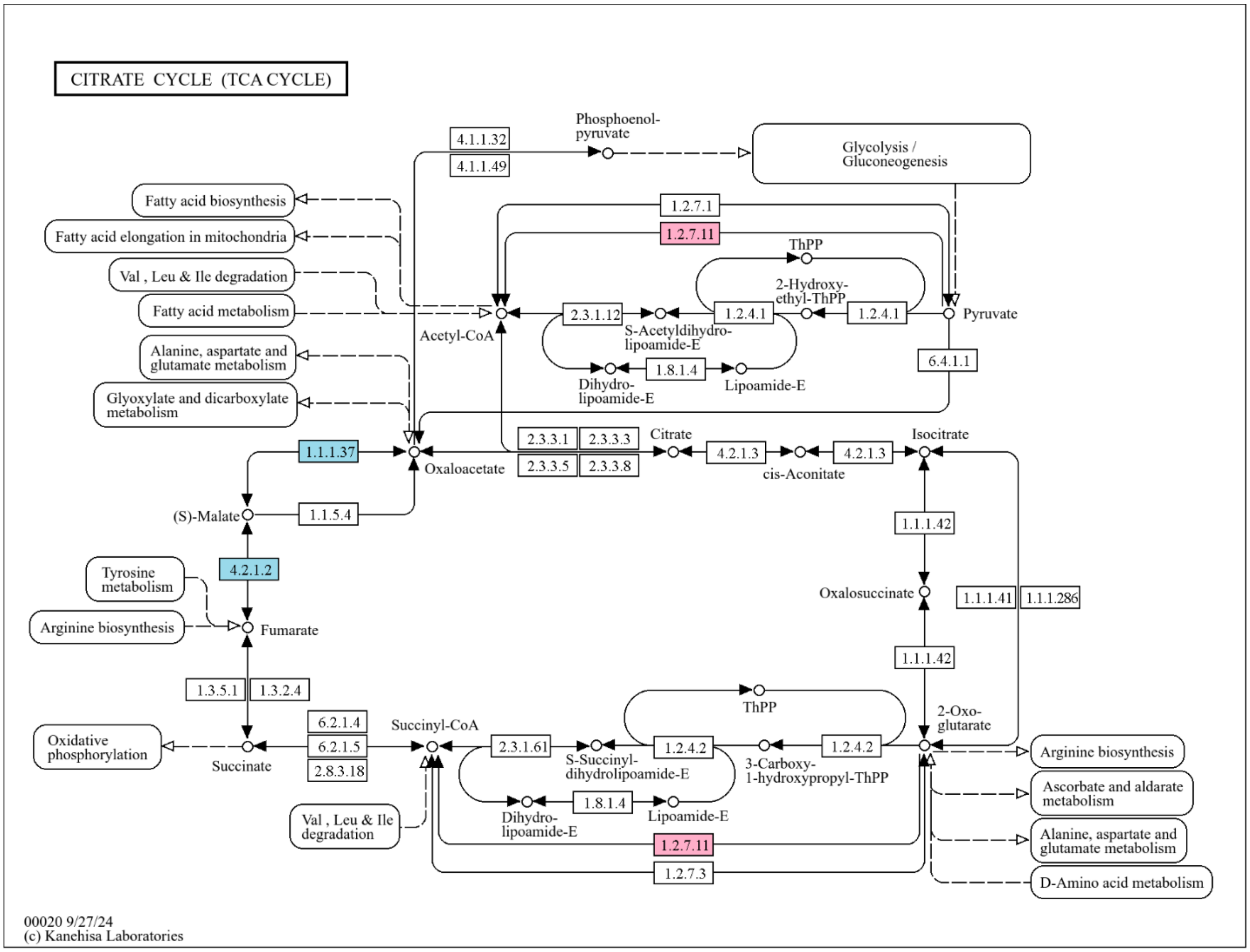

Figure S2. KEGG pathway "Citrate cycle" (TCA cycle, map00020), delineating enzyme(s) and/or enzymatic constituents manifesting high abundance (demarcated by light blue boxes) within the intestinal microbial consortium of female C57BL/6 mice subjected to a fiber-devoid (NF) nutritional paradigm, juxtaposed against experimental cohorts administered either fiber-replete (F) or fiber-devoid diets augmented with exogenous short-chain fatty acids (NF-SCFA). These biocatalytic entities orchestrate the biosynthetic transformation of alternative non-polysaccharide substrates—including proteinaceous amino acid moieties and monosaccharide metabolites synthesized by the gut microbiome, which subsequently undergo anaerobic fermentative catabolism, culminating in the generation of short-chain fatty acids. EC 1.2.7.11 = 2-oxoglutarate/2-oxoacid ferredoxin oxidoreductase subunit alpha, EC 4.2.1.2 = fumarate hydratase, class II, EC 1.1.1.37 = malate dehydrogenase. Enzymes exhibiting pronounced enrichment in fiber-replete (F) dietary cohort versus fiber-devoid (NF) or exogenous SCFA-supplemented (NF-SCFA) groups are demarcated by pink boxes, whereas enzymes demonstrating pronounced enrichment in fiber-devoid (NF) cohort relative to fiber-replete (F) or SCFA-supplemented (NF-SCFA) groups are demarcated by light blue boxes.

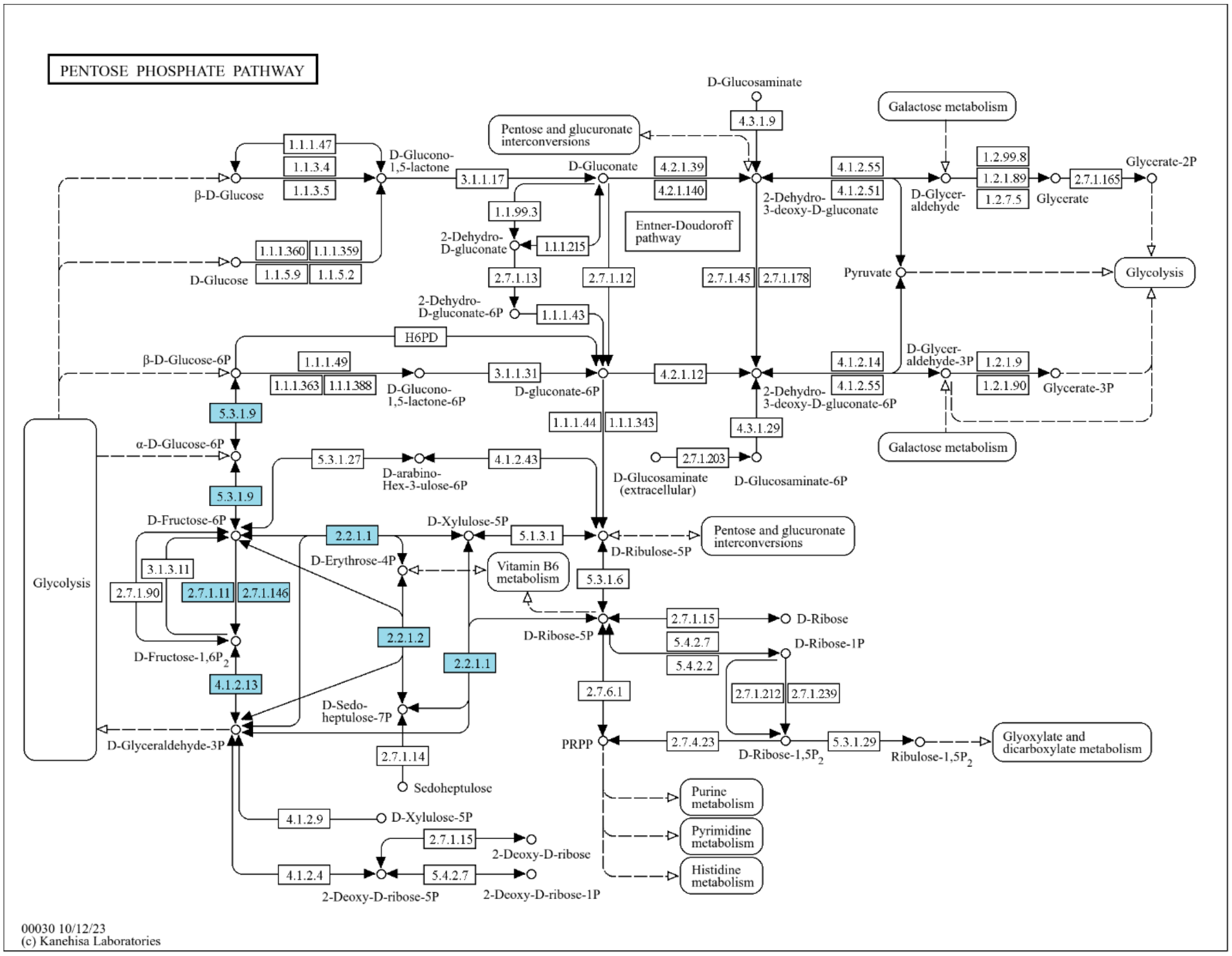

Figure S3. KEGG pathway "Pentose phosphate" (map00030), delineating enzyme(s) and/or enzymatic constituents manifesting high abundance (demarcated by light blue boxes) within the intestinal microbial consortium of female C57BL/6 mice subjected to a fiber-devoid (NF) nutritional paradigm, juxtaposed against experimental cohorts administered either fiber-replete (F) or fiber-devoid diets augmented with exogenous short-chain fatty acids (NF-SCFA). These biocatalytic entities orchestrate the biosynthetic transformation of alternative non-polysaccharide substrates—including proteinaceous amino acid moieties and monosaccharide metabolites synthesized by the gut microbiome, which subsequently undergo anaerobic fermentative catabolism, culminating in the generation of short-chain fatty acids. EC 5.3.1.9 = glucose-6-phosphate isomerase, EC 2.2.1.1 = transketolase, EC 2.2.1.2 = transaldolase, EC 4.1.2.13 = fructose-bisphosphate aldolase, class I & II, EC 2.7.1.11 = 6-phosphofructokinase. Enzymes exhibiting pronounced enrichment in fiber-replete (F) dietary cohort versus fiber-devoid (NF) or exogenous SCFA-supplemented (NF-SCFA) groups are demarcated by pink boxes, whereas enzymes demonstrating pronounced enrichment in fiber-devoid (NF) cohort relative to fiber-replete (F) or SCFA-supplemented (NF-SCFA) groups are demarcated by light blue boxes.

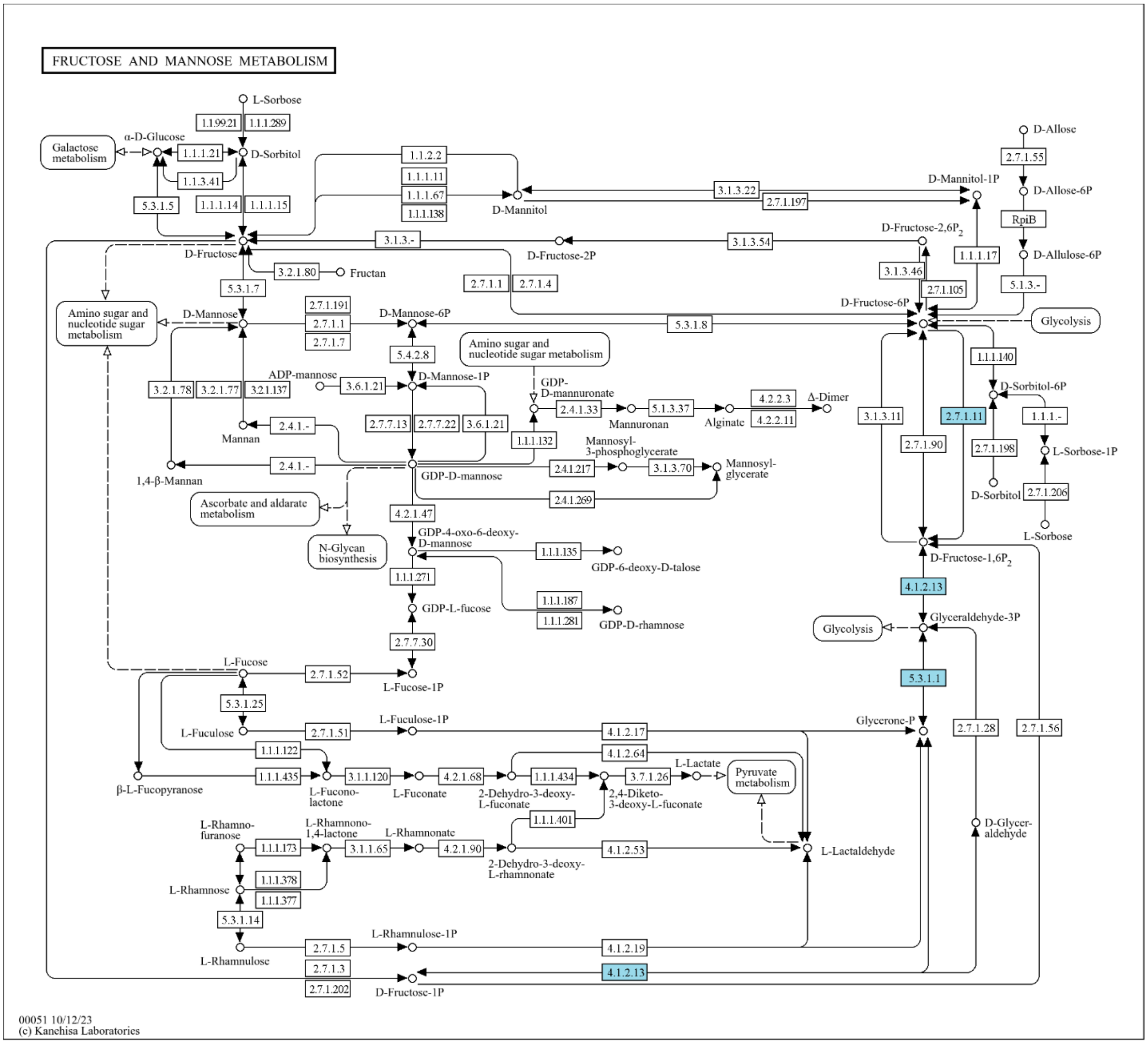

Figure S4. KEGG pathway "Fructose and mannose metabolism" (map00051), delineating enzyme(s) and/or enzymatic constituents manifesting high abundance (demarcated by light blue boxes) within the intestinal microbial consortium of female C57BL/6 mice subjected to a fiber-devoid (NF) nutritional paradigm, juxtaposed against experimental cohorts administered either fiber-replete (F) or fiber-devoid diets augmented with exogenous short-chain fatty acids (NF-SCFA). These biocatalytic entities orchestrate the biosynthetic transformation of alternative non-polysaccharide substrates—including proteinaceous amino acid moieties and monosaccharide metabolites synthesized by the gut microbiome, which subsequently undergo anaerobic fermentative catabolism, culminating in the generation of short-chain fatty acids. EC 5.3.1.1 = triosephosphate isomerase, EC 4.1.2.13 = fructose-bisphosphate aldolase, class I & II, EC 2.7.1.11 = 6-phosphofructokinase. Enzymes exhibiting pronounced enrichment in fiber-replete (F) dietary cohort versus fiber-devoid (NF) or exogenous SCFA-supplemented (NF-SCFA) groups are demarcated by pink boxes, whereas enzymes demonstrating pronounced enrichment in fiber-devoid (NF) cohort relative to fiber-replete (F) or SCFA-supplemented (NF-SCFA) groups are demarcated by light blue boxes.

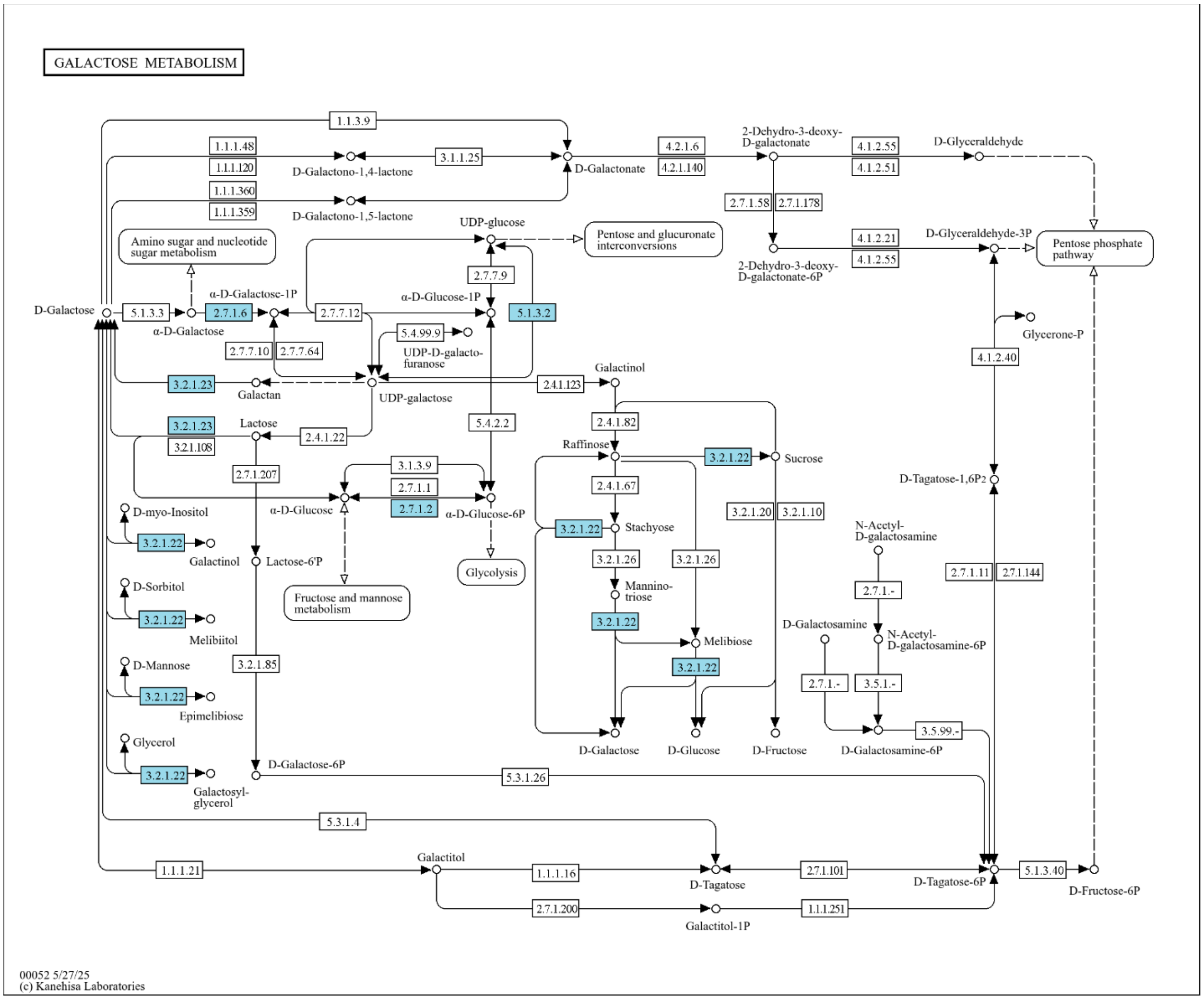

Figure S5. KEGG pathway "Galactose metabolism" (map00052), delineating enzyme(s) and/or enzymatic constituents manifesting high abundance (demarcated by light blue boxes) within the gut microbial consortium of female C57BL/6 mice subjected to a fiber-devoid (NF) nutritional paradigm, juxtaposed against experimental cohorts administered either fiber-replete (F) or fiber-devoid diets augmented with exogenous short-chain fatty acids (NF-SCFA). These biocatalytic entities orchestrate the biosynthetic transformation of alternative non-polysaccharide substrates—including proteinaceous amino acid moieties and monosaccharide metabolites synthesized by the gut microbiome, which subsequently undergo anaerobic fermentative catabolism, culminating in the generation of short-chain fatty acids. EC 2.7.1.2 = glucokinase, EC 3.2.1.23 = beta-galactosidase, EC 3.2.1.22 = alpha-galactosidase, EC 5.1.3.2 = UDP-glucose 4-epimerase, EC 2.7.1.6 = galactokinase. Enzymes exhibiting pronounced enrichment in fiber-replete (F) dietary cohort versus fiber-devoid (NF) or exogenous SCFA-supplemented (NF-SCFA) groups are demarcated by pink boxes, whereas enzymes demonstrating pronounced enrichment in fiber-devoid (NF) cohort relative to fiber-replete (F) or SCFA-supplemented (NF-SCFA) groups are demarcated by light blue boxes.

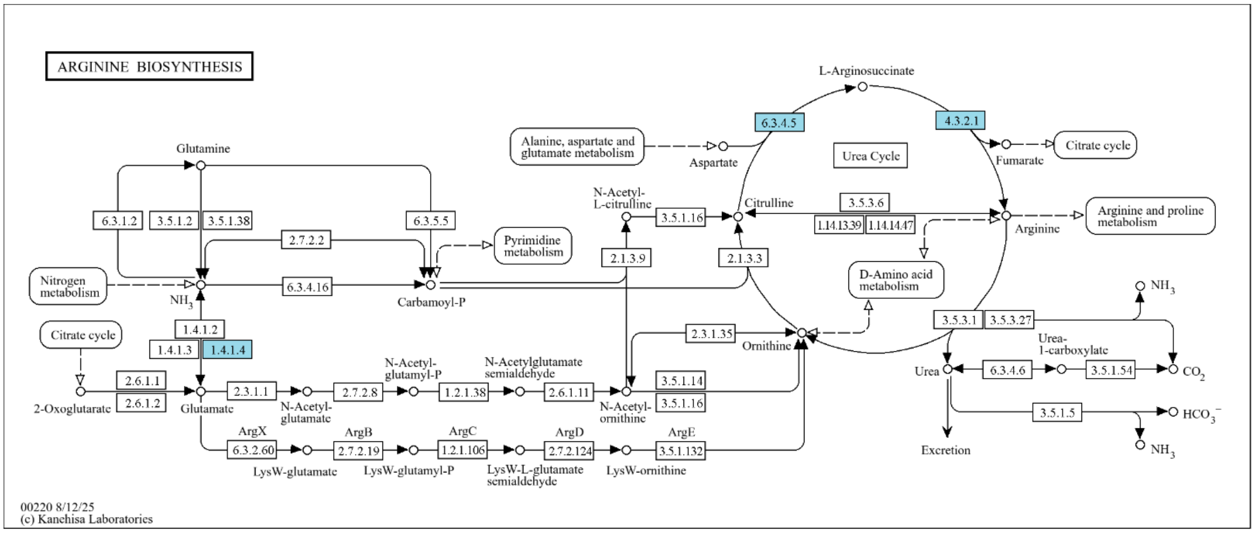

Figure S6. KEGG pathway "Arginine biosynthesis" (map00220), delineating enzyme(s) and/or enzymatic constituents manifesting high abundance (demarcated by light blue boxes) within the intestinal microbial consortium of female C57BL/6 mice subjected to a fiber-devoid (NF) nutritional paradigm, juxtaposed against experimental cohorts administered either fiber-replete (F) or fiber-devoid diets augmented with exogenous short-chain fatty acids (NF-SCFA). These biocatalytic entities orchestrate the biosynthetic transformation of alternative non-polysaccharide substrates—including proteinaceous amino acid moieties and monosaccharide metabolites synthesized by the gut microbiome, which subsequently undergo anaerobic fermentative catabolism, culminating in the generation of short-chain fatty acids. EC 4.3.2.1 = argininosuccinate lyase, EC 1.4.1.4 = glutamate dehydrogenase (NADP+), EC 6.3.4.5 = argininosuccinate synthase. Enzymes exhibiting pronounced enrichment in fiber-replete (F) dietary cohort versus fiber-devoid (NF) or exogenous SCFA-supplemented (NF-SCFA) groups are demarcated by pink boxes, whereas enzymes demonstrating pronounced enrichment in fiber-devoid (NF) cohort relative to fiber-replete (F) or SCFA-supplemented (NF-SCFA) groups are demarcated by light blue boxes.

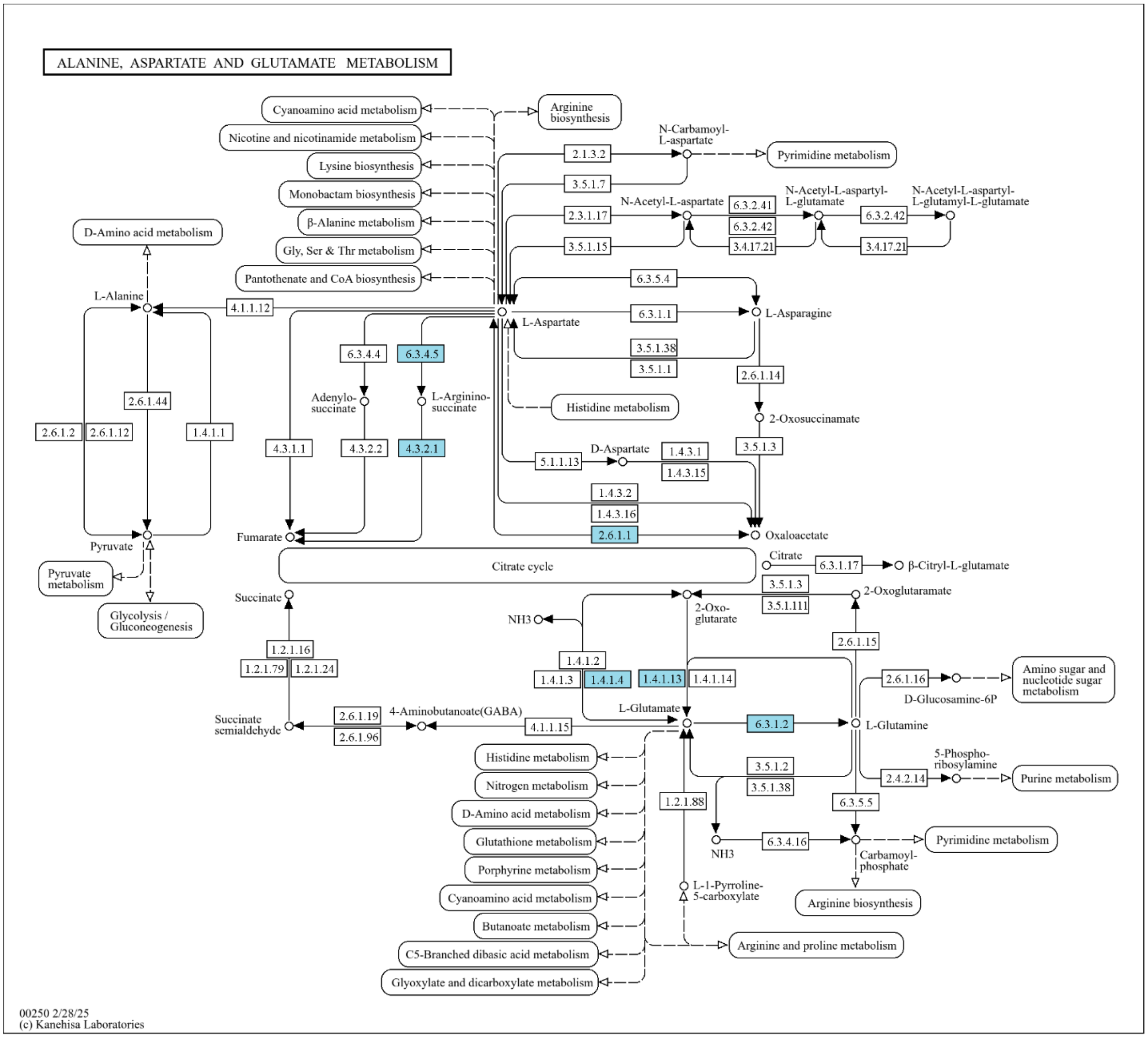

Figure S7. KEGG pathway "Alanine, aspartate and glutamate metabolism" (map00250), delineating enzyme(s) and/or enzymatic constituents manifesting high abundance (demarcated by light blue boxes) within the intestinal microbial consortium of female C57BL/6 mice subjected to a fiber-devoid (NF) nutritional paradigm, juxtaposed against experimental cohorts administered either fiber-replete (F) or fiber-devoid diets augmented with exogenous short-chain fatty acids (NF-SCFA). These biocatalytic entities orchestrate the biosynthetic transformation of alternative non-polysaccharide substrates—including proteinaceous amino acid moieties and monosaccharide metabolites synthesized by the gut microbiome, which subsequently undergo anaerobic fermentative catabolism, culminating in the generation of short-chain fatty acids. EC 1.4.1.4 = glutamate dehydrogenase (NADP+), EC 1.4.1.13 = glutamate synthase (NADPH/NADH) small chain, EC 6.3.1.2 = glutamine synthetase, EC 6.3.4.5 = argininosuccinate synthase, EC 4.3.2.1 = argininosuccinate lyase, EC 2.6.1.1 = aspartate aminotransferase. Enzymes exhibiting pronounced enrichment in fiber-replete (F) dietary cohort versus fiber-devoid (NF) or exogenous SCFA-supplemented (NF-SCFA) groups are demarcated by pink boxes, whereas enzymes demonstrating pronounced enrichment in fiber-devoid (NF) cohort relative to fiber-replete (F) or SCFA-supplemented (NF-SCFA) groups are demarcated by light blue boxes.

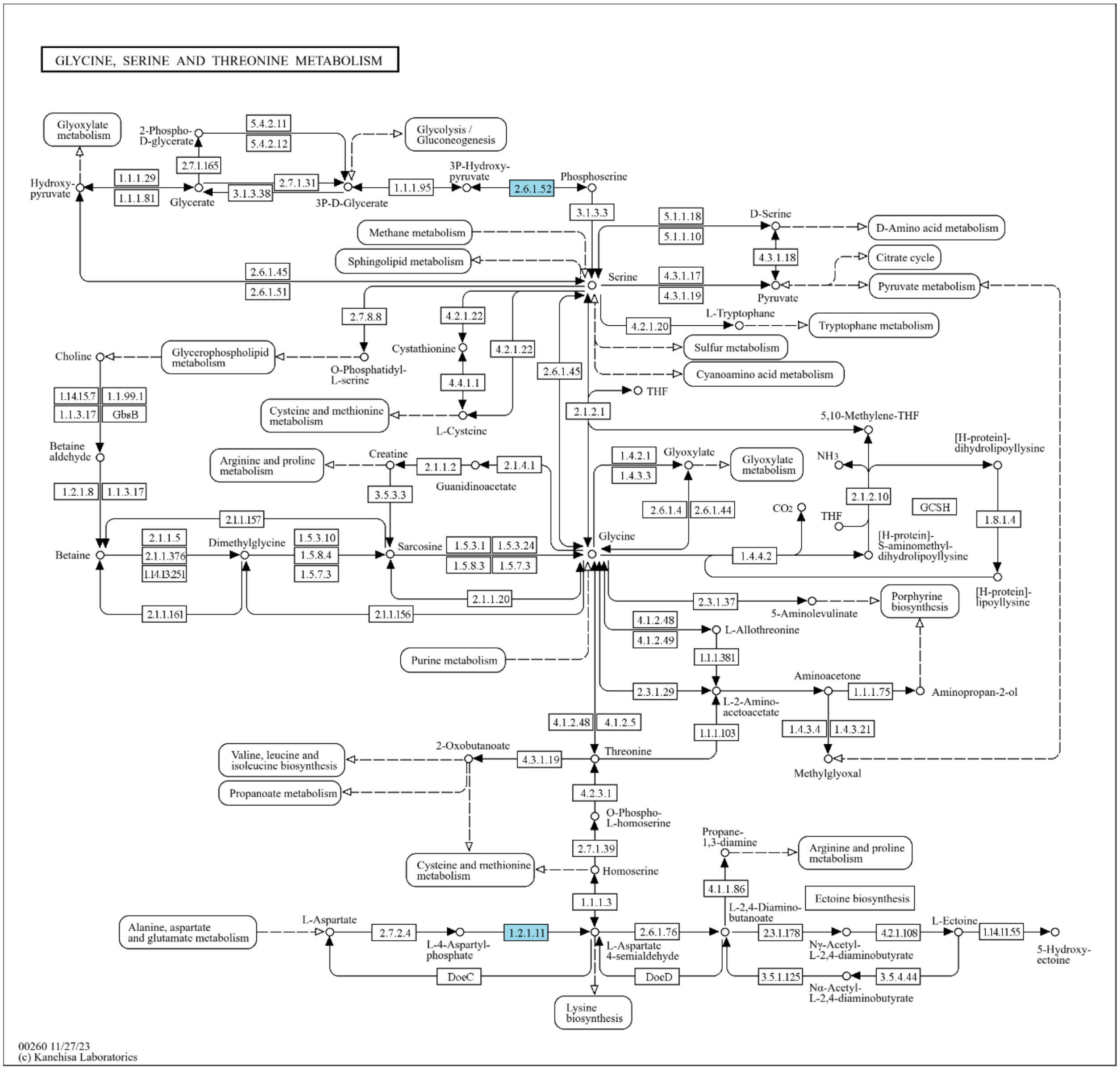

Figure S8. KEGG pathway "Glycine, serine and threonine metabolism" (map00260), delineating enzyme(s) and/or enzymatic constituents manifesting high abundance (demarcated by light blue boxes) within the intestinal microbial consortium of female C57BL/6 mice subjected to a fiber-devoid (NF) nutritional paradigm, juxtaposed against experimental cohorts administered either fiber-replete (F) or fiber-devoid diets augmented with exogenous short-chain fatty acids (NF-SCFA). These biocatalytic entities orchestrate the biosynthetic transformation of alternative non-polysaccharide substrates—including proteinaceous amino acid moieties and monosaccharide metabolites synthesized by the gut microbiome, which subsequently undergo anaerobic fermentative catabolism, culminating in the generation of short-chain fatty acids. EC 1.2.1.11 = aspartate-semialdehyde dehydrogenase, EC 2.6.1.52 = phosphoserine aminotransferase. Enzymes exhibiting pronounced enrichment in fiber-replete (F) dietary cohort versus fiber-devoid (NF) or exogenous SCFA-supplemented (NF-SCFA) groups are demarcated by pink boxes, whereas enzymes demonstrating pronounced enrichment in fiber-devoid (NF) cohort relative to fiber-replete (F) or SCFA-supplemented (NF-SCFA) groups are demarcated by light blue boxes.

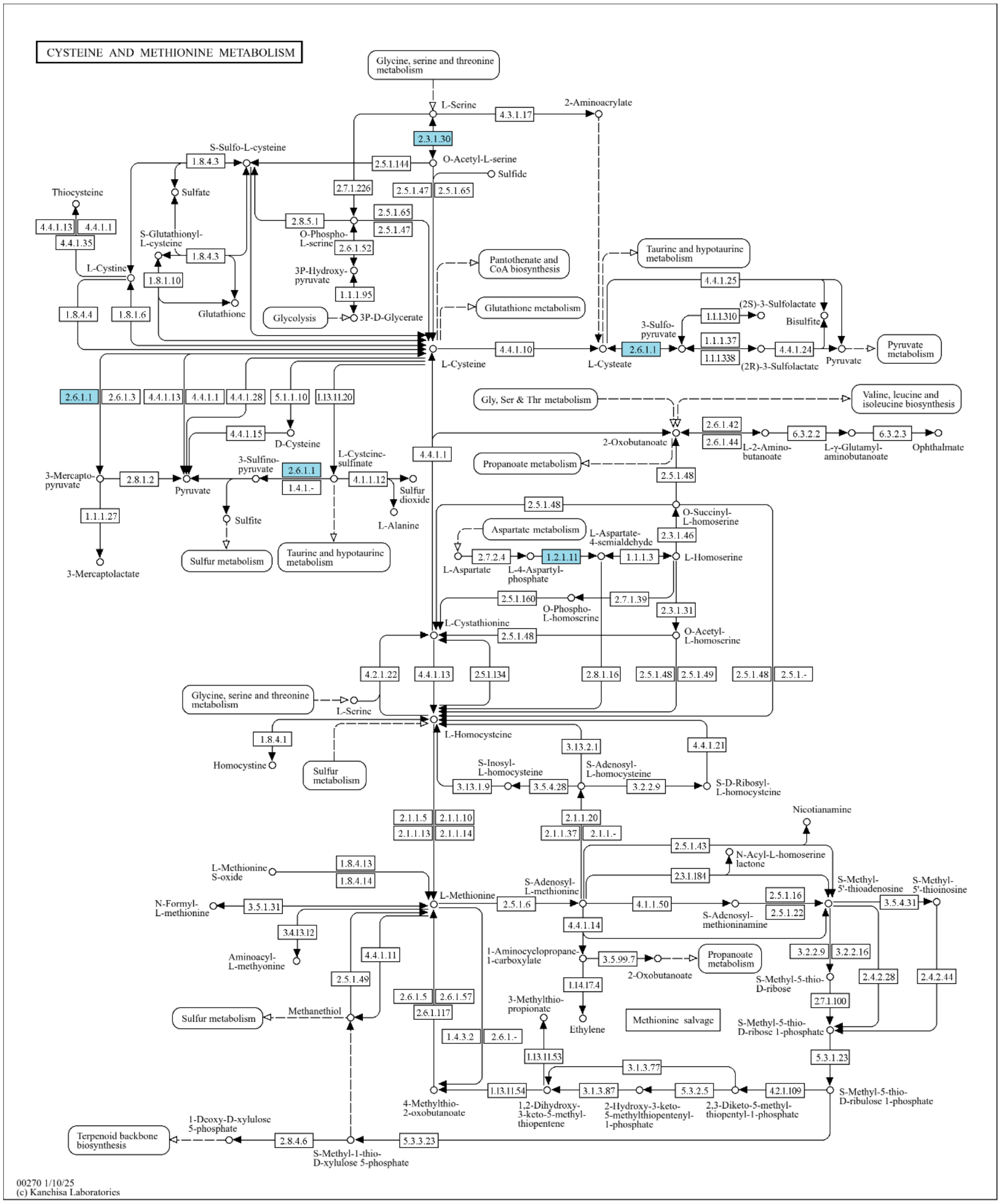

Figure S9. KEGG pathway “Cysteine and methionine metabolism” (map00270), delineating enzyme(s) and/or enzymatic constituents manifesting high abundance (demarcated by light blue boxes) within the intestinal microbial consortium of female C57BL/6 mice subjected to a fiber-devoid (NF) nutritional paradigm, juxtaposed against experimental cohorts administered either fiber-replete (F) or fiber-devoid diets augmented with exogenous short-chain fatty acids (NF-SCFA). These biocatalytic entities orchestrate the biosynthetic transformation of alternative non-polysaccharide substrates—including proteinaceous amino acid moieties and monosaccharide metabolites synthesized by the gut microbiome, which subsequently undergo anaerobic fermentative catabolism, culminating in the generation of short-chain fatty acids. EC 1.2.1.11 = aspartate-semialdehyde dehydrogenase, EC 2.3.1.30 = serine acetyltransferase, EC 2.6.1.1 = aspartate aminotransferase. Enzymes exhibiting pronounced enrichment in fiber-replete (F) dietary cohort versus fiber-devoid (NF) or exogenous SCFA-supplemented (NF-SCFA) groups are demarcated by pink boxes, whereas enzymes demonstrating pronounced enrichment in fiber-devoid (NF) cohort relative to fiber-replete (F) or SCFA-supplemented (NF-SCFA) groups are demarcated by light blue boxes.

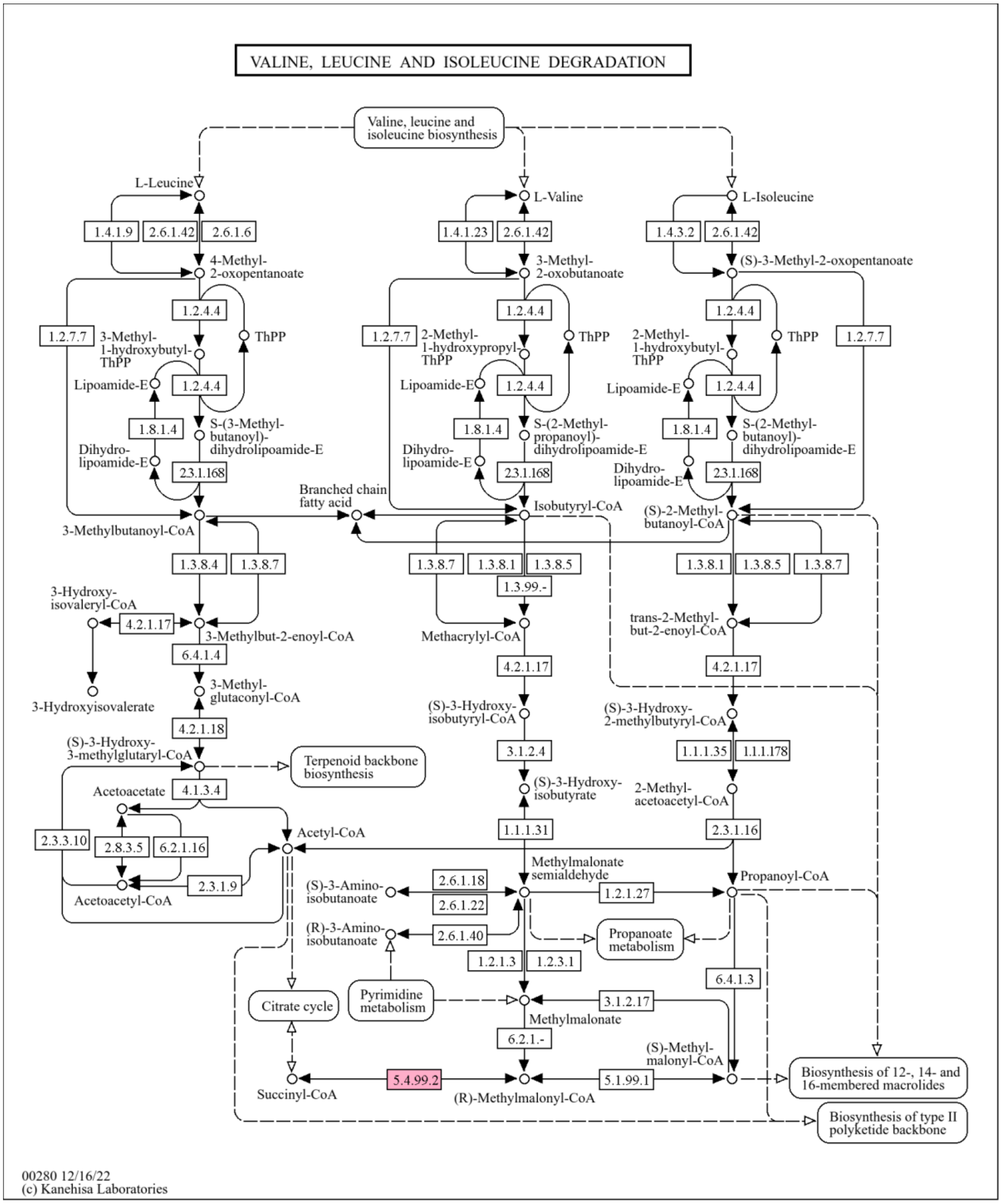

Figure S10. KEGG pathway "Valine, leucine and isoleucine degradation" (map00280), delineating enzyme(s) and/or enzymatic constituents manifesting high abundance (demarcated by a pink box) within the intestinal microbial consortium of female C57BL/6 mice subjected to a fiber-replete (F) nutritional paradigm, juxtaposed against experimental cohorts administered either fiber-devoid (NF) or fiber-devoid diets augmented with exogenous short-chain fatty acids (NF-SCFA). These biocatalytic entities orchestrate the biosynthetic transformation of alternative non-polysaccharide substrates—including proteinaceous amino acid moieties and monosaccharide metabolites synthesized by the gut microbiome, which subsequently undergo anaerobic fermentative catabolism, culminating in the generation of short-chain fatty acids. EC 5.4.99.2 = methylmalonyl-CoA mutase. Enzymes exhibiting pronounced enrichment in fiber-replete (F) dietary cohort versus fiber-devoid (NF) or exogenous SCFA-supplemented (NF-SCFA) groups are demarcated by pink boxes, whereas enzymes demonstrating pronounced enrichment in fiber-devoid (NF) cohort relative to fiber-replete (F) or SCFA-supplemented (NF-SCFA) groups are demarcated by light blue boxes.

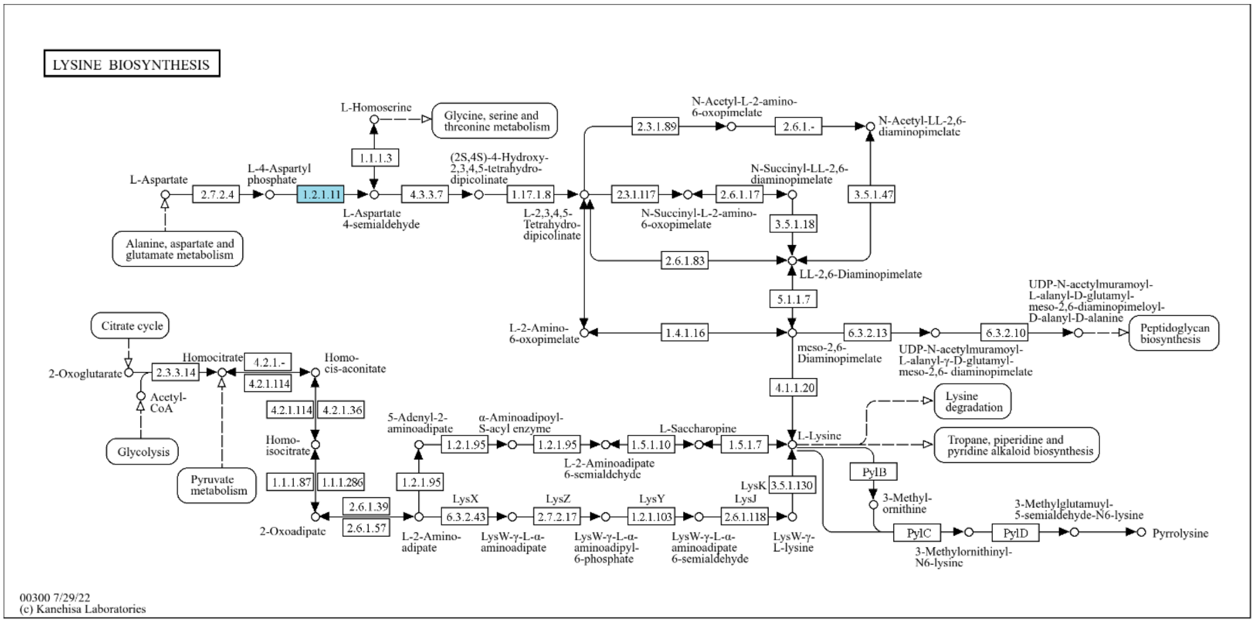

Figure S11. KEGG pathway "Lysine biosynthesis" (map00300), delineating enzyme(s) and/or enzymatic constituents manifesting high abundance (demarcated by light blue boxes) within the intestinal microbial consortium of female C57BL/6 mice subjected to a fiber-devoid (NF) nutritional paradigm, juxtaposed against experimental cohorts administered either fiber-replete (F) or fiber-devoid diets augmented with exogenous short-chain fatty acids (NF-SCFA). These biocatalytic entities orchestrate the biosynthetic transformation of alternative non-polysaccharide substrates—including proteinaceous amino acid moieties and monosaccharide metabolites synthesized by the gut microbiome, which subsequently undergo anaerobic fermentative catabolism, culminating in the generation of short-chain fatty acids. EC 1.2.1.11 = aspartate-semialdehyde dehydrogenase. Enzymes exhibiting pronounced enrichment in fiber-replete (F) dietary cohort versus fiber-devoid (NF) or exogenous SCFA-supplemented (NF-SCFA) groups are demarcated by pink boxes, whereas enzymes demonstrating pronounced enrichment in fiber-devoid (NF) cohort relative to fiber-replete (F) or SCFA-supplemented (NF-SCFA) groups are demarcated by light blue boxes.

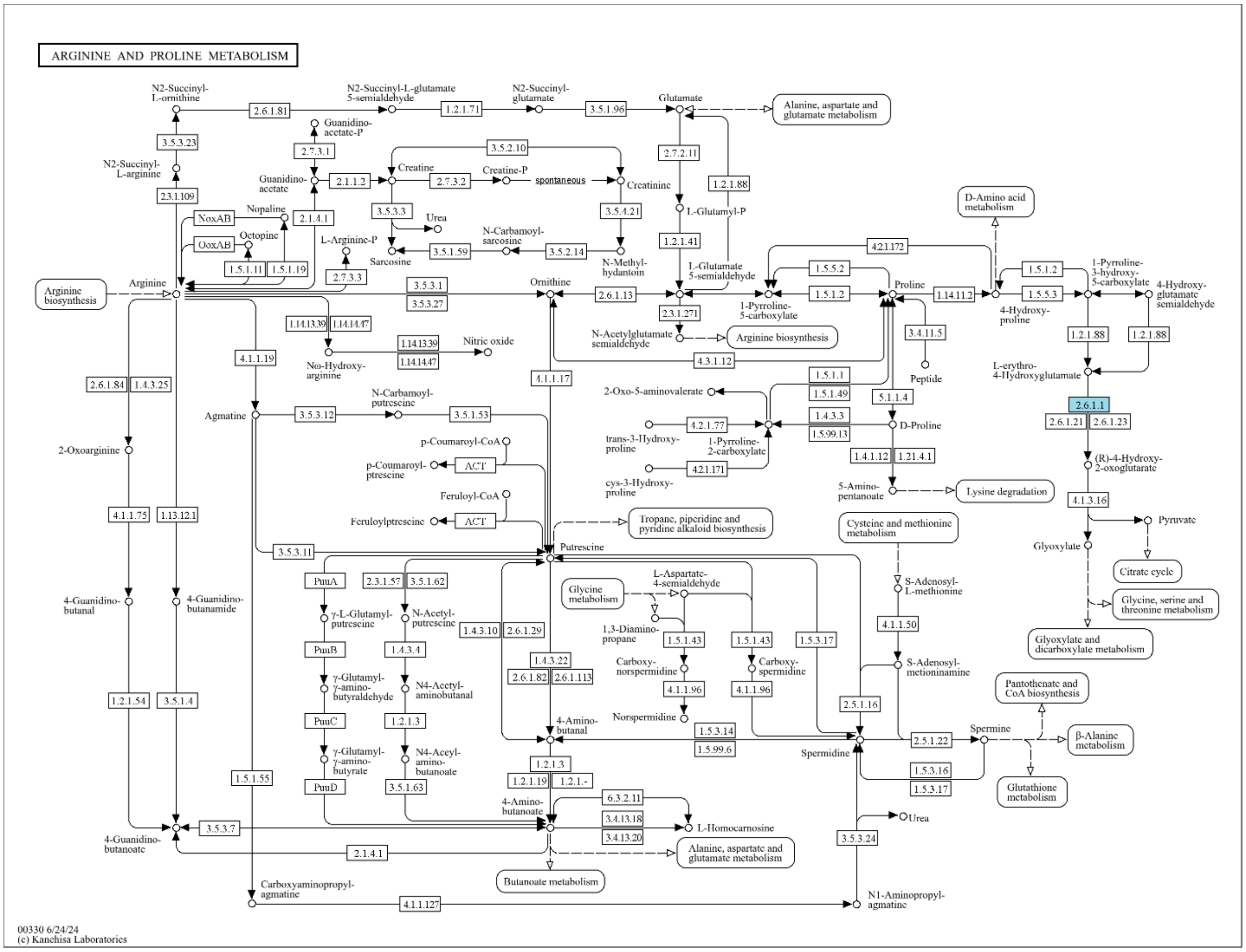

Figure S12. KEGG pathway "Arginine and proline metabolism" (map00330), delineating enzyme(s) and/or enzymatic constituents manifesting high abundance (demarcated by light blue boxes) within the intestinal microbial consortium of female C57BL/6 mice subjected to a fiber-devoid (NF) nutritional paradigm, juxtaposed against experimental cohorts administered either fiber-replete (F) or fiber-devoid diets augmented with exogenous short-chain fatty acids (NF-SCFA). These biocatalytic entities orchestrate the biosynthetic transformation of alternative non-polysaccharide substrates—including proteinaceous amino acid moieties and monosaccharide metabolites synthesized by the gut microbiome, which subsequently undergo anaerobic fermentative catabolism, culminating in the generation of short-chain fatty acids. EC 2.6.1.1 = aspartate aminotransferase. Enzymes exhibiting pronounced enrichment in fiber-replete (F) dietary cohort versus fiber-devoid (NF) or exogenous SCFA-supplemented (NF-SCFA) groups are demarcated by pink boxes, whereas enzymes demonstrating pronounced enrichment in fiber-devoid (NF) cohort relative to fiber-replete (F) or SCFA-supplemented (NF-SCFA) groups are demarcated by light blue boxes.

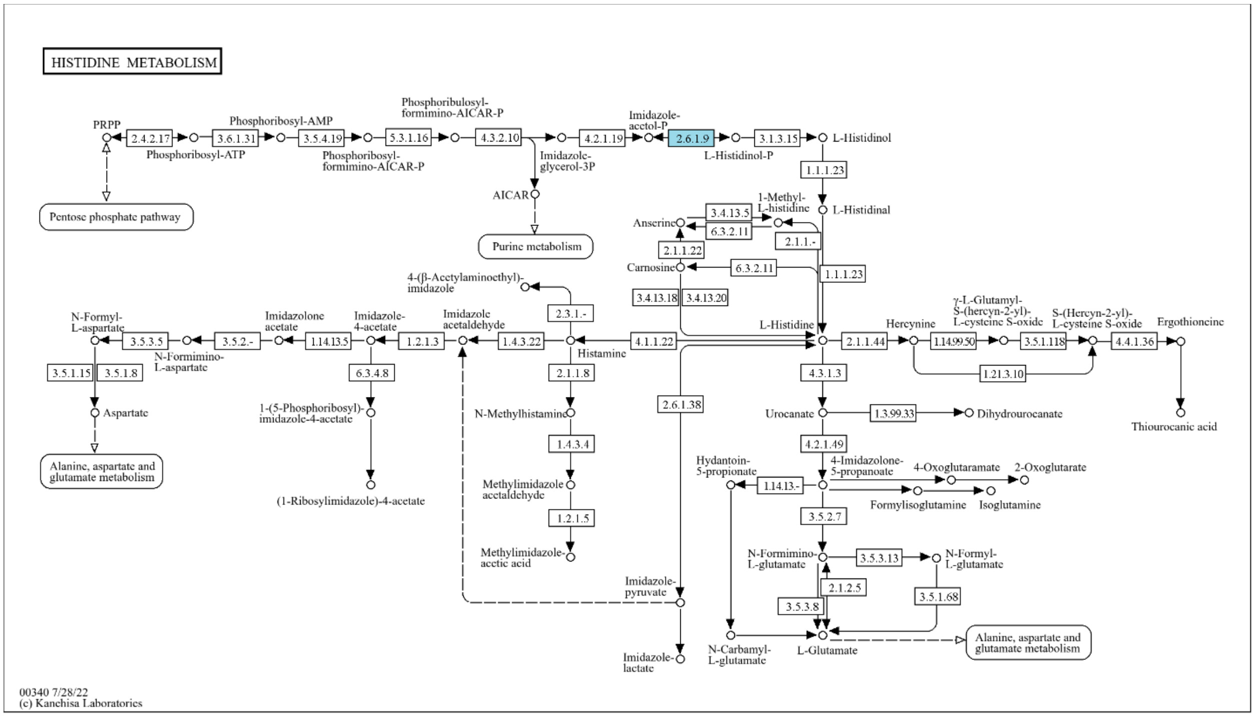

Figure S13. KEGG pathway "Histidine metabolism" (map00340), delineating enzyme(s) and/or enzymatic constituents manifesting high abundance (demarcated by light blue boxes) within the intestinal microbial consortium of female C57BL/6 mice subjected to a fiber-devoid (NF) nutritional paradigm, juxtaposed against experimental cohorts administered either fiber-replete (F) or fiber-devoid diets augmented with exogenous short-chain fatty acids (NF-SCFA). These biocatalytic entities orchestrate the biosynthetic transformation of alternative non-polysaccharide substrates—including proteinaceous amino acid moieties and monosaccharide metabolites synthesized by the gut microbiome, which subsequently undergo anaerobic fermentative catabolism, culminating in the generation of short-chain fatty acids. EC 2.6.1.9 = histidinol-phosphate aminotransferase. Enzymes exhibiting pronounced enrichment in fiber-replete (F) dietary cohort versus fiber-devoid (NF) or exogenous SCFA-supplemented (NF-SCFA) groups are demarcated by pink boxes, whereas enzymes demonstrating pronounced enrichment in fiber-devoid (NF) cohort relative to fiber-replete (F) or SCFA-supplemented (NF-SCFA) groups are demarcated by light blue boxes.

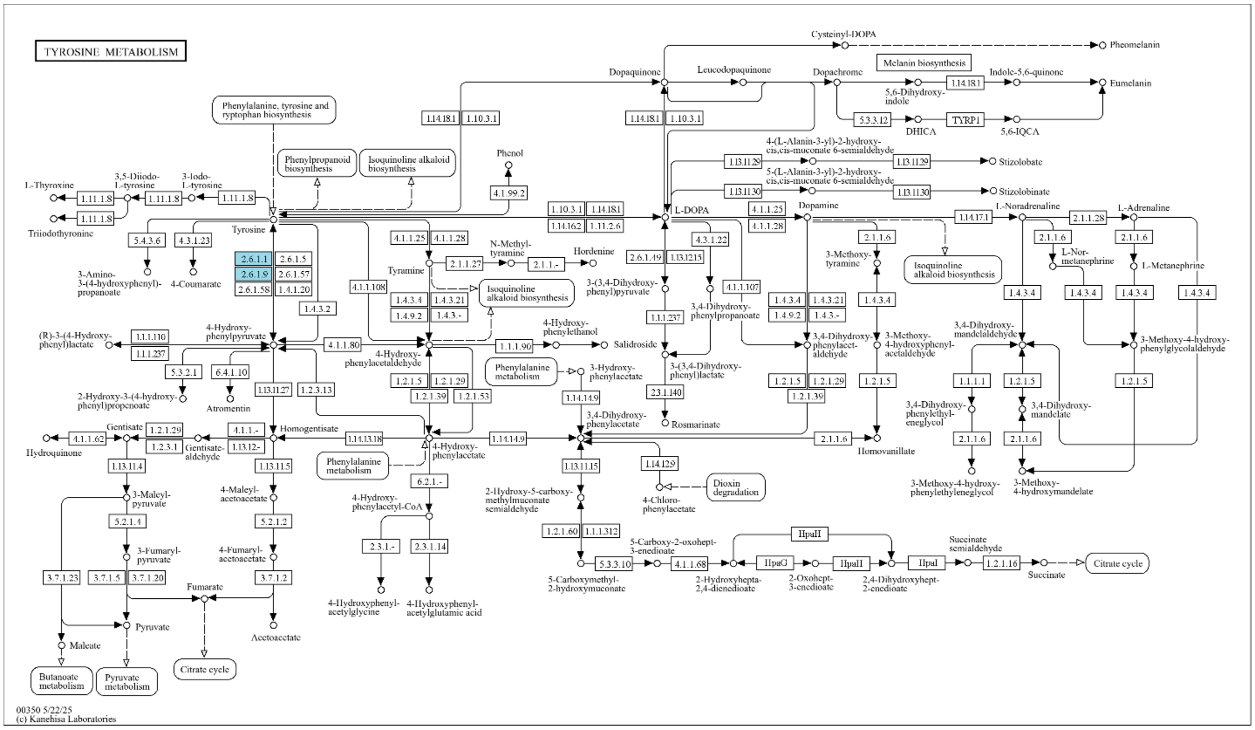

Figure S14. KEGG pathway "Tyrosine metabolism" (map00350), delineating enzyme(s) and/or enzymatic constituents manifesting high abundance (demarcated by light blue boxes) within the intestinal microbial consortium of female C57BL/6 mice subjected to a fiber-devoid (NF) nutritional paradigm, juxtaposed against experimental cohorts administered either fiber-replete (F) or fiber-devoid diets augmented with exogenous short-chain fatty acids (NF-SCFA). These biocatalytic entities orchestrate the biosynthetic transformation of alternative non-polysaccharide substrates—including proteinaceous amino acid moieties and monosaccharide metabolites synthesized by the gut microbiome, which subsequently undergo anaerobic fermentative catabolism, culminating in the generation of short-chain fatty acids. EC 2.6.1.1 = aspartate aminotransferase, EC 2.6.1.9 = histidinol-phosphate aminotransferase. Enzymes exhibiting pronounced enrichment in fiber-replete (F) dietary cohort versus fiber-devoid (NF) or exogenous SCFA-supplemented (NF-SCFA) groups are demarcated by pink boxes, whereas enzymes demonstrating pronounced enrichment in fiber-devoid (NF) cohort relative to fiber-replete (F) or SCFA-supplemented (NF-SCFA) groups are demarcated by light blue boxes.

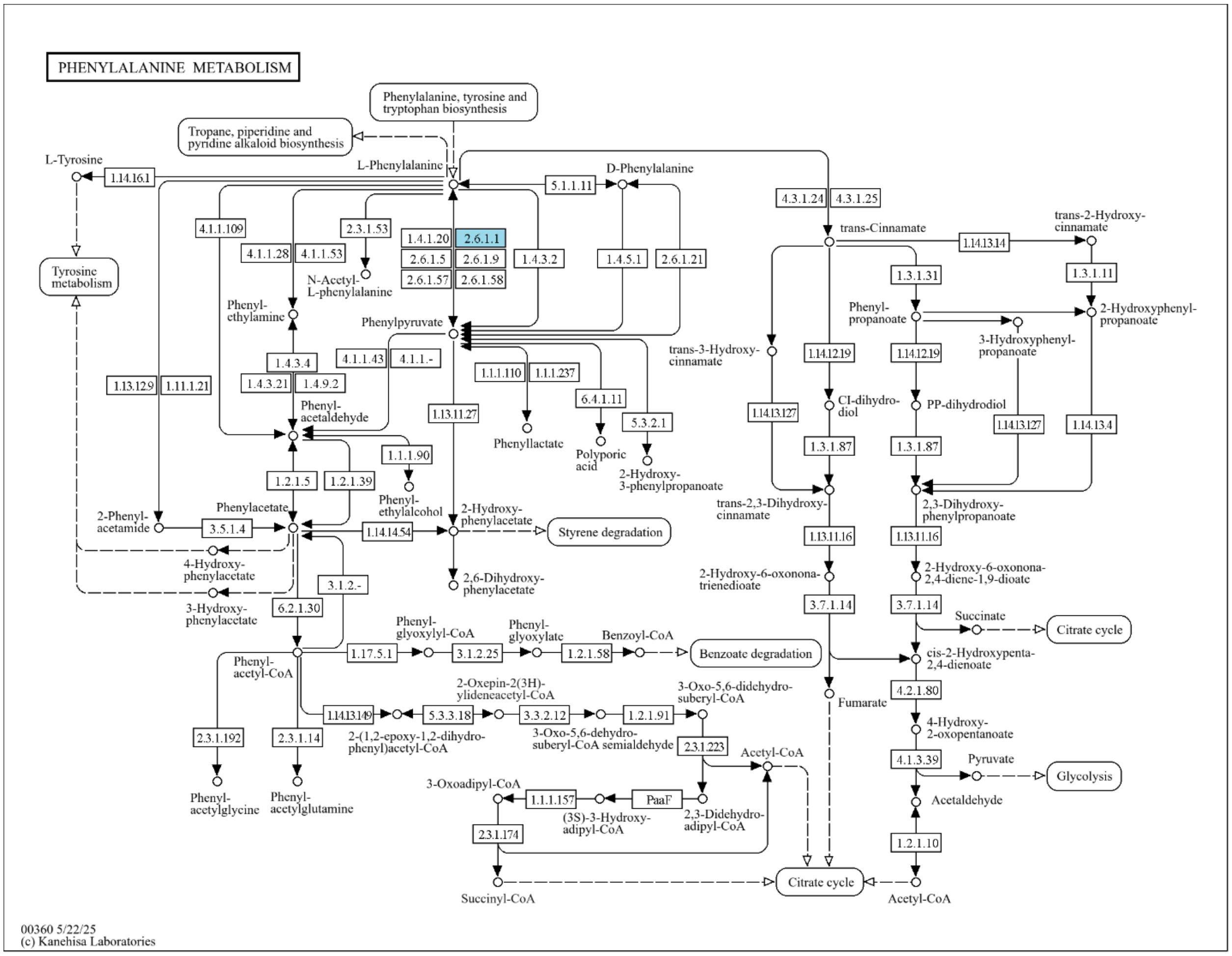

Figure S15. KEGG pathway "Phenylalanine metabolism" (map00360), delineating enzyme(s) and/or enzymatic constituents manifesting high abundance (demarcated by light blue boxes) within the intestinal microbial consortium of female C57BL/6 mice subjected to a fiber-devoid (NF) nutritional paradigm, juxtaposed against experimental cohorts administered either fiber-replete (F) or fiber-devoid diets augmented with exogenous short-chain fatty acids (NF-SCFA). These biocatalytic entities orchestrate the biosynthetic transformation of alternative non-polysaccharide substrates—including proteinaceous amino acid moieties and monosaccharide metabolites synthesized by the gut microbiome, which subsequently undergo anaerobic fermentative catabolism, culminating in the generation of short-chain fatty acids. EC 2.6.1.1 = aspartate aminotransferase. Enzymes exhibiting pronounced enrichment in fiber-replete (F) dietary cohort versus fiber-devoid (NF) or exogenous SCFA-supplemented (NF-SCFA) groups are demarcated by pink boxes, whereas enzymes demonstrating pronounced enrichment in fiber-devoid (NF) cohort relative to fiber-replete (F) or SCFA-supplemented (NF-SCFA) groups are demarcated by light blue boxes.

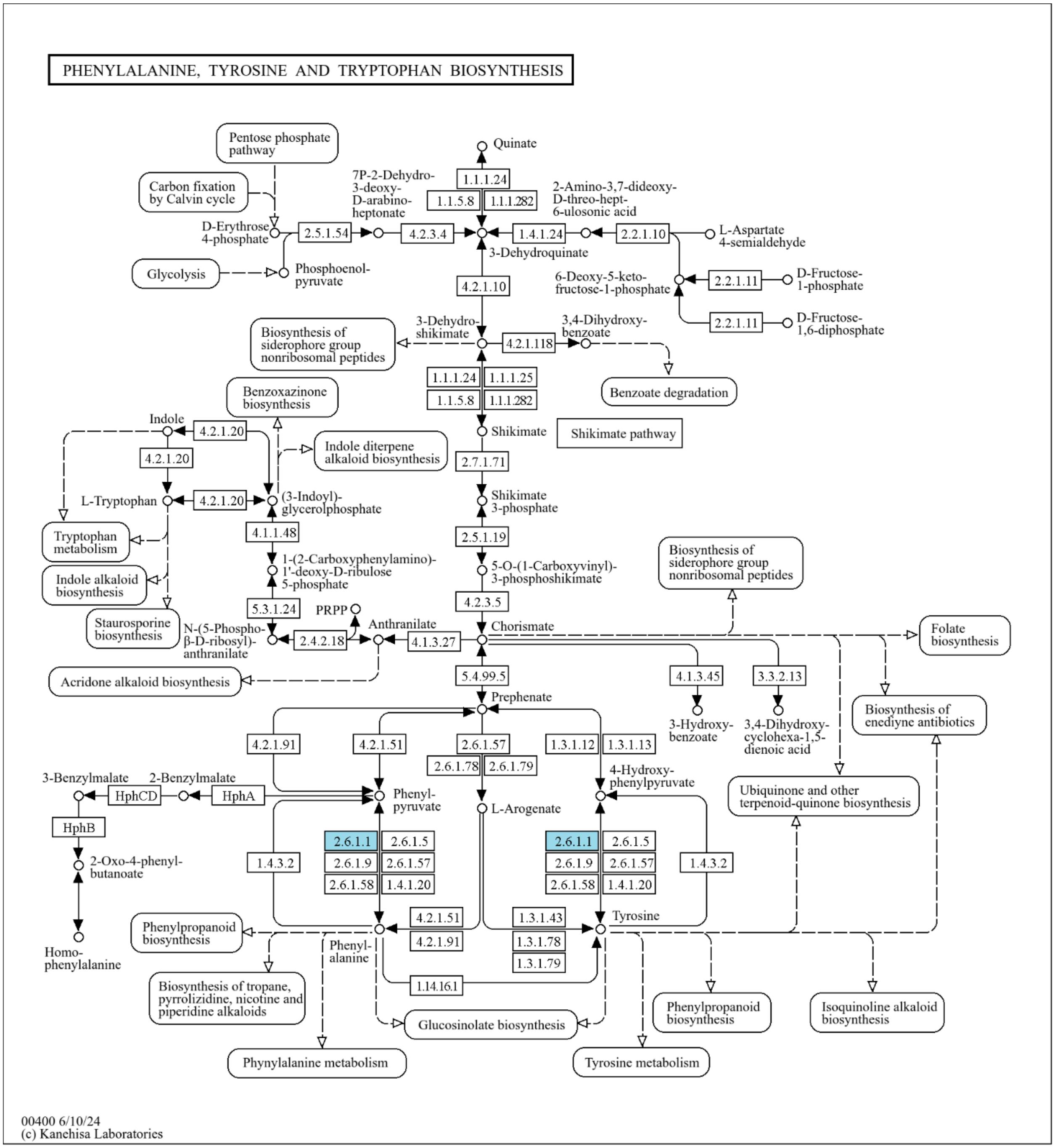

Figure S16. KEGG pathway “Phenylalanine, tyrosine and tryptophan biosynthesis” (map00400), delineating enzyme(s) and/or enzymatic constituents manifesting high abundance (demarcated by light blue boxes) within the intestinal microbial consortium of female C57BL/6 mice subjected to a fiber-devoid (NF) nutritional paradigm, juxtaposed against experimental cohorts administered either fiber-replete (F) or fiber-devoid diets augmented with exogenous short-chain fatty acids (NF-SCFA). These biocatalytic entities orchestrate the biosynthetic transformation of alternative non-polysaccharide substrates—including proteinaceous amino acid moieties and monosaccharide metabolites synthesized by the gut microbiome, which subsequently undergo anaerobic fermentative catabolism, culminating in the generation of short-chain fatty acids. EC 2.6.1.1 = aspartate aminotransferase. Enzymes exhibiting pronounced enrichment in fiber-replete (F) dietary cohort versus fiber-devoid (NF) or exogenous SCFA-supplemented (NF-SCFA) groups are demarcated by pink boxes, whereas enzymes demonstrating pronounced enrichment in fiber-devoid (NF) cohort relative to fiber-replete (F) or SCFA-supplemented (NF-SCFA) groups are demarcated by light blue boxes.

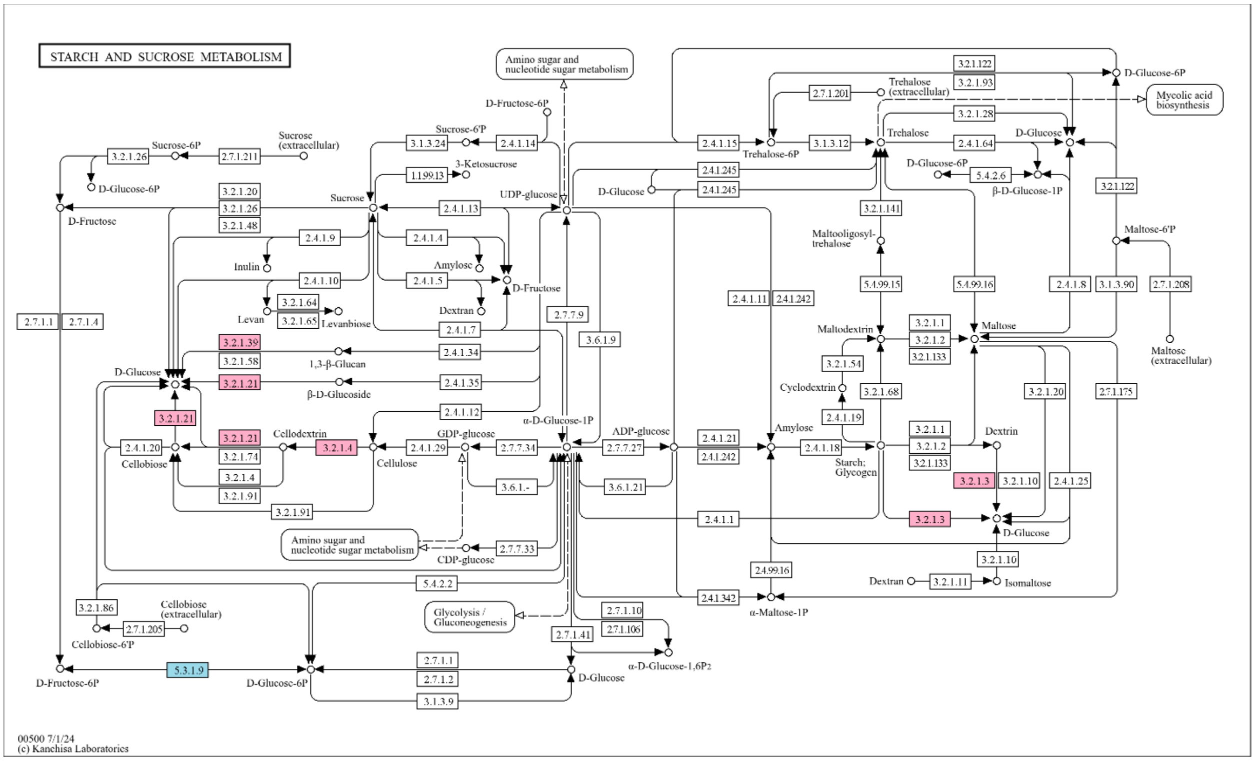

Figure S17. KEGG pathway "Starch and sucrose metabolism" (map00500), delineating enzyme(s) and/or enzymatic constituents manifesting high abundance (demarcated by light blue boxes) within the intestinal microbial consortium of female C57BL/6 mice subjected to a fiber-devoid (NF) nutritional paradigm, juxtaposed against experimental cohorts administered either fiber-replete (F) or fiber-devoid diets augmented with exogenous short-chain fatty acids (NF-SCFA). These biocatalytic entities orchestrate the biosynthetic transformation of alternative non-polysaccharide substrates—including proteinaceous amino acid moieties and monosaccharide metabolites synthesized by the gut microbiome, which subsequently undergo anaerobic fermentative catabolism, culminating in the generation of short-chain fatty acids. EC 3.2.1.21 = beta-glucosidase, EC 3.2.1.3 = glucan 1,4-alpha-glucosidase, EC 3.2.1.4 = cellulase, EC 3.2.1.39 = glucan endo-1,3-beta-D-glucosidase, EC 5.3.1.9 = glucose-6-phosphate isomerase. Enzymes exhibiting pronounced enrichment in fiber-replete (F) dietary cohort versus fiber-devoid (NF) or exogenous SCFA-supplemented (NF-SCFA) groups are demarcated by pink boxes, whereas enzymes demonstrating pronounced enrichment in fiber-devoid (NF) cohort relative to fiber-replete (F) or SCFA-supplemented (NF-SCFA) groups are demarcated by light blue boxes.

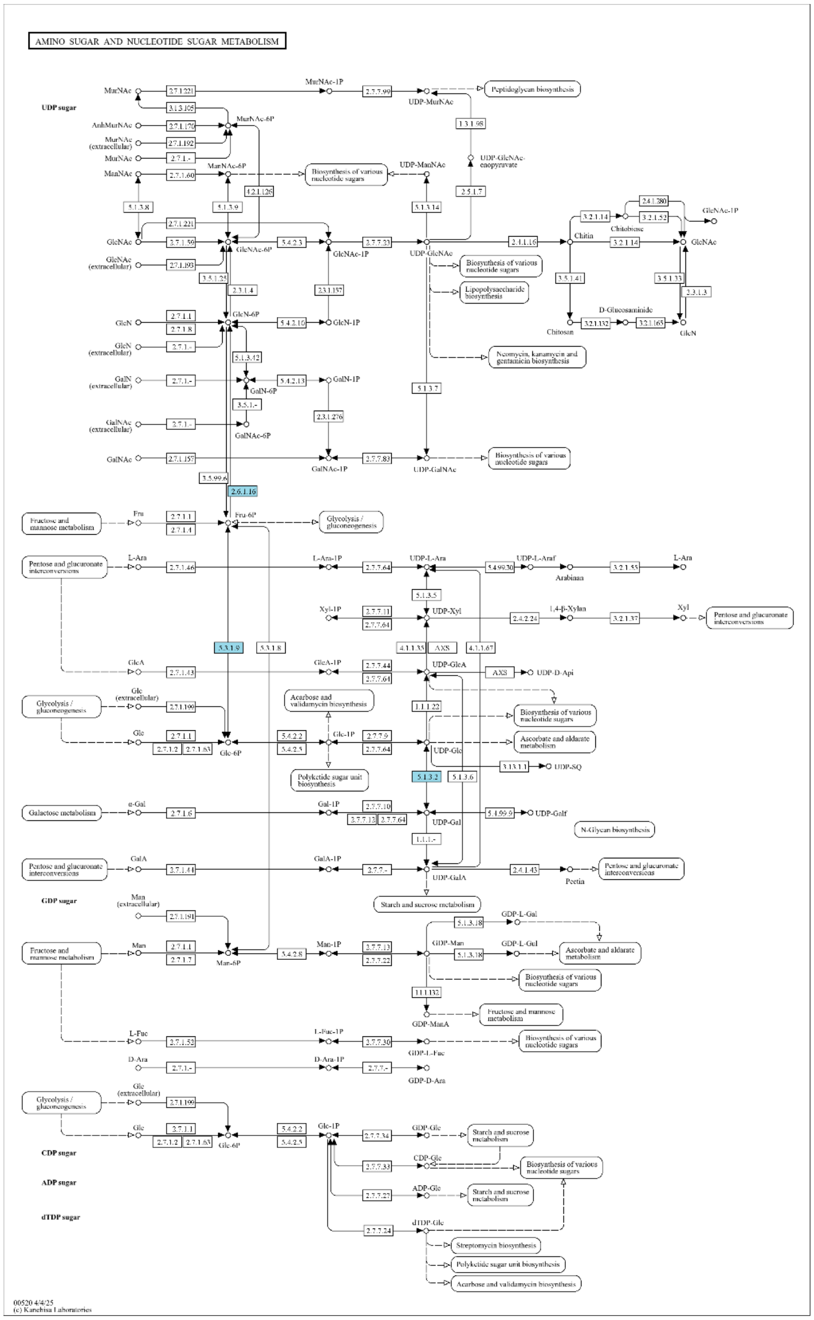

Figure S18. KEGG pathway “Amino sugar and nucleotide sugar metabolism” (map00520), delineating enzyme(s) and/or enzymatic constituents manifesting high abundance (demarcated by light blue boxes) within the intestinal microbial consortium of female C57BL/6 mice subjected to a fiber-devoid (NF) nutritional paradigm, juxtaposed against experimental cohorts administered either fiber-replete (F) or fiber-devoid diets augmented with exogenous short-chain fatty acids (NF-SCFA). These biocatalytic entities orchestrate the biosynthetic transformation of alternative non-polysaccharide substrates—including proteinaceous amino acid moieties and monosaccharide metabolites synthesized by the gut microbiome, which subsequently undergo anaerobic fermentative catabolism, culminating in the generation of short-chain fatty acids. EC 5.3.1.9 = glucose-6-phosphate isomerase, EC 5.1.3.2 = UDP-glucose 4-epimerase, EC 2.6.1.16 = glutamine--fructose-6-phosphate transaminase. Enzymes exhibiting pronounced enrichment in fiber-replete (F) dietary cohort versus fiber-devoid (NF) or exogenous SCFA-supplemented (NF-SCFA) groups are demarcated by pink boxes, whereas enzymes demonstrating pronounced enrichment in fiber-devoid (NF) cohort relative to fiber-replete (F) or SCFA-supplemented (NF-SCFA) groups are demarcated by light blue boxes.

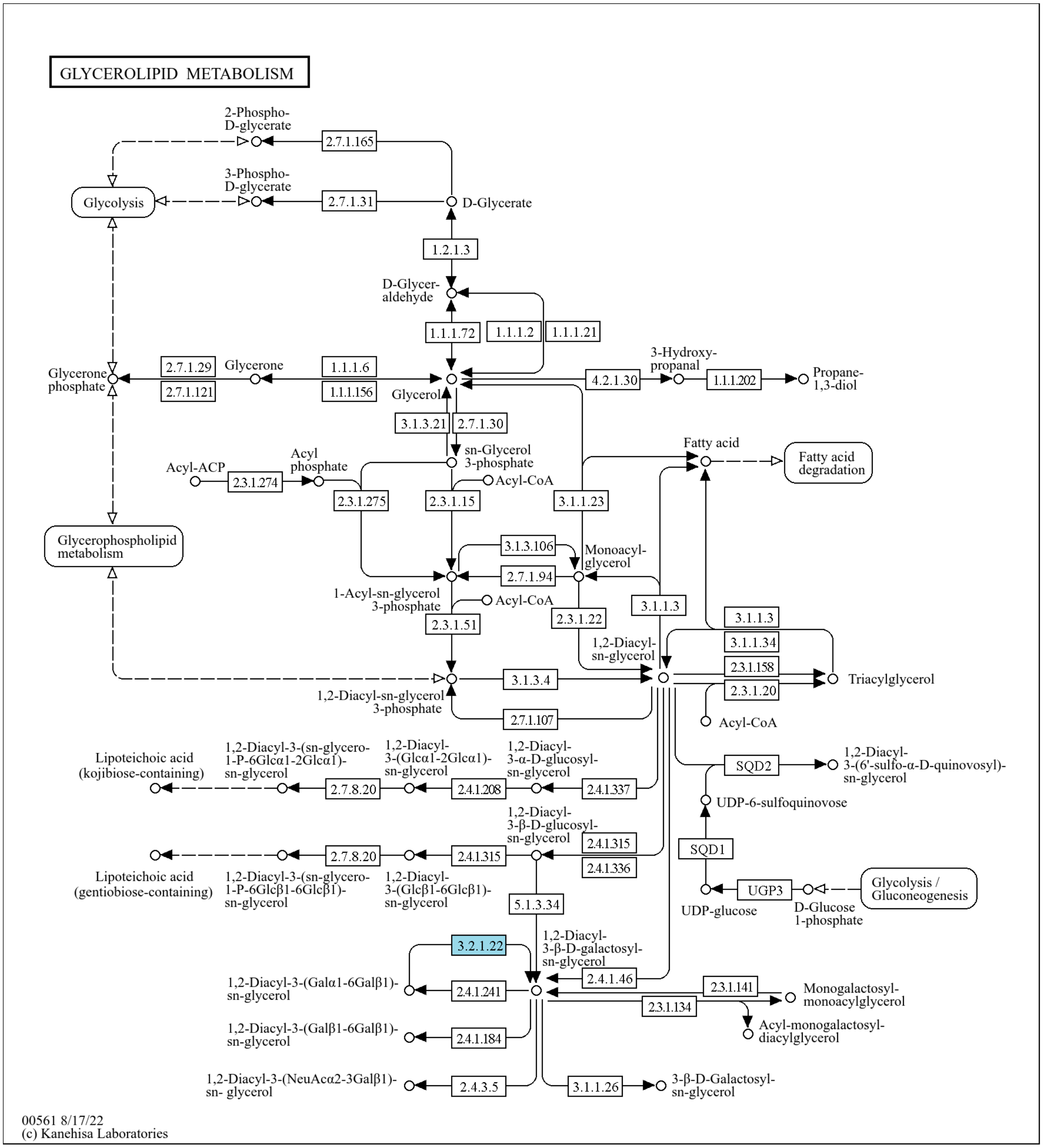

Figure S19. KEGG pathway "Glycerolipid metabolism" (map00561), delineating enzyme(s) and/or enzymatic constituents manifesting high abundance (demarcated by light blue boxes) within the intestinal microbial consortium of female C57BL/6 mice subjected to a fiber-devoid (NF) nutritional paradigm, juxtaposed against experimental cohorts administered either fiber-replete (F) or fiber-devoid diets augmented with exogenous short-chain fatty acids (NF-SCFA). These biocatalytic entities orchestrate the biosynthetic transformation of alternative non-polysaccharide substrates—including proteinaceous amino acid moieties and monosaccharide metabolites synthesized by the gut microbiome, which subsequently undergo anaerobic fermentative catabolism, culminating in the generation of short-chain fatty acids. EC 3.2.1.22 = alpha-galactosidase. Enzymes exhibiting pronounced enrichment in fiber-replete (F) dietary cohort versus fiber-devoid (NF) or exogenous SCFA-supplemented (NF-SCFA) groups are demarcated by pink boxes, whereas enzymes demonstrating pronounced enrichment in fiber-devoid (NF) cohort relative to fiber-replete (F) or SCFA-supplemented (NF-SCFA) groups are demarcated by light blue boxes.

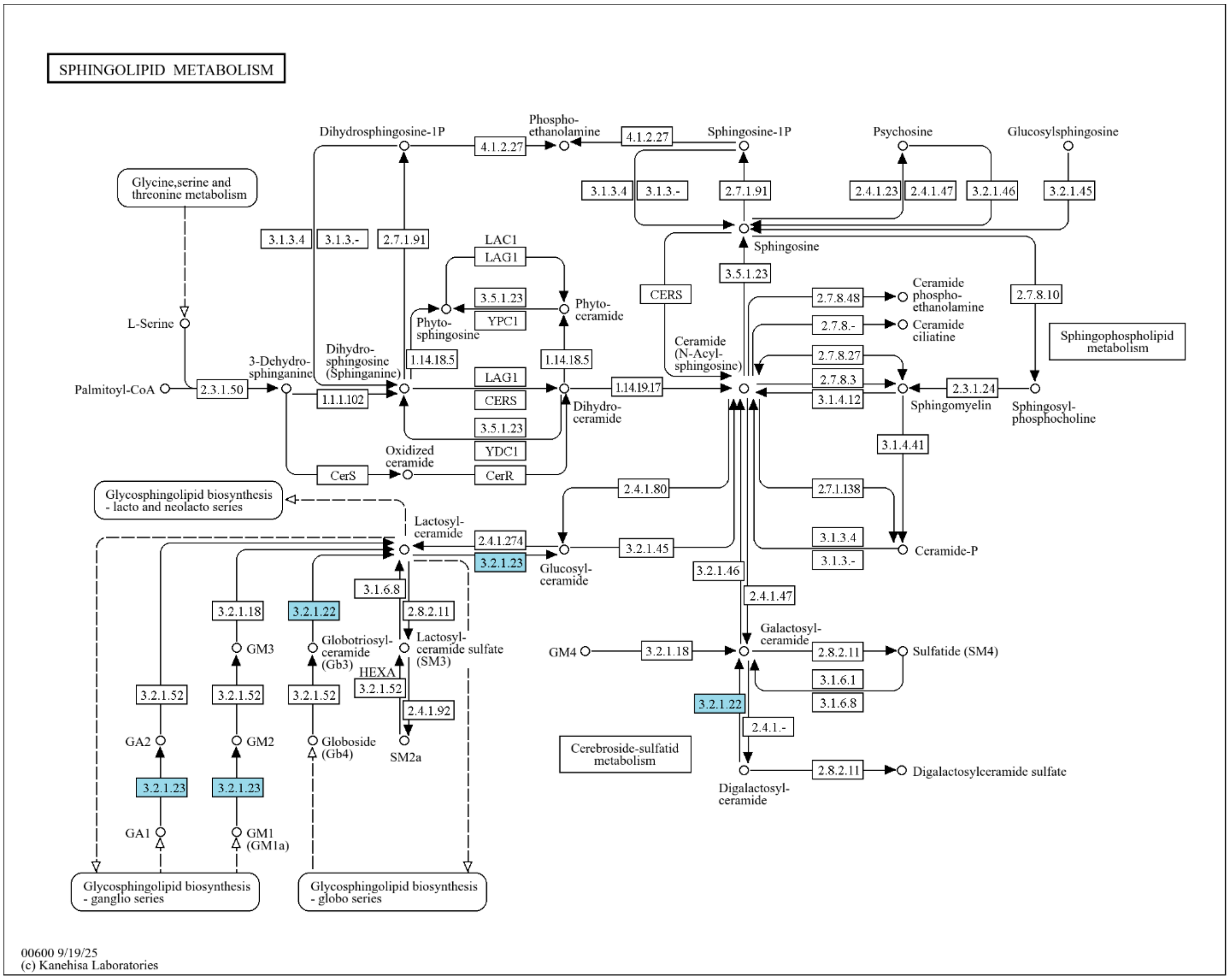

Figure S20. KEGG pathway "Sphingolipid metabolism" (map00600), delineating enzyme(s) and/or enzymatic constituents manifesting high abundance (demarcated by light blue boxes) within the intestinal microbial consortium of female C57BL/6 mice subjected to a fiber-devoid (NF) nutritional paradigm, juxtaposed against experimental cohorts administered either fiber-replete (F) or fiber-devoid diets augmented with exogenous short-chain fatty acids (NF-SCFA). These biocatalytic entities orchestrate the biosynthetic transformation of alternative non-polysaccharide substrates—including proteinaceous amino acid moieties and monosaccharide metabolites synthesized by the gut microbiome, which subsequently undergo anaerobic fermentative catabolism, culminating in the generation of short-chain fatty acids. EC 3.2.1.23 = beta-galactosidase, EC 3.2.1.22 = alpha-galactosidase. Enzymes exhibiting pronounced enrichment in fiber-replete (F) dietary cohort versus fiber-devoid (NF) or exogenous SCFA-supplemented (NF-SCFA) groups are demarcated by pink boxes, whereas enzymes demonstrating pronounced enrichment in fiber-devoid (NF) cohort relative to fiber-replete (F) or SCFA-supplemented (NF-SCFA) groups are demarcated by light blue boxes.

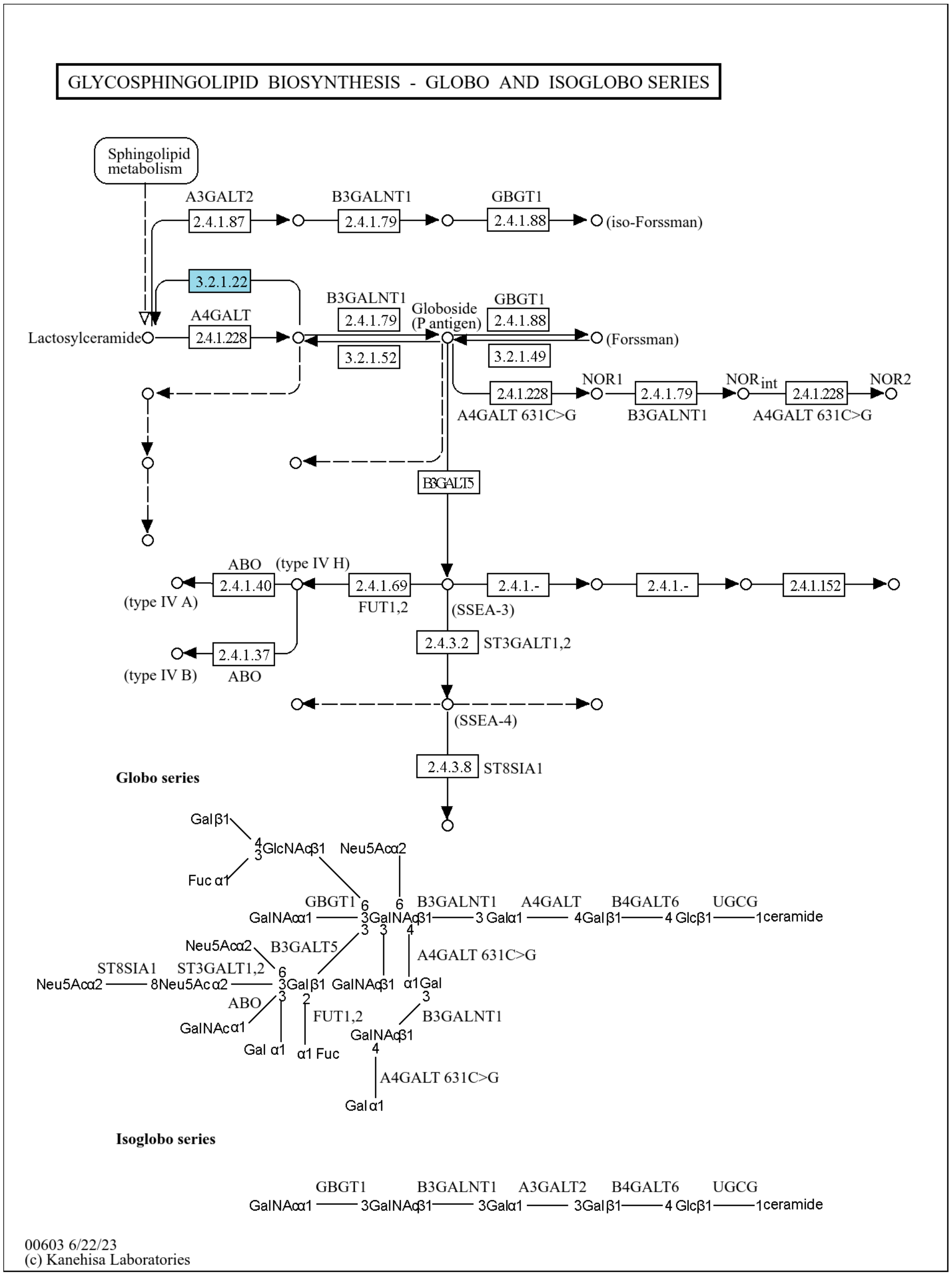

Figure S21. KEGG pathway "Glycosphingolipid biosynthesis - globo and isoglobo series" (map00603), delineating enzyme(s) and/or enzymatic constituents manifesting high abundance (demarcated by light blue boxes) within the intestinal microbial consortium of female C57BL/6 mice subjected to a fiber-devoid (NF) nutritional paradigm, juxtaposed against experimental cohorts administered either fiber-replete (F) or fiber-devoid diets augmented with exogenous short-chain fatty acids (NF-SCFA). These biocatalytic entities orchestrate the biosynthetic transformation of alternative non-polysaccharide substrates—including proteinaceous amino acid moieties and monosaccharide metabolites synthesized by the gut microbiome, which subsequently undergo anaerobic fermentative catabolism, culminating in the generation of short-chain fatty acids. EC 3.2.1.22 = alpha-galactosidase. Enzymes exhibiting pronounced enrichment in fiber-replete (F) dietary cohort versus fiber-devoid (NF) or exogenous SCFA-supplemented (NF-SCFA) groups are demarcated by pink boxes, whereas enzymes demonstrating pronounced enrichment in fiber-devoid (NF) cohort relative to fiber-replete (F) or SCFA-supplemented (NF-SCFA) groups are demarcated by light blue boxes.

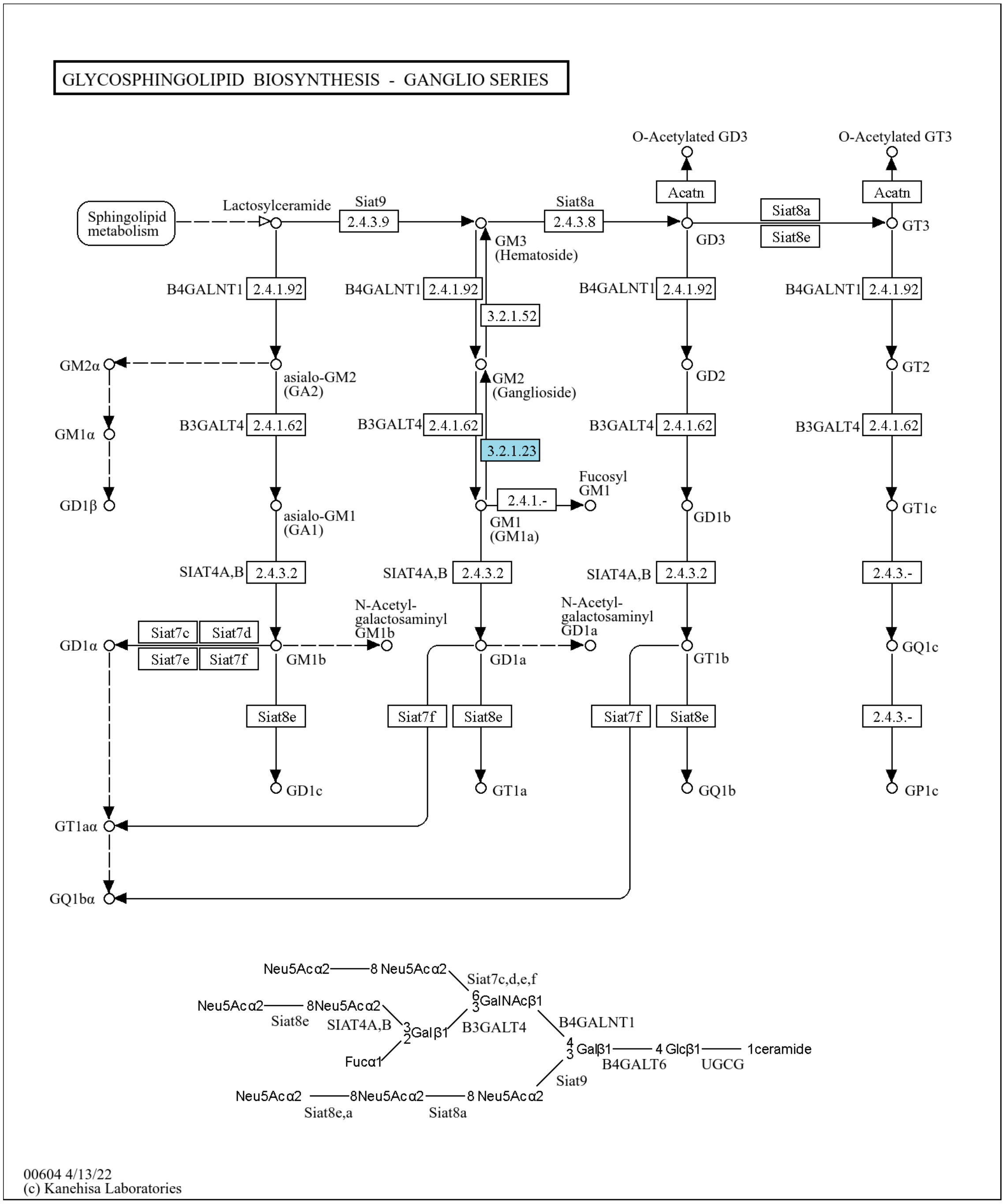

Figure S22. KEGG pathway "Glycosphingolipid biosynthesis - ganglio series" (map00604), delineating enzyme(s) and/or enzymatic constituents manifesting high abundance (demarcated by light blue boxes) within the intestinal microbial consortium of female C57BL/6 mice subjected to a fiber-devoid (NF) nutritional paradigm, juxtaposed against experimental cohorts administered either fiber-replete (F) or fiber-devoid diets augmented with exogenous short-chain fatty acids (NF-SCFA). These biocatalytic entities orchestrate the biosynthetic transformation of alternative non-polysaccharide substrates—including proteinaceous amino acid moieties and monosaccharide metabolites synthesized by the gut microbiome, which subsequently undergo anaerobic fermentative catabolism, culminating in the generation of short-chain fatty acids. EC 3.2.1.23 = beta-galactosidase. Enzymes exhibiting pronounced enrichment in fiber-replete (F) dietary cohort versus fiber-devoid (NF) or exogenous SCFA-supplemented (NF-SCFA) groups are demarcated by pink boxes, whereas enzymes demonstrating pronounced enrichment in fiber-devoid (NF) cohort relative to fiber-replete (F) or SCFA-supplemented (NF-SCFA) groups are demarcated by light blue boxes.

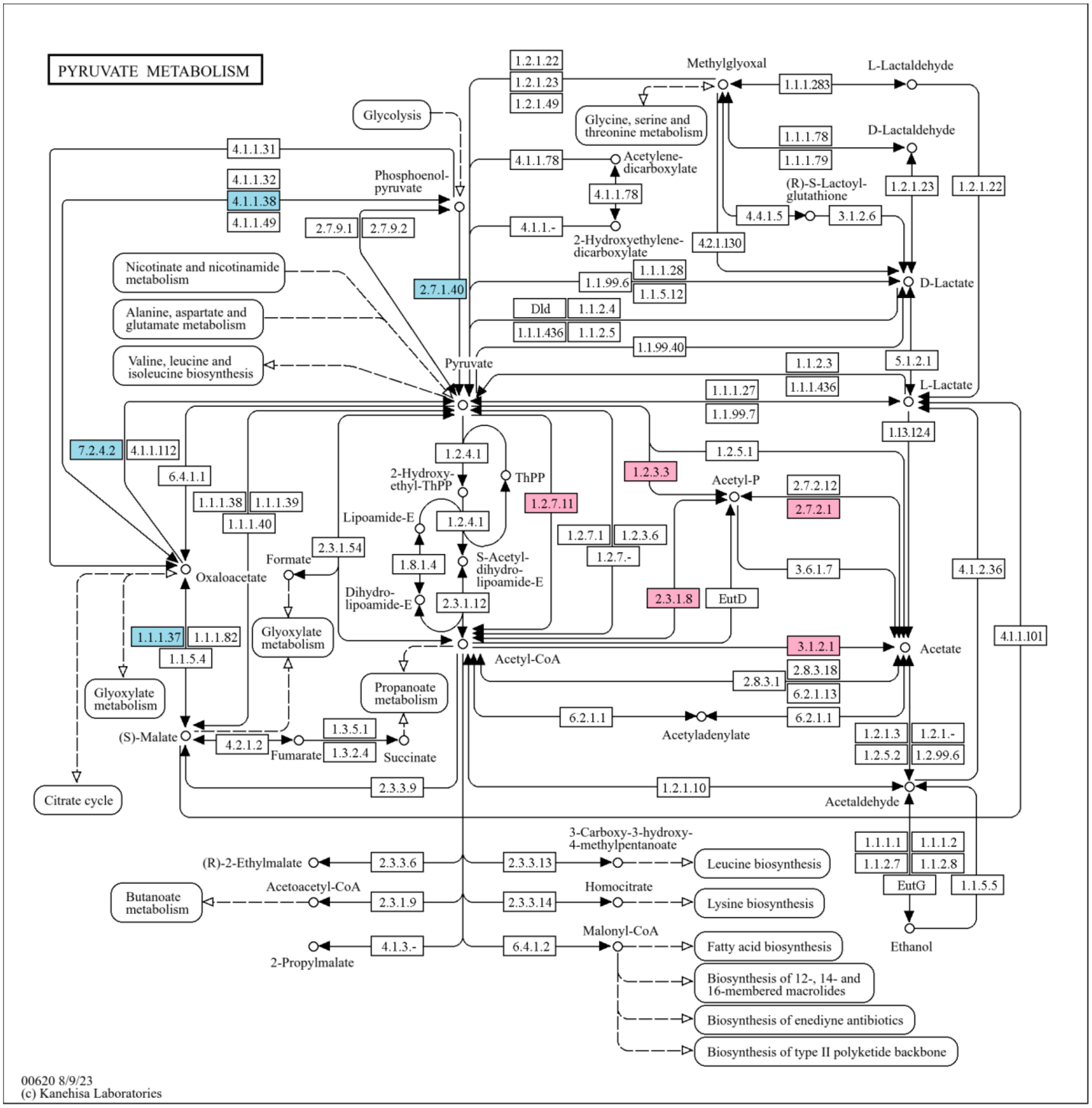

Figure S23. KEGG pathway "Pyruvate metabolism" (map00620), delineating enzyme(s) and/or enzymatic constituents manifesting high abundance within the intestinal microbial consortium of female C57BL/6 mice subjected to either fiber-replete (F, demarcated by pink boxes) or fiber-devoid (NF, demarcated by light blue boxes) dietary regimens, contrasted with an experimental cohort administered a fiber-devoid diet augmented with exogenous short-chain fatty acids (NF-SCFA). These biocatalytic entities orchestrate the biosynthetic transformation of alternative non-polysaccharide substrates—including proteinaceous amino acid moieties and monosaccharide metabolites synthesized by the gut microbiome, which subsequently undergo anaerobic fermentative catabolism, culminating in the generation of short-chain fatty acids. EC 2.7.2.1 = acetate kinase, EC 2.3.1.8 = phosphate acetyltransferase, EC 2.7.1.40 = pyruvate kinase, EC 4.1.1.38 = phosphoenolpyruvate carboxykinase (diphosphate), EC 1.2.3.3 = pyruvate oxidase, EC 3.1.2.1 = acetyl-CoA hydrolase, EC 7.2.4.2 = oxaloacetate decarboxylase (Na+ extruding), EC 1.2.7.11 = 2-oxoglutarate/2-oxoacid ferredoxin oxidoreductase subunit alpha, EC 1.1.1.37 = malate dehydrogenase. Enzymes exhibiting pronounced enrichment in fiber-replete (F) dietary cohort versus fiber-devoid (NF) or exogenous SCFA-supplemented (NF-SCFA) groups are demarcated by pink boxes, whereas enzymes demonstrating pronounced enrichment in fiber-devoid (NF) cohort relative to fiber-replete (F) or SCFA-supplemented (NF-SCFA) groups are demarcated by light blue boxes.

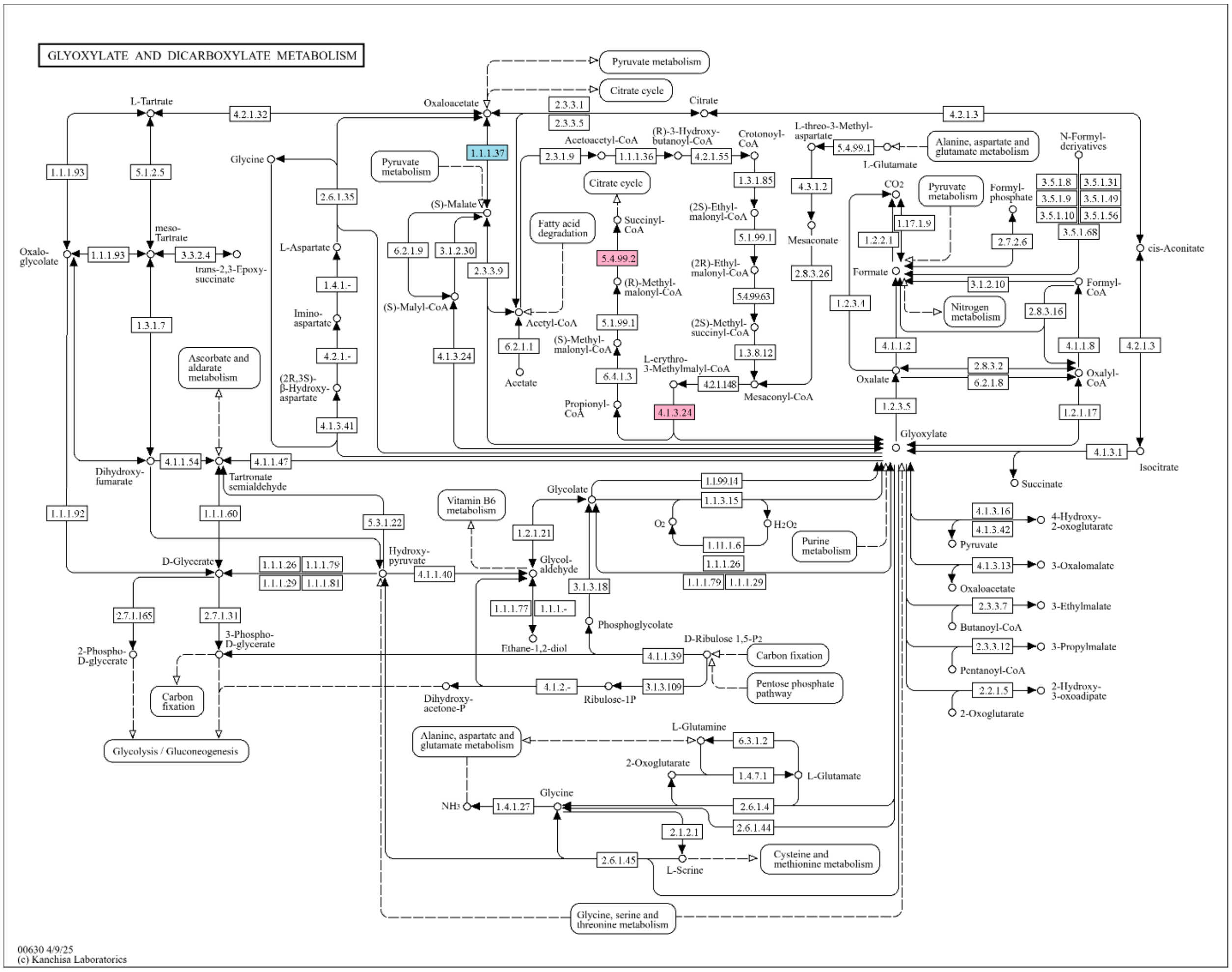

Figure S24. KEGG pathway "Glyoxylate and dicarboxylate metabolism" (map00630), delineating enzyme(s) and/or enzymatic constituents manifesting high abundance within the intestinal microbial consortium of female C57BL/6 mice subjected to either fiber-replete (F, demarcated by a pink box) or fiber-devoid (NF, demarcated by a light blue box) dietary regimens, contrasted with an experimental cohort administered a fiber-devoid diet augmented with exogenous short-chain fatty acids (NF-SCFA). These biocatalytic entities orchestrate the biosynthetic transformation of alternative non-polysaccharide substrates—including proteinaceous amino acid moieties and monosaccharide metabolites synthesized by the gut microbiome, which subsequently undergo anaerobic fermentative catabolism, culminating in the generation of short-chain fatty acids. EC 1.1.1.37 = malate dehydrogenase, EC 5.4.99.2 = methylmalonyl-CoA mutase, EC 4.1.3.24 = malyl-CoA lyase. Enzymes exhibiting pronounced enrichment in fiber-replete (F) dietary cohort versus fiber-devoid (NF) or exogenous SCFA-supplemented (NF-SCFA) groups are demarcated by pink boxes, whereas enzymes demonstrating pronounced enrichment in fiber-devoid (NF) cohort relative to fiber-replete (F) or SCFA-supplemented (NF-SCFA) groups are demarcated by light blue boxes.

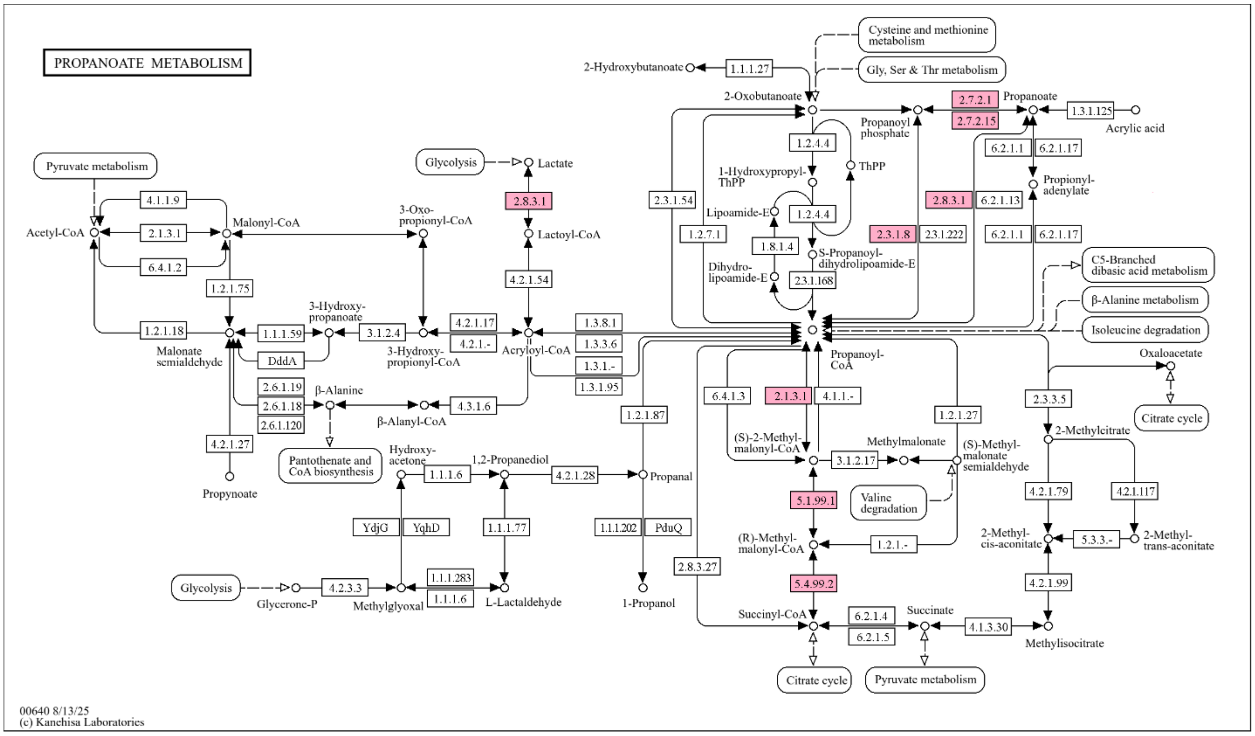

Figure S25. KEGG pathway "Propanoate metabolism" (map00640), delineating enzyme(s) and/or enzymatic constituents manifesting high abundance (demarcated by pink boxes) within the intestinal microbial consortium of female C57BL/6 mice subjected to a fiber-replete (F) nutritional paradigm, juxtaposed against experimental cohorts administered either fiber-devoid (NF) or fiber-devoid diets augmented with exogenous short-chain fatty acids (NF-SCFA). These biocatalytic entities orchestrate the biosynthetic transformation of alternative non-polysaccharide substrates—including proteinaceous amino acid moieties and monosaccharide metabolites synthesized by the gut microbiome, which subsequently undergo anaerobic fermentative catabolism, culminating in the generation of short-chain fatty acids. EC 2.7.2.1 = acetate kinase, EC 2.3.1.8 = phosphate acetyltransferase, EC 2.8.3.1 = propionate CoA-transferase, EC 5.1.99.1 = methylmalonyl-CoA epimerase, EC 2.1.3.1 = methylmalonyl-CoA carboxytransferase, EC 5.4.99.2 = methylmalonyl-CoA mutase, EC 2.7.2.15 = propionate kinase. Enzymes exhibiting pronounced enrichment in fiber-replete (F) dietary cohort versus fiber-devoid (NF) or exogenous SCFA-supplemented (NF-SCFA) groups are demarcated by pink boxes, whereas enzymes demonstrating pronounced enrichment in fiber-devoid (NF) cohort relative to fiber-replete (F) or SCFA-supplemented (NF-SCFA) groups are demarcated by light blue boxes.

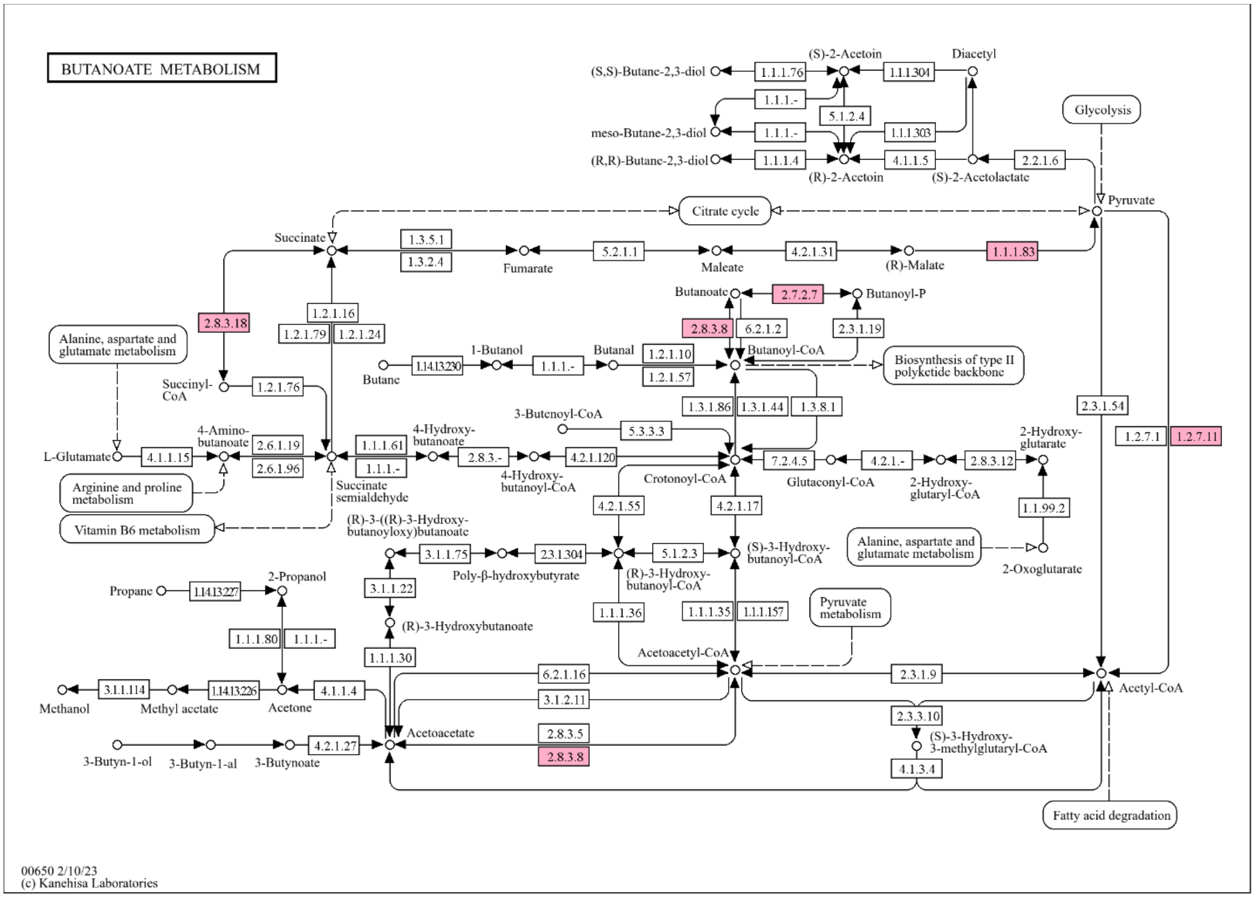

Figure S26. KEGG pathway "Butanoate metabolism" (map00650), delineating enzyme(s) and/or enzymatic constituents manifesting high abundance (demarcated by pink boxes) within the intestinal microbial consortium of female C57BL/6 mice subjected to a fiber-replete (F) nutritional paradigm, juxtaposed against experimental cohorts administered either fiber-devoid (NF) or fiber-devoid diets augmented with exogenous short-chain fatty acids (NF-SCFA). These biocatalytic entities orchestrate the biosynthetic transformation of alternative non-polysaccharide substrates—including proteinaceous amino acid moieties and monosaccharide metabolites synthesized by the gut microbiome, which subsequently undergo anaerobic fermentative catabolism, culminating in the generation of short-chain fatty acids. EC 1.2.7.11 = 2-oxoglutarate/2-oxoacid ferredoxin oxidoreductase subunit alpha, EC 1.1.1.83 = D-malate dehydrogenase (decarboxylating), EC 2.7.2.7 = butyrate kinase, EC 2.8.3.8 = acetate CoA-transferase / butyryl CoA:acetate CoA transferase, EC 2.8.3.18 = succinyl-CoA:acetate CoA-transferase. Enzymes exhibiting pronounced enrichment in fiber-replete (F) dietary cohort versus fiber-devoid (NF) or exogenous SCFA-supplemented (NF-SCFA) groups are demarcated by pink boxes, whereas enzymes demonstrating pronounced enrichment in fiber-devoid (NF) cohort relative to fiber-replete (F) or SCFA-supplemented (NF-SCFA) groups are demarcated by light blue boxes.

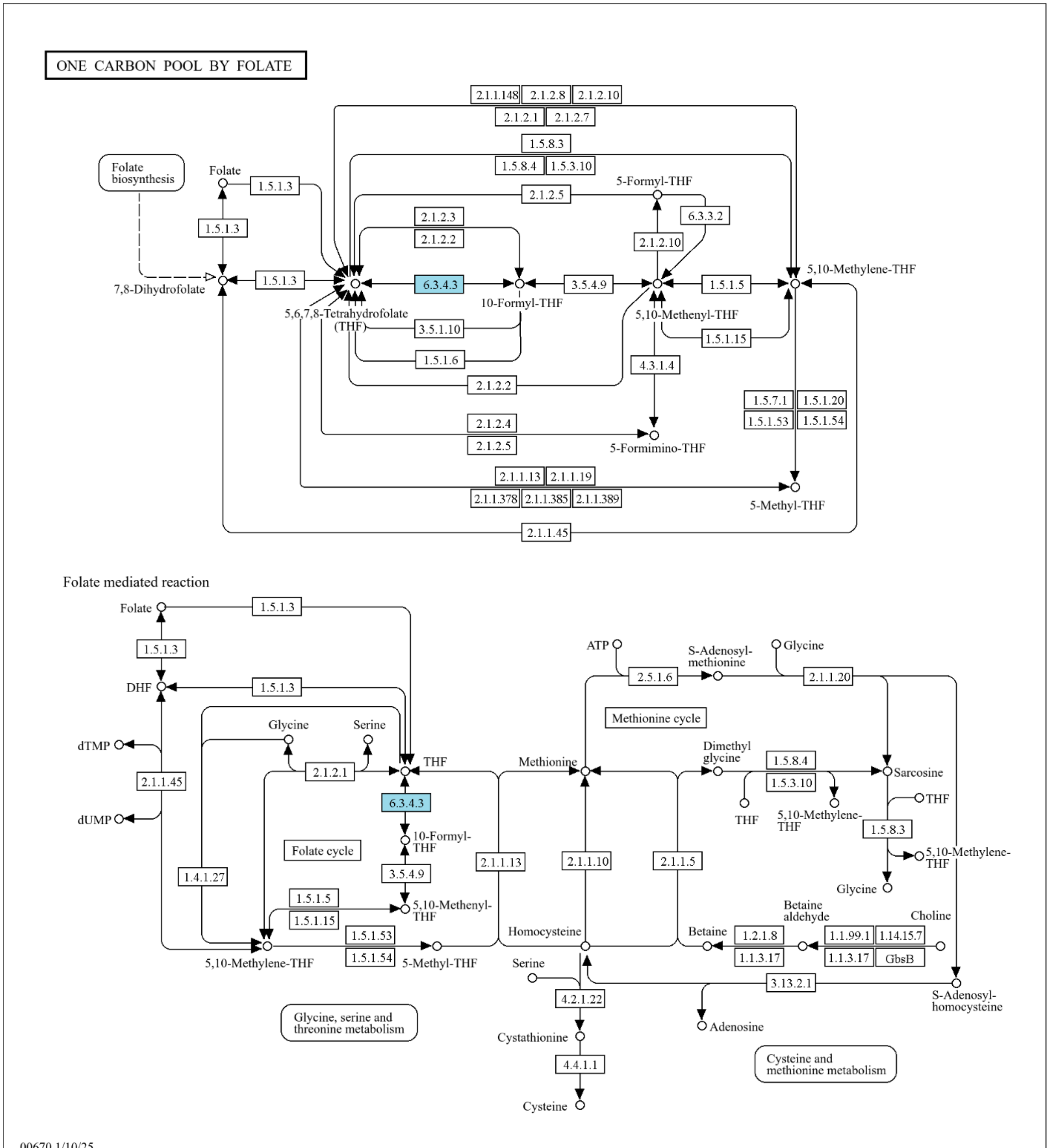

Figure S27. KEGG pathway "One carbon pool by folate" (map00670), delineating enzyme(s) and/or enzymatic constituents manifesting high abundance (demarcated by light blue boxes) within the intestinal microbial consortium of female C57BL/6 mice subjected to a fiber-devoid (NF) nutritional paradigm, juxtaposed against experimental cohorts administered either fiber-replete (F) or fiber-devoid diets augmented with exogenous short-chain fatty acids (NF-SCFA). These biocatalytic entities orchestrate the biosynthetic transformation of alternative non-polysaccharide substrates—including proteinaceous amino acid moieties and monosaccharide metabolites synthesized by the gut microbiome, which subsequently undergo anaerobic fermentative catabolism, culminating in the generation of short-chain fatty acids. EC 6.3.4.3 = formate--tetrahydrofolate ligase. Enzymes exhibiting pronounced enrichment in fiber-replete (F) dietary cohort versus fiber-devoid (NF) or exogenous SCFA-supplemented (NF-SCFA) groups are demarcated by pink boxes, whereas enzymes demonstrating pronounced enrichment in fiber-devoid (NF) cohort relative to fiber-replete (F) or SCFA-supplemented (NF-SCFA) groups are demarcated by light blue boxes.

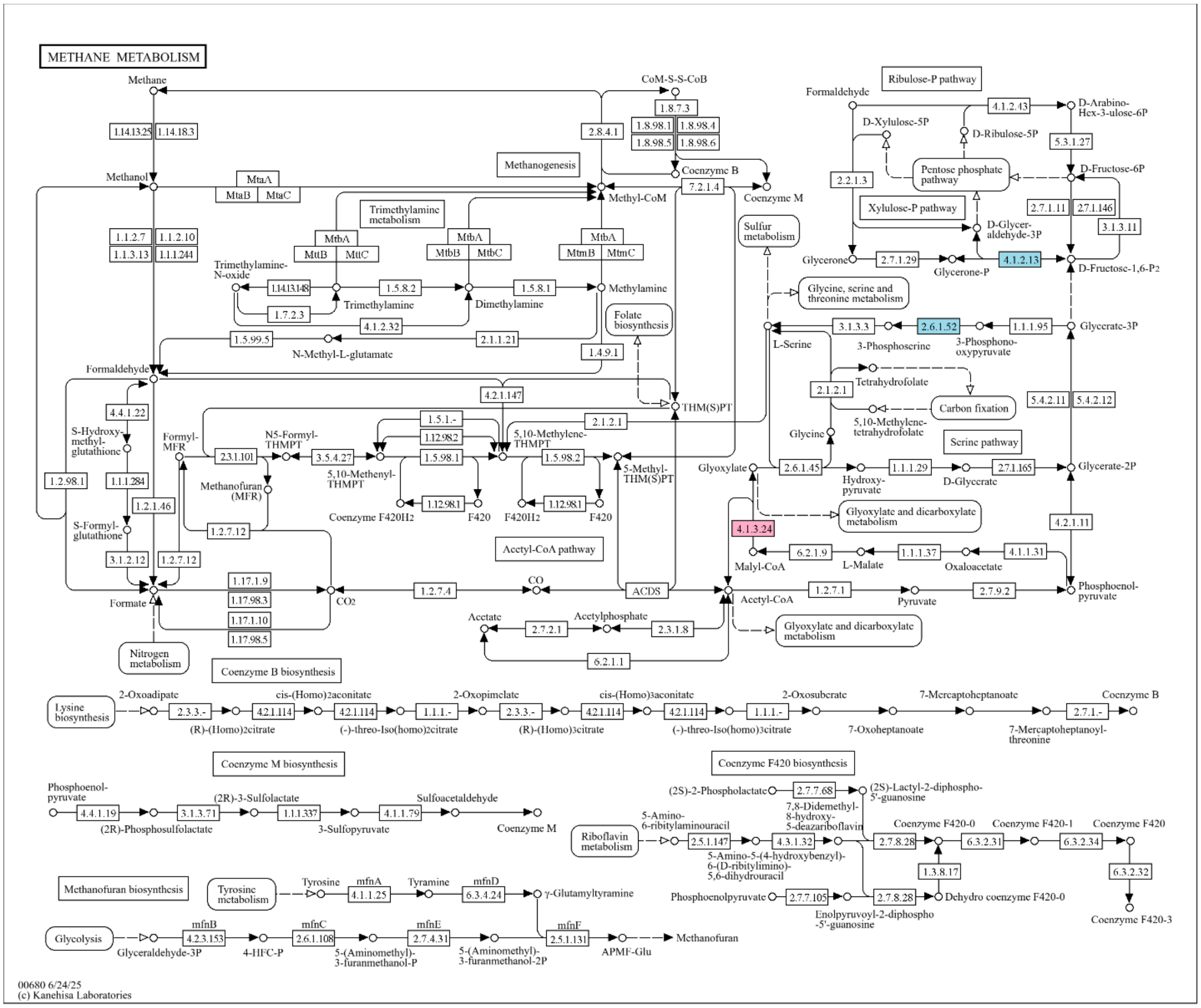

Figure S28. KEGG pathway "Methane metabolism" (map00680), delineating enzyme(s) and/or enzymatic constituents manifesting high abundance (demarcated by light blue boxes) within the intestinal microbial consortium of female C57BL/6 mice subjected to a fiber-devoid (NF) nutritional paradigm, juxtaposed against experimental cohorts administered either fiber-replete (F) or fiber-devoid diets augmented with exogenous short-chain fatty acids (NF-SCFA). These biocatalytic entities orchestrate the biosynthetic transformation of alternative non-polysaccharide substrates—including proteinaceous amino acid moieties and monosaccharide metabolites synthesized by the gut microbiome, which subsequently undergo anaerobic fermentative catabolism, culminating in the generation of short-chain fatty acids. EC 2.6.1.52 = phosphoserine aminotransferase, EC 4.1.3.24 = malyl-CoA lyase, EC 4.1.2.13 = fructose-bisphosphate aldolase, class I & II. Enzymes exhibiting pronounced enrichment in fiber-replete (F) dietary cohort versus fiber-devoid (NF) or exogenous SCFA-supplemented (NF-SCFA) groups are demarcated by pink boxes, whereas enzymes demonstrating pronounced enrichment in fiber-devoid (NF) cohort relative to fiber-replete (F) or SCFA-supplemented (NF-SCFA) groups are demarcated by light blue boxes.

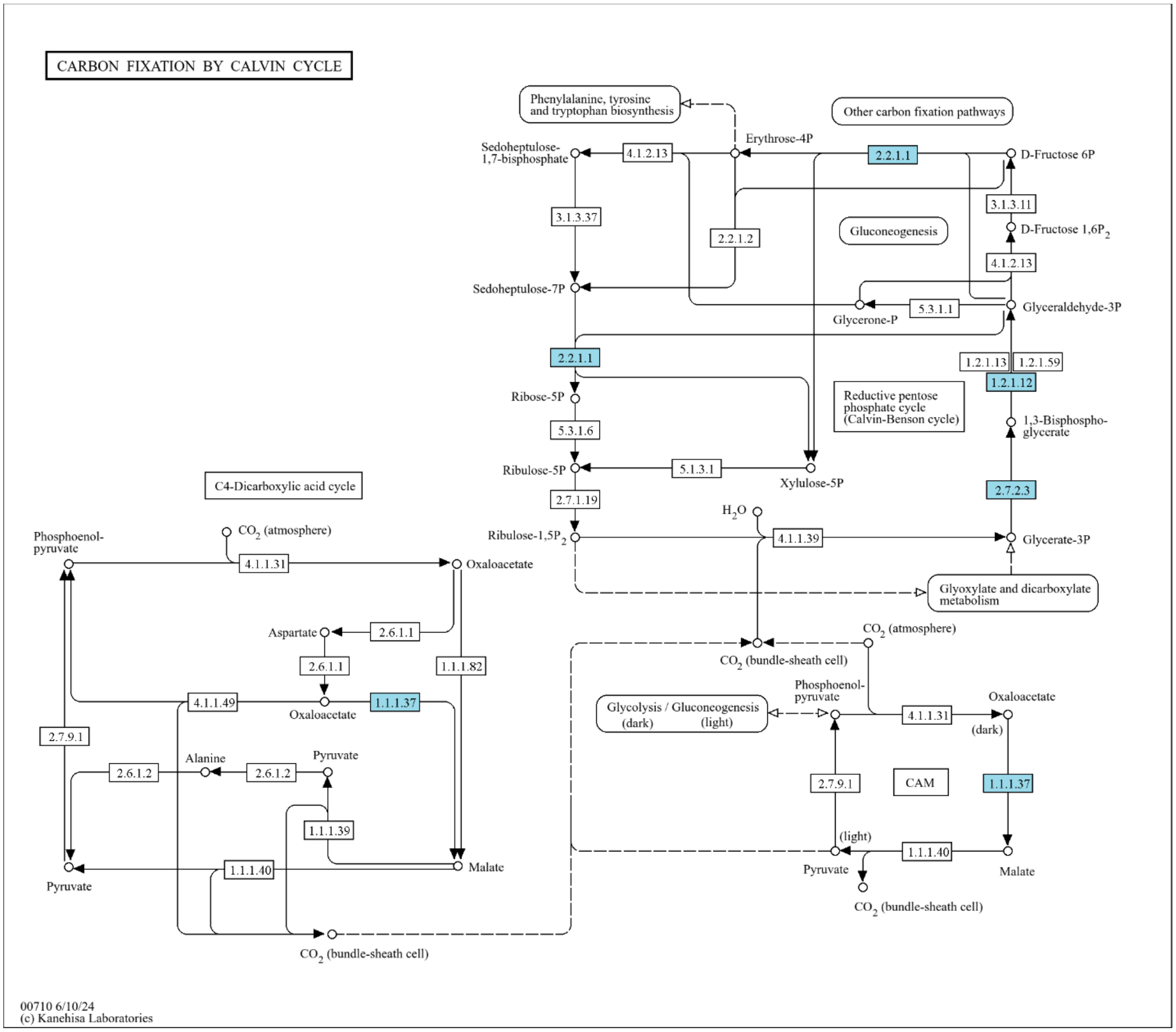

Figure S29. KEGG pathway "Carbon fixation by Calvin cycle" (map00710), delineating enzyme(s) and/or enzymatic constituents manifesting high abundance (demarcated by light blue boxes) within the intestinal microbial consortium of female C57BL/6 mice subjected to a fiber-devoid (NF) nutritional paradigm, juxtaposed against experimental cohorts administered either fiber-replete (F) or fiber-devoid diets augmented with exogenous short-chain fatty acids (NF-SCFA). These biocatalytic entities orchestrate the biosynthetic transformation of alternative non-polysaccharide substrates—including proteinaceous amino acid moieties and monosaccharide metabolites synthesized by the gut microbiome, which subsequently undergo anaerobic fermentative catabolism, culminating in the generation of short-chain fatty acids. EC 2.7.2.3 = phosphoglycerate kinase, EC 1.2.1.12 = glyceraldehyde-3-phosphate dehydrogenase, EC 1.1.1.37 = malate dehydrogenase, EC 2.2.1.1 = transketolase. Enzymes exhibiting pronounced enrichment in fiber-replete (F) dietary cohort versus fiber-devoid (NF) or exogenous SCFA-supplemented (NF-SCFA) groups are demarcated by pink boxes, whereas enzymes demonstrating pronounced enrichment in fiber-devoid (NF) cohort relative to fiber-replete (F) or SCFA-supplemented (NF-SCFA) groups are demarcated by light blue boxes.

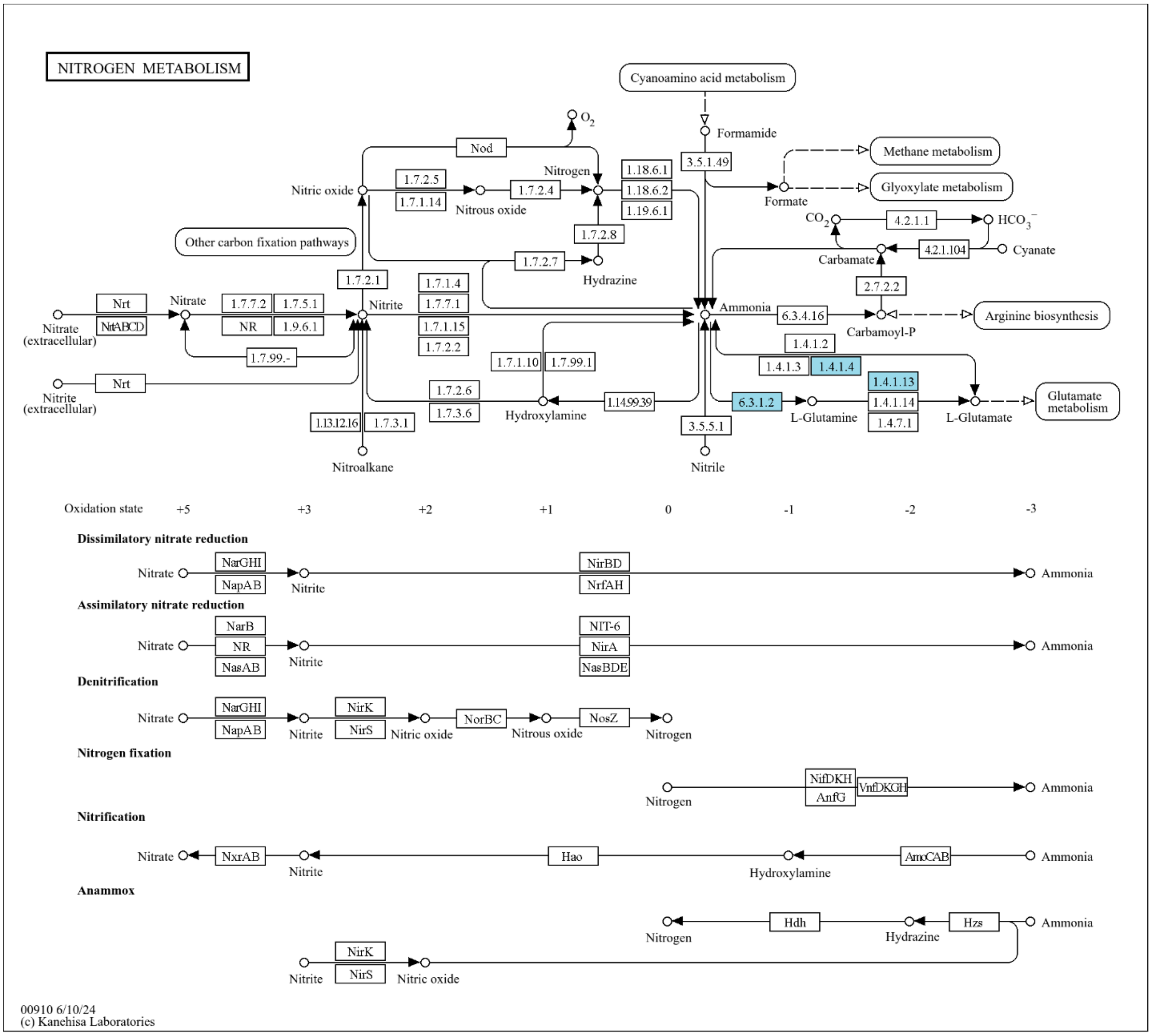

Figure S30. KEGG pathway "Nitrogen metabolism" (map00910), delineating enzyme(s) and/or enzymatic constituents manifesting high abundance (demarcated by light blue boxes) within the intestinal microbial consortium of female C57BL/6 mice subjected to a fiber-devoid (NF) nutritional paradigm, juxtaposed against experimental cohorts administered either fiber-replete (F) or fiber-devoid diets augmented with exogenous short-chain fatty acids (NF-SCFA). These biocatalytic entities orchestrate the biosynthetic transformation of alternative non-polysaccharide substrates—including proteinaceous amino acid moieties and monosaccharide metabolites synthesized by the gut microbiome, which subsequently undergo anaerobic fermentative catabolism, culminating in the generation of short-chain fatty acids. EC 1.4.1.13 = glutamate synthase (NADPH/NADH) small chain, EC 6.3.1.2 = glutamine synthetase. Enzymes exhibiting pronounced enrichment in fiber-replete (F) dietary cohort versus fiber-devoid (NF) or exogenous SCFA-supplemented (NF-SCFA) groups are demarcated by pink boxes, whereas enzymes demonstrating pronounced enrichment in fiber-devoid (NF) cohort relative to fiber-replete (F) or SCFA-supplemented (NF-SCFA) groups are demarcated by light blue boxes.

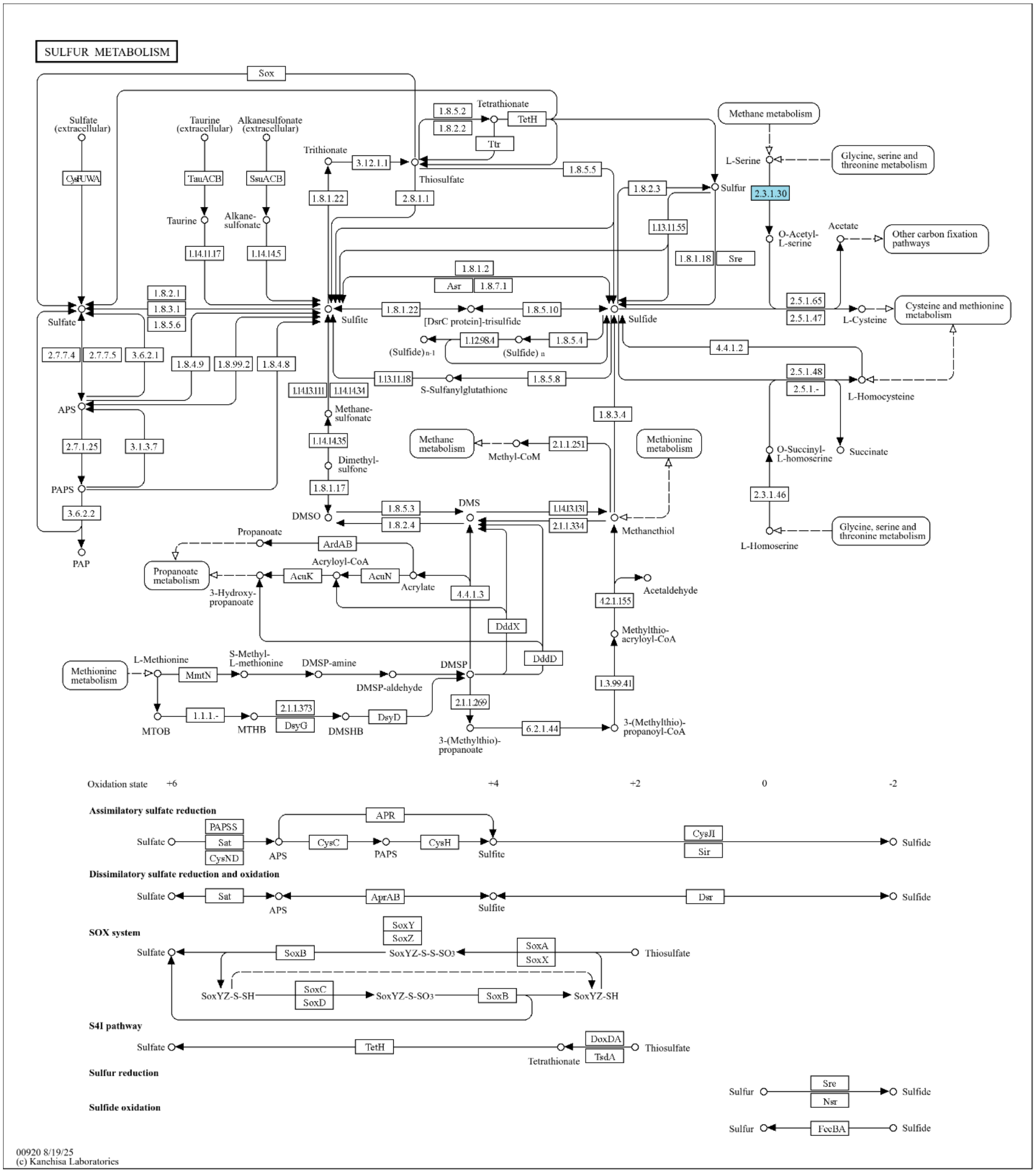

Figure S31. KEGG pathway "Sulfur metabolism" (map00920), delineating enzyme(s) and/or enzymatic constituents manifesting high abundance (demarcated by light blue boxes) within the intestinal microbial consortium of female C57BL/6 mice subjected to a fiber-devoid (NF) nutritional paradigm, juxtaposed against experimental cohorts administered either fiber-replete (F) or fiber-devoid diets augmented with exogenous short-chain fatty acids (NF-SCFA). These biocatalytic entities orchestrate the biosynthetic transformation of alternative non-polysaccharide substrates—including proteinaceous amino acid moieties and monosaccharide metabolites synthesized by the gut microbiome, which subsequently undergo anaerobic fermentative catabolism, culminating in the generation of short-chain fatty acids. EC 2.3.1.30 = serine acetyltransferase. Enzymes exhibiting pronounced enrichment in fiber-replete (F) dietary cohort versus fiber-devoid (NF) or exogenous SCFA-supplemented (NF-SCFA) groups are demarcated by pink boxes, whereas enzymes demonstrating pronounced enrichment in fiber-devoid (NF) cohort relative to fiber-replete (F) or SCFA-supplemented (NF-SCFA) groups are demarcated by light blue boxes.

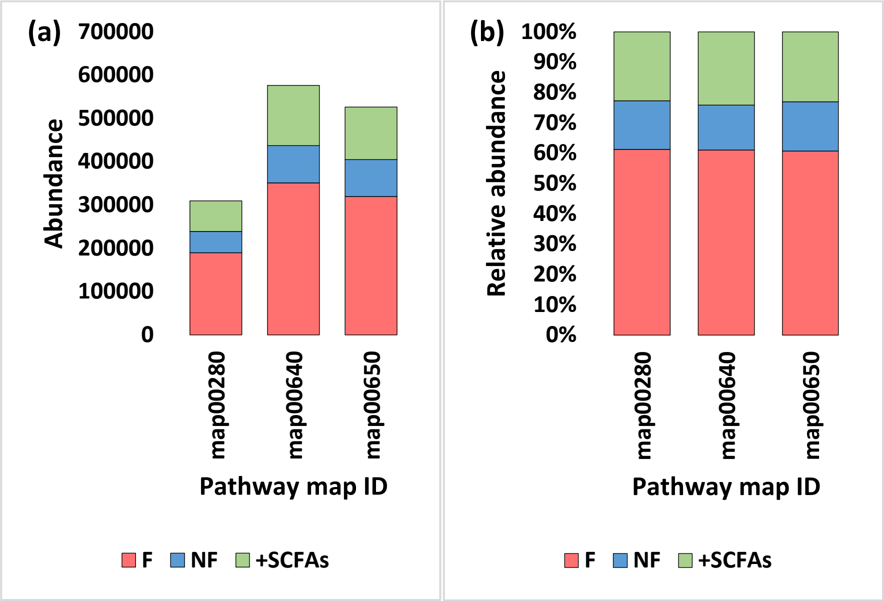

Figure S32. Abundance (a) and relative abundance (b) of preferential KEGG metabolic pathways within the gut microbiome of female C57BL/6 mice, as defined by the distribution of highly prevalent enzymes or enzymatic constituents in fiber-replete (F), and fiber-devoid (NF) cohorts compared to that fed a fiber-devoid diet supplemented with exogenous SCFAs (+SCFA). Comprehensive details for enzymatic classifications, abundance, and pathway mapping are available in Tables S6 and S7.

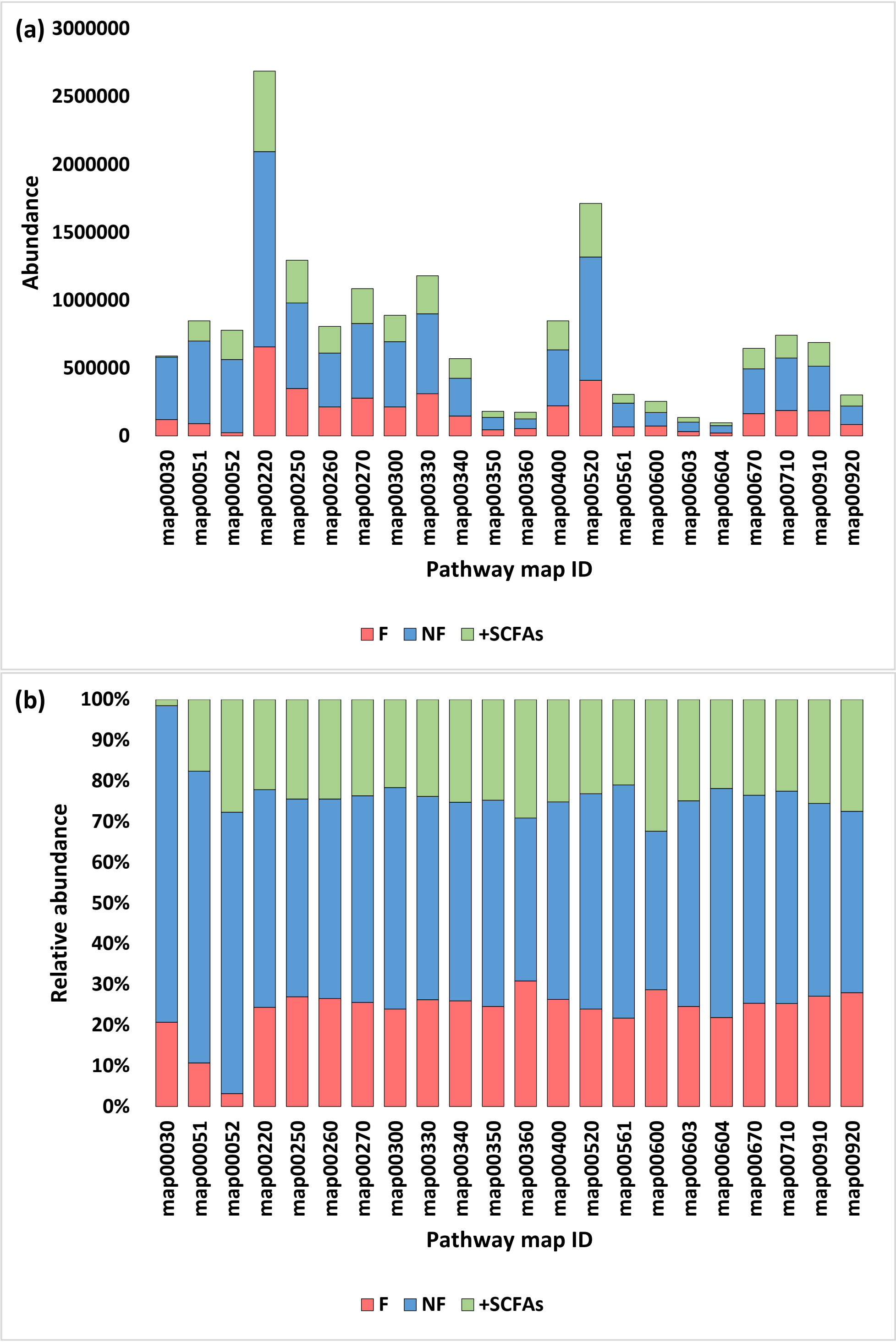

Figure S33. Abundance (a) and relative abundance (b) of preferential KEGG metabolic pathways within the gut microbiome of female C57BL/6 mice, as determined by the distribution of highly prevalent enzymes or enzymatic constituents in fiber-devoid (NF) cohort versus those maintained on a fiber-replete (F) diet or on a fiber-devoid diet supplemented with exogenous SCFAs (+SCFA). Detailed enzyme classifications, abundance, and pathway assignments are provided in Tables S6 and S7.

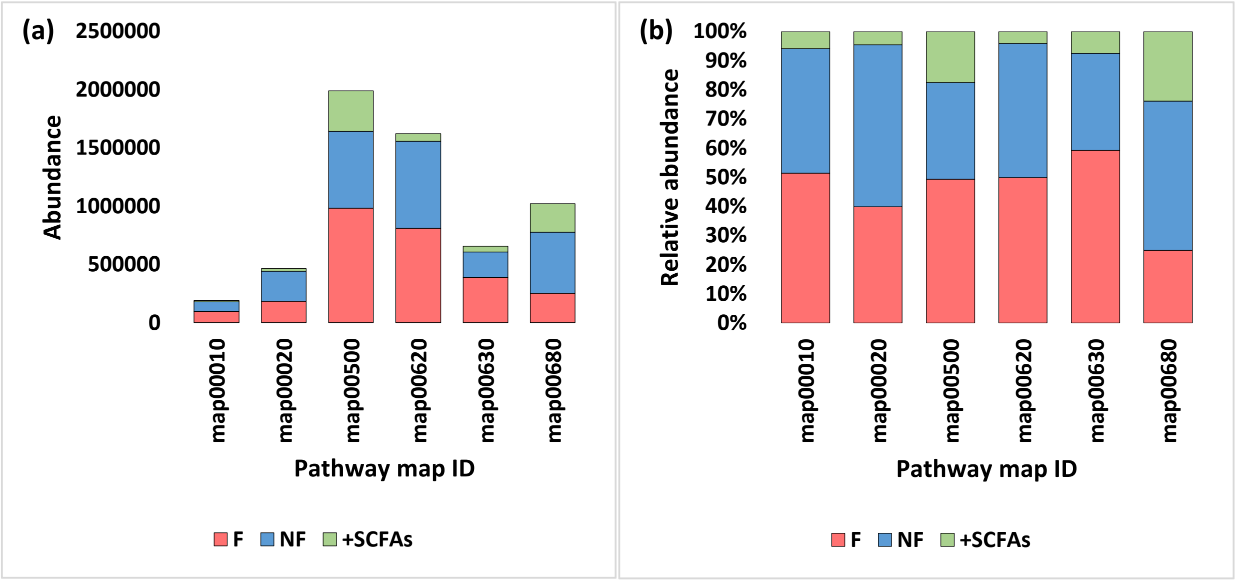

Figure S34. Abundance (a) and relative abundance (b) of preferential KEGG metabolic pathways within the gut microbiome of female C57BL/6 mice, as determined by the distribution of highly prevalent enzymes or enzymatic constituents in fiber-deplete and fiber-devoid (NF) cohorts versus that maintained on a fiber-devoid diet supplemented with exogenous SCFAs (+SCFA). Detailed enzyme classifications, abundance, and pathway assignments are provided in Tables S6 and S7.

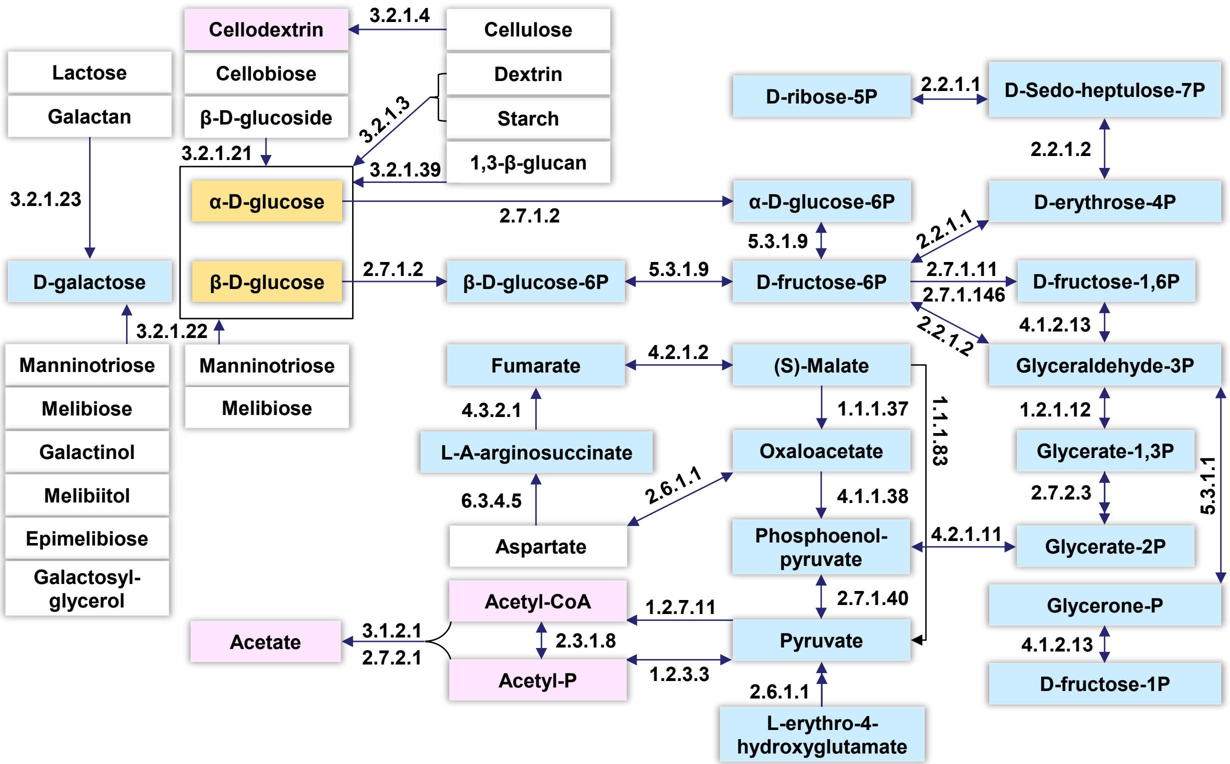

Figure S35. Dietary fiber-dependent metabolic pathways driving pyruvate metabolism and acetate biosyntheis in the gut microbiota of female C57BL/6 mice. This diagram illustrates the metabolic divergence manifested by gut microbiota across three distinct nutritional paradigms: fiber-replete (F), fiber-devoid (NF), and fiber-devoid with exogenous short-chain fatty acid supplementation (NF-SCFA). The microbiota maintained under fiber-devoid conditions preferentially engage metabolic conduits culminating in pyruvate biosynthesis, whereas those sustained on fiber-replete regimens predominantly activate enzymatic pathways directing pyruvate conversion toward acetate production. Color-stratified metabolic nodes designate primary metabolic substrates (white boxes), intermediary metabolic junctions, and terminal fermentation products, each annotated according to their relative abundance across treatment cohorts. Blue-demarcated boxes represent metabolites manifesting substantially elevated concentrations within the fiber-devoid (NF) microbiota relative to alternative treatment cohorts, whereas pink-demarcated boxes identify metabolites exhibiting preferentially augmented abundance exclusively within the fiber-replete (F) microbiota compared with both fiber-devoid (NF) and fiber-devoid-supplemented (NF-SCFA) cohorts. Glucose, marked in orange, exhibits comparable enrichment under both fiber-replete and fiber-devoid nutritional conditions, reflecting its indispensable role as a fundamental glycolytic hub despite differential enzymatic mechanisms and catabolic specialization characterizing each dietary cohort. Secondary metabolic endpoints accumulated under fiber-devoid conditions—including D-ribose-5-phosphate, D-galactose, and fumarate—represent metabolic intermediates that fail to undergo the additional transformative steps requisite for SCFAs biosynthesis, thereby exemplifying the metabolic incompleteness of non-polysaccharide fermentation pathways. Comprehensive metabolomic characterization and supporting analytical data are detailed in Table S6 and Figures S1-S7, S9, S12, S14-S26, S28, and S29.
