# Supplementary material for: Dietary Fiber-Associated Differences in Gut Microbial Community Composition and Predicted Short-Chain Fatty Acid-Related Functional Potential: An In Silico Re-Analysis of 16S rRNA Data
Source: Int J Mol Sci. 2026 Sep 2;27(17):7851. doi: 10.3390/ijms27177851 (PMC13566324; doi:10.3390/ijms27177851)
Supplement: Supplementary file 1 [file ijms-27-07851-s001.zip › Table S2.pdf]

Table S2. Pairwise and overall PERMANOVA, ANOSIM, and Jaccard-based statistical comparisons of gut microbial community composition across dietary cohorts.

**Pairwise comparison**	**pseudo-F**	**Raw p-value**	**BH q-value**
Fiber vs. No-Fiber	23.209	0.009	0.012
Fiber vs. NF-SCFA	27.366	0.008	0.012
No-Fiber vs. NF-SCFA	71.383	0.012	0.012
