# Supplementary material for: Dietary Fiber-Associated Differences in Gut Microbial Community Composition and Predicted Short-Chain Fatty Acid-Related Functional Potential: An In Silico Re-Analysis of 16S rRNA Data
Source: Int J Mol Sci. 2026 Sep 2;27(17):7851. doi: 10.3390/ijms27177851 (PMC13566324; doi:10.3390/ijms27177851)
Supplement: Supplementary file 1 [file ijms-27-07851-s001.zip › Table S8.pdf]

Table S8. GenBank (NCBI) accession numbers for 16S rRNA gene sequencing datasets derived from fecal samples of female C57BL/6 mice fed fiber-rich (F), no-fiber (NF), or no-fiber + exogenous short-chain fatty acid (NF-SCFA) diets. R = regular fiber diet (physiological SCFA production), L = low/no-fiber diet (deficient in SCFAs), LR = low/no-fiber diet supplemented with exogenous SCFAs, NC = non-immunized control.

Sample name	Accession no.
F-R1	SAMN13226853
F-R2	SAMN13226854
F-R3	SAMN13226855
F-R4	SAMN13226856
F-NC	SAMN13226857
NF-L1	SAMN13226858
NF-L2	SAMN13226859
NF-L3	SAMN13226860
NF-L4	SAMN13226861
NF-NC	SAMN13226862
NF-SCFA-LR1	SAMN13226863
NF-SCFA-LR2	SAMN13226864
NF-SCFA-LR3	SAMN13226865
NF-SCFA-LR4	SAMN13226866
NF-SCFA-NC	SAMN13226867
